# Supplementary material for: Impacts of observation frequency on proximity contact data and modeled transmission dynamics
Source: PLoS Comput Biol. 2023 Feb 27;19(2):e1010917. doi: 10.1371/journal.pcbi.1010917 (PMC9997969; doi:10.1371/journal.pcbi.1010917)
Supplement: S1 Table — It contains two comma-separated values (CSV) files, one for each of the Snapshot and the Upperbound downsampling method. Each CSV file contains details of the corresponding Welch’s t-test, where block names are underscore-concatenated strings of the underlying population and the type of disease/pathogens. (ZIP) [file pcbi.1010917.s001.zip › Welchs-t-test-on-observation-intervals/Welchs-t-test-on-observation-intervals-snapshot.pdf]

number of t-tests per block: 21 (combination 2 out of 7, that is 7*6/2)
Bonferroni-corrected critical value: 0.00238 (0.05 / 21)
Lines with red background-color are cases rejected by Bonferroni-corrected t-test

blocks	obs.interval.x	obs.interval.y	t	df	p.value	conf.low	conf.high
shed1_covid19	5	10	-0.74547	2329.4	0.45606	-0.57095	0.25642
shed1_covid19	5	30	0.13934	2334.5	0.88920	-0.37992	0.43804
shed1_covid19	5	60	0.83778	2330.6	0.40224	-0.22345	0.55678
shed1_covid19	5	90	-0.24312	2337.9	0.80793	-0.44941	0.35027
shed1_covid19	5	180	1.47516	2331.6	0.14031	-0.09683	0.68487
shed1_covid19	5	360	9.15517	2019.8	0.00000	1.24450	1.92302
shed1_covid19	10	30	0.86689	2336.9	0.38609	-0.23516	0.60781
shed1_covid19	10	60	1.57545	2306.7	0.11529	-0.07927	0.72714
shed1_covid19	10	90	0.51181	2327.8	0.60884	-0.30493	0.52032
shed1_covid19	10	180	2.19095	2308.7	0.02855	0.04737	0.85520
shed1_covid19	10	360	9.63878	1948.9	0.00000	1.38678	2.09527
shed1_covid19	30	60	0.67738	2317.1	0.49823	-0.26076	0.53597
shed1_covid19	30	90	-0.37803	2333.4	0.70544	-0.48653	0.32927
shed1_covid19	30	180	1.30192	2318.8	0.19307	-0.13413	0.66404
shed1_covid19	30	360	8.74339	1974.3	0.00000	1.20598	1.90342
shed1_covid19	60	90	-1.09013	2332.0	0.27577	-0.60522	0.17274
shed1_covid19	60	180	0.65765	2338.0	0.51083	-0.25238	0.50708
shed1_covid19	60	360	8.51477	2084.9	0.00000	1.09071	1.74348
shed1_covid19	90	180	1.72887	2332.9	0.08397	-0.04613	0.73331
shed1_covid19	90	360	9.47809	2026.2	0.00000	1.29538	1.97129
shed1_covid19	180	360	7.72878	2080.4	0.00000	0.96248	1.61700
shed1_covid19alpha	5	10	0.01288	2337.5	0.98972	-0.51701	0.52385
shed1_covid19alpha	5	30	-0.27523	2336.3	0.78316	-0.59721	0.45020
shed1_covid19alpha	5	60	1.54842	2331.3	0.12166	-0.10589	0.90077
shed1_covid19alpha	5	90	1.35122	2330.2	0.17676	-0.15621	0.84852
shed1_covid19alpha	5	180	3.15131	2306.2	0.00165	0.29669	1.27425
shed1_covid19alpha	5	360	11.70258	1923.0	0.00000	2.12087	2.97485
shed1_covid19alpha	10	30	-0.28601	2337.6	0.77489	-0.60433	0.45048
shed1_covid19alpha	10	60	1.52344	2327.2	0.12778	-0.11316	0.90120
shed1_covid19alpha	10	90	1.32768	2325.9	0.18441	-0.16348	0.84895
shed1_covid19alpha	10	180	3.11236	2298.2	0.00188	0.28931	1.27480
shed1_covid19alpha	10	360	11.56412	1906.5	0.00000	2.11292	2.97597
shed1_covid19alpha	30	60	1.80889	2323.0	0.07060	-0.03960	0.98148
shed1_covid19alpha	30	90	1.61493	2321.5	0.10646	-0.08993	0.92924
shed1_covid19alpha	30	180	3.39469	2290.8	0.00070	0.36277	1.35518
shed1_covid19alpha	30	360	11.80589	1892.5	0.00000	2.18590	3.05683
shed1_covid19alpha	60	90	-0.20581	2338.0	0.83696	-0.53990	0.43734
shed1_covid19alpha	60	180	1.60315	2328.4	0.10904	-0.08661	0.86268
shed1_covid19alpha	60	360	10.26805	1985.5	0.00000	1.73970	2.56115
shed1_covid19alpha	90	180	1.81896	2329.6	0.06905	-0.03430	0.91293
shed1_covid19alpha	90	360	10.54340	1990.3	0.00000	1.79217	2.61124
shed1_covid19alpha	180	360	8.80031	2060.3	0.00000	1.36965	2.15514
shed1_covid19beta	5	10	-0.26775	2336.1	0.78892	-0.57628	0.43782
shed1_covid19beta	5	30	-0.44216	2333.0	0.65841	-0.62712	0.39635
shed1_covid19beta	5	60	1.28683	2334.0	0.19828	-0.16836	0.81109
shed1_covid19beta	5	90	1.21114	2331.5	0.22597	-0.18627	0.78798
shed1_covid19beta	5	180	3.22663	2305.9	0.00127	0.30508	1.25047
shed1_covid19beta	5	360	11.57359	1941.2	0.00000	2.03306	2.86266
shed1_covid19beta	10	30	-0.17445	2337.3	0.86153	-0.56497	0.47266
shed1_covid19beta	10	60	1.54078	2326.6	0.12351	-0.10652	0.88772
shed1_covid19beta	10	90	1.46744	2322.8	0.14239	-0.12447	0.86464
shed1_covid19beta	10	180	3.45782	2289.3	0.00055	0.36665	1.32736
shed1_covid19beta	10	360	11.65620	1908.4	0.00000	2.09358	2.94061
shed1_covid19beta	30	60	1.70645	2320.2	0.08806	-0.06515	0.93865
shed1_covid19beta	30	90	1.63458	2315.5	0.10227	-0.08312	0.91560
shed1_covid19beta	30	180	3.60911	2277.4	0.00031	0.40786	1.37846
shed1_covid19beta	30	360	11.71510	1888.2	0.00000	2.13414	2.99236
shed1_covid19beta	60	90	-0.08437	2337.7	0.93277	-0.49729	0.45627
shed1_covid19beta	60	180	1.93713	2324.3	0.05285	-0.00562	0.91844
shed1_covid19beta	60	360	10.35873	1989.6	0.00000	1.72390	2.52909
shed1_covid19beta	90	180	2.03637	2328.0	0.04183	0.01765	0.93619
shed1_covid19beta	90	360	10.54175	2002.7	0.00000	1.74759	2.54643
shed1_covid19beta	180	360	8.58100	2079.0	0.00000	1.28840	2.05177
shed1_covid19delta	5	10	-1.23065	2333.1	0.21858	-1.27013	0.29064
shed1_covid19delta	5	30	-0.29064	2338.0	0.77135	-0.87403	0.64839
shed1_covid19delta	5	60	1.41221	2332.4	0.15802	-0.20825	1.28004
shed1_covid19delta	5	90	1.10770	2332.6	0.26811	-0.32393	1.16495
shed1_covid19delta	5	180	4.57355	2289.3	0.00001	0.94863	2.37273
shed1_covid19delta	5	360	16.29796	1843.6	0.00000	4.52312	5.76064
shed1_covid19delta	10	30	0.94834	2332.5	0.34306	-0.40248	1.15633
shed1_covid19delta	10	60	2.63689	2317.1	0.00842	0.26290	1.78838
shed1_covid19delta	10	90	2.33935	2317.5	0.01940	0.14722	1.67329
shed1_covid19delta	10	180	5.76513	2256.0	0.00000	1.41896	2.88190
shed1_covid19delta	10	360	17.23063	1793.9	0.00000	4.99060	6.27265
shed1_covid19delta	30	60	1.71188	2332.9	0.08705	-0.09440	1.39183
shed1_covid19delta	30	90	1.40683	2333.1	0.15961	-0.21008	1.27675
shed1_covid19delta	30	180	4.89163	2290.9	0.00000	1.06253	2.48448
shed1_covid19delta	30	360	16.68887	1846.5	0.00000	4.63718	5.87222
shed1_covid19delta	60	90	-0.31169	2338.0	0.75530	-0.84131	0.61054
shed1_covid19delta	60	180	3.18436	2316.0	0.00147	0.43212	1.81745
shed1_covid19delta	60	360	15.14802	1898.8	0.00000	4.00965	5.20232
shed1_covid19delta	90	180	3.50940	2315.6	0.00046	0.54719	1.93316
shed1_covid19delta	90	360	15.51780	1897.8	0.00000	4.12466	5.31808
shed1_covid19delta	180	360	12.28486	2012.2	0.00000	2.92546	4.03693
shed1_diphtheria	5	10	-1.52420	2332.9	0.12759	-1.06120	0.13300
shed1_diphtheria	5	30	0.30236	2336.8	0.76241	-0.48761	0.66539
shed1_diphtheria	5	60	0.86043	2337.8	0.38964	-0.32578	0.83518
shed1_diphtheria	5	90	-0.06582	2337.8	0.94753	-0.60534	0.56602
shed1_diphtheria	5	180	2.32666	2316.5	0.02007	0.10384	1.21753
shed1_diphtheria	5	360	11.54431	1944.3	0.00000	2.36619	3.33467
shed1_diphtheria	10	30	1.83574	2326.8	0.06652	-0.03773	1.14371
shed1_diphtheria	10	60	2.37056	2330.9	0.01784	0.12419	1.31341
shed1_diphtheria	10	90	1.45335	2334.7	0.14626	-0.15524	1.04413
shed1_diphtheria	10	180	3.85909	2291.7	0.00012	0.55323	1.69635
shed1_diphtheria	10	360	12.97254	1890.8	0.00000	2.81343	3.81563
shed1_diphtheria	30	60	0.56655	2337.5	0.57108	-0.40811	0.73973
shed1_diphtheria	30	90	-0.36752	2335.6	0.71326	-0.68772	0.47063
shed1_diphtheria	30	180	2.03869	2325.4	0.04159	0.02179	1.12180
shed1_diphtheria	30	360	11.36953	1970.9	0.00000	2.28519	3.23789
shed1_diphtheria	60	90	-0.92261	2337.3	0.35631	-0.85750	0.30878
shed1_diphtheria	60	180	1.43659	2320.2	0.15097	-0.14819	0.96016
shed1_diphtheria	60	360	10.57989	1954.6	0.00000	2.11456	3.07689
shed1_diphtheria	90	180	2.38403	2312.4	0.01720	0.12072	1.23996
shed1_diphtheria	90	360	11.54801	1933.9	0.00000	2.38266	3.35751
shed1_diphtheria	180	360	9.49349	2056.9	0.00000	1.73740	2.64209
shed1_fifth	5	10	0.19076	2338.0	0.84873	-0.19829	0.24103
shed1_fifth	5	30	-1.91826	2328.7	0.05520	-0.44939	0.00495
shed1_fifth	5	60	-0.73725	2337.9	0.46105	-0.30342	0.13761
shed1_fifth	5	90	-2.17827	2332.4	0.02949	-0.47587	-0.02498
shed1_fifth	5	180	-0.41152	2334.9	0.68073	-0.27102	0.17700
shed1_fifth	5	360	4.78163	2165.9	0.00000	0.27931	0.66770
shed1_fifth	10	30	-2.10449	2328.1	0.03544	-0.47057	-0.01661
shed1_fifth	10	60	-0.92810	2337.9	0.35345	-0.32459	0.11605
shed1_fifth	10	90	-2.36617	2332.0	0.01805	-0.49705	-0.04654
shed1_fifth	10	180	-0.59909	2334.5	0.54917	-0.29219	0.15544
shed1_fifth	10	360	4.57115	2167.8	0.00001	0.25817	0.64611
shed1_fifth	30	60	1.19922	2330.3	0.23056	-0.08850	0.36713
shed1_fifth	30	90	-0.23781	2337.5	0.81205	-0.26079	0.20438
shed1_fifth	30	180	1.48618	2336.3	0.13737	-0.05598	0.40640
shed1_fifth	30	360	6.73961	2096.8	0.00000	0.49328	0.89817
shed1_fifth	60	90	-1.45299	2333.7	0.14636	-0.39361	0.05857
shed1_fifth	60	180	0.31334	2335.8	0.75405	-0.18876	0.26055
shed1_fifth	60	360	5.59729	2159.7	0.00000	0.36147	0.75135

blocks	obs.interval.x	obs.interval.y	t	df	p.value	conf.low	conf.high
shed1_fifth	90	180	1.73817	2337.6	0.08231	-0.02608	0.43291
shed1_fifth	90	360	7.08064	2113.0	0.00000	0.52343	0.92444
shed1_fifth	180	360	5.13249	2126.6	0.00000	0.32163	0.71940
shed1_flu	5	10	0.29756	2337.5	0.76607	-0.22934	0.31139
shed1_flu	5	30	0.31386	2336.1	0.75366	-0.22876	0.31594
shed1_flu	5	60	1.24101	2334.5	0.21473	-0.09669	0.43003
shed1_flu	5	90	-0.78153	2330.0	0.43457	-0.38691	0.16639
shed1_flu	5	180	3.14218	2285.7	0.00170	0.15069	0.65102
shed1_flu	5	360	9.34361	2085.9	0.00000	0.87047	1.33294
shed1_flu	10	30	0.01833	2337.5	0.98537	-0.27171	0.27683
shed1_flu	10	60	0.92852	2331.4	0.35323	-0.13971	0.39099
shed1_flu	10	90	-1.06504	2333.4	0.28697	-0.42983	0.12726
shed1_flu	10	180	2.79720	2275.8	0.00520	0.10757	0.61209
shed1_flu	10	360	8.90852	2069.5	0.00000	0.82719	1.29418
shed1_flu	30	60	0.90271	2327.4	0.36677	-0.14429	0.39044
shed1_flu	30	90	-1.07567	2335.9	0.28218	-0.43431	0.12662
shed1_flu	30	180	2.75412	2265.3	0.00593	0.10288	0.61165
shed1_flu	30	360	8.80069	2053.0	0.00000	0.82233	1.29391
shed1_flu	60	90	-1.99835	2316.1	0.04580	-0.54867	-0.00518
shed1_flu	60	180	1.87651	2308.5	0.06071	-0.01054	0.47892
shed1_flu	60	360	8.13748	2129.3	0.00000	0.70970	1.16038
shed1_flu	90	180	3.87010	2241.0	0.00011	0.25213	0.77010
shed1_flu	90	360	9.87269	2018.1	0.00000	0.97122	1.45271
shed1_flu	180	360	6.55217	2241.7	0.00000	0.49109	0.91062
shed1_mers	5	10	-0.22523	2334.9	0.82182	-0.14933	0.11856
shed1_mers	5	30	0.15596	2334.3	0.87608	-0.11870	0.13921
shed1_mers	5	60	1.21052	2333.8	0.22620	-0.04928	0.20825
shed1_mers	5	90	0.03796	2337.5	0.96972	-0.12989	0.13502
shed1_mers	5	180	1.58202	2320.2	0.11378	-0.02436	0.22778
shed1_mers	5	360	4.71394	2186.3	0.00000	0.16422	0.39818
shed1_mers	10	30	0.38247	2324.6	0.70215	-0.10583	0.15711
shed1_mers	10	60	1.41715	2323.5	0.15657	-0.03641	0.22615
shed1_mers	10	90	0.26091	2336.9	0.79419	-0.11695	0.15285
shed1_mers	10	180	1.78499	2302.8	0.07439	-0.01155	0.24573
shed1_mers	10	360	4.85720	2148.5	0.00000	0.17684	0.41632
shed1_mers	30	60	1.07588	2338.0	0.28209	-0.05695	0.19542
shed1_mers	30	90	-0.11608	2331.2	0.90760	-0.13764	0.12226
shed1_mers	30	180	1.45287	2332.6	0.14639	-0.03198	0.21489
shed1_mers	30	360	4.65527	2224.2	0.00000	0.15681	0.38507
shed1_mers	60	90	-1.16249	2330.4	0.24516	-0.20668	0.05284
shed1_mers	60	180	0.35361	2333.2	0.72366	-0.10101	0.14546
shed1_mers	60	360	3.47235	2227.0	0.00053	0.08779	0.31563
shed1_mers	90	180	1.52980	2313.8	0.12620	-0.02794	0.22624
shed1_mers	90	360	4.62764	2171.4	0.00000	0.16056	0.39671
shed1_mers	180	360	3.17487	2264.7	0.00152	0.06862	0.29035
shed1_sars	5	10	0.02663	2337.7	0.97876	-0.37254	0.38280
shed1_sars	5	30	-0.37773	2337.8	0.70567	-0.45510	0.30810
shed1_sars	5	60	0.26712	2337.3	0.78940	-0.32519	0.42775
shed1_sars	5	90	-0.02653	2338.0	0.97884	-0.38422	0.37397
shed1_sars	5	180	1.79634	2325.9	0.07257	-0.03079	0.70258
shed1_sars	5	360	10.08768	1975.3	0.00000	1.31648	1.95189
shed1_sars	10	30	-0.40621	2337.0	0.68462	-0.45823	0.30096
shed1_sars	10	60	0.24171	2337.9	0.80902	-0.32828	0.42059
shed1_sars	10	90	-0.05334	2337.9	0.95747	-0.38733	0.36682
shed1_sars	10	180	1.77904	2329.2	0.07536	-0.03383	0.69537
shed1_sars	10	360	10.13289	1987.8	0.00000	1.31377	1.94435
shed1_sars	30	60	0.64668	2336.3	0.51790	-0.25361	0.50319
shed1_sars	30	90	0.35192	2337.6	0.72493	-0.31263	0.44939
shed1_sars	30	180	2.17767	2322.3	0.02953	0.04074	0.77807
shed1_sars	30	360	10.46614	1963.6	0.00000	1.38770	2.02768
shed1_sars	60	90	-0.29430	2337.5	0.76855	-0.43228	0.31946
shed1_sars	60	180	1.53605	2330.9	0.12466	-0.07874	0.64797
shed1_sars	60	360	9.89106	1995.4	0.00000	1.26905	1.89676
shed1_sars	90	180	1.82682	2326.9	0.06785	-0.02505	0.70710
shed1_sars	90	360	10.14192	1979.0	0.00000	1.32232	1.95631
shed1_sars	180	360	8.42981	2059.6	0.00000	0.99626	1.60033
shed2_covid19	5	10	0.19408	1917.9	0.84613	-0.15175	0.18509
shed2_covid19	5	30	0.60385	1909.6	0.54601	-0.11239	0.21239
shed2_covid19	5	60	1.35722	1864.3	0.17487	-0.04775	0.26233
shed2_covid19	5	90	0.20484	1917.1	0.83772	-0.15184	0.18725
shed2_covid19	5	180	1.63225	1865.7	0.10280	-0.02603	0.28437
shed2_covid19	5	360	-0.98637	1884.2	0.32408	-0.27082	0.08957
shed2_covid19	10	30	0.40069	1907.3	0.68869	-0.12982	0.19648
shed2_covid19	10	60	1.14054	1858.9	0.25421	-0.06521	0.24646
shed2_covid19	10	90	0.01200	1917.7	0.99043	-0.16923	0.17131
shed2_covid19	10	180	1.41440	1860.5	0.15741	-0.04350	0.26850
shed2_covid19	10	360	-1.16336	1888.3	0.24483	-0.28817	0.07358
shed2_covid19	30	60	0.75259	1897.2	0.45179	-0.09201	0.20659
shed2_covid19	30	90	-0.38543	1903.3	0.69996	-0.19660	0.13202
shed2_covid19	30	180	1.03879	1898.2	0.29904	-0.07030	0.22863
shed2_covid19	30	360	-1.57349	1845.3	0.11578	-0.31590	0.03465
shed2_covid19	60	90	-1.11871	1850.6	0.26341	-0.24664	0.06747
shed2_covid19	60	180	0.30332	1918.0	0.76168	-0.11957	0.16332
shed2_covid19	60	360	-2.30380	1762.5	0.02135	-0.36641	-0.02942
shed2_covid19	90	180	1.39047	1852.2	0.16455	-0.04575	0.26867
shed2_covid19	90	360	-1.16788	1894.0	0.24300	-0.29026	0.07359
shed2_covid19	180	360	-2.55618	1764.7	0.01067	-0.38843	-0.05115
shed2_covid19alpha	5	10	0.00000	1911.5	1.00000	-0.20272	0.20272
shed2_covid19alpha	5	30	-0.50966	1895.4	0.61035	-0.26260	0.15427
shed2_covid19alpha	5	60	0.59390	1917.9	0.55265	-0.13670	0.25545
shed2_covid19alpha	5	90	0.73753	1918.0	0.46089	-0.12271	0.27062
shed2_covid19alpha	5	180	1.62944	1895.4	0.10339	-0.03160	0.34202
shed2_covid19alpha	5	360	-0.30889	1906.0	0.75744	-0.23732	0.17273
shed2_covid19alpha	10	30	-0.49620	1913.0	0.61981	-0.26826	0.15993
shed2_covid19alpha	10	60	0.57626	1909.9	0.56451	-0.14270	0.26145
shed2_covid19alpha	10	90	0.71575	1911.4	0.47423	-0.12869	0.27661
shed2_covid19alpha	10	180	1.57636	1866.4	0.11511	-0.03790	0.34831
shed2_covid19alpha	10	360	-0.30047	1917.1	0.76385	-0.24306	0.17848
shed2_covid19alpha	30	60	1.07157	1892.6	0.28405	-0.09427	0.32135
shed2_covid19alpha	30	90	1.20595	1895.1	0.22799	-0.08024	0.33649
shed2_covid19alpha	30	180	2.06251	1832.8	0.03930	0.01028	0.40847
shed2_covid19alpha	30	360	0.19837	1916.3	0.84278	-0.19440	0.23815
shed2_covid19alpha	60	90	0.14592	1917.9	0.88400	-0.18141	0.21058
shed2_covid19alpha	60	180	1.00990	1898.1	0.31267	-0.09027	0.28194
shed2_covid19alpha	60	360	-0.87960	1903.9	0.37919	-0.29605	0.11272
shed2_covid19alpha	90	180	0.85335	1895.7	0.39357	-0.10548	0.26798
shed2_covid19alpha	90	360	-1.01670	1905.8	0.30942	-0.31121	0.09871
shed2_covid19alpha	180	360	-1.88078	1853.4	0.06016	-0.38302	0.00802
shed2_covid19beta	5	10	0.42835	1918.0	0.66845	-0.15656	0.24406
shed2_covid19beta	5	30	-0.13885	1913.0	0.88958	-0.22057	0.19140
shed2_covid19beta	5	60	0.74427	1911.1	0.45681	-0.12093	0.26884
shed2_covid19beta	5	90	0.76460	1916.3	0.44460	-0.12064	0.27480
shed2_covid19beta	5	180	1.97861	1849.8	0.04801	0.00163	0.36921
shed2_covid19beta	5	360	-0.22984	1915.4	0.81824	-0.22840	0.18048
shed2_covid19beta	10	30	-0.55631	1912.3	0.57806	-0.26398	0.14731
shed2_covid19beta	10	60	0.30456	1911.9	0.76073	-0.16432	0.22473
shed2_covid19beta	10	90	0.33123	1916.7	0.74050	-0.16403	0.23070
shed2_covid19beta	10	180	1.51490	1852.1	0.12997	-0.04174	0.32507
shed2_covid19beta	10	360	-0.65063	1914.9	0.51536	-0.27180	0.13639
shed2_covid19beta	30	60	0.86666	1894.7	0.38624	-0.11183	0.28891
shed2_covid19beta	30	90	0.88507	1905.5	0.37623	-0.11146	0.29479
shed2_covid19beta	30	180	2.06893	1812.9	0.03869	0.01041	0.38959
shed2_covid19beta	30	360	-0.08769	1917.6	0.93013	-0.21904	0.20029

blocks	obs.interval.x	obs.interval.y	t	df	p.value	conf.low	conf.high
shed2_covid19beta	60	90	0.03194	1916.3	0.97452	-0.18873	0.19498
shed2_covid19beta	60	180	1.23176	1884.4	0.21819	-0.06601	0.28892
shed2_covid19beta	60	360	-0.96610	1900.3	0.33412	-0.29669	0.10086
shed2_covid19beta	90	180	1.17664	1868.3	0.23949	-0.07224	0.28891
shed2_covid19beta	90	360	-0.98319	1909.5	0.32564	-0.30259	0.10051
shed2_covid19beta	180	360	-2.18532	1823.9	0.02899	-0.39728	-0.02147
shed2_covid19delta	5	10	-0.68497	1914.5	0.49345	-0.42656	0.20573
shed2_covid19delta	5	30	0.92080	1909.5	0.35727	-0.15889	0.44014
shed2_covid19delta	5	60	1.60632	1891.5	0.10837	-0.05293	0.53210
shed2_covid19delta	5	90	1.12553	1909.5	0.26051	-0.12761	0.47136
shed2_covid19delta	5	180	3.68261	1697.0	0.00024	0.23273	0.76311
shed2_covid19delta	5	360	1.19464	1885.0	0.23238	-0.11363	0.46780
shed2_covid19delta	10	30	1.60613	1895.5	0.10841	-0.05550	0.55758
shed2_covid19delta	10	60	2.29033	1870.1	0.02211	0.05029	0.64971
shed2_covid19delta	10	90	1.80622	1895.4	0.07104	-0.02422	0.58881
shed2_covid19delta	10	180	4.36893	1656.4	0.00001	0.33523	0.88144
shed2_covid19delta	10	360	1.89245	1861.7	0.05859	-0.01045	0.58545
shed2_covid19delta	30	60	0.68796	1912.8	0.49156	-0.18315	0.38107
shed2_covid19delta	30	90	0.21183	1918.0	0.83227	-0.25808	0.32058
shed2_covid19delta	30	180	2.76263	1758.2	0.00579	0.10363	0.61095
shed2_covid19delta	30	360	0.25515	1909.6	0.79864	-0.24378	0.31670
shed2_covid19delta	60	90	-0.47076	1912.9	0.63787	-0.34979	0.21437
shed2_covid19delta	60	180	2.06511	1802.3	0.03906	0.01299	0.50368
shed2_covid19delta	60	360	-0.44942	1917.6	0.65318	-0.33524	0.21024
shed2_covid19delta	90	180	2.52134	1758.4	0.01178	0.07242	0.57966
shed2_covid19delta	90	360	0.03645	1909.7	0.97092	-0.27500	0.28542
shed2_covid19delta	180	360	-2.58741	1813.5	0.00975	-0.56403	-0.07764
shed2_diphtheria	5	10	-0.65751	1917.1	0.51093	-0.36509	0.18175
shed2_diphtheria	5	30	0.55003	1912.8	0.58237	-0.18975	0.33767
shed2_diphtheria	5	60	0.82990	1912.3	0.40670	-0.15194	0.37485
shed2_diphtheria	5	90	-0.16420	1917.0	0.86959	-0.29663	0.25079
shed2_diphtheria	5	180	1.23148	1906.2	0.21830	-0.09691	0.42399
shed2_diphtheria	5	360	-1.34208	1896.5	0.17973	-0.48201	0.09034
shed2_diphtheria	10	30	1.21793	1907.7	0.22340	-0.10108	0.43233
shed2_diphtheria	10	60	1.49543	1907.0	0.13497	-0.06327	0.46952
shed2_diphtheria	10	90	0.48747	1918.0	0.62598	-0.20785	0.34535
shed2_diphtheria	10	180	1.89963	1899.1	0.05763	-0.00827	0.51869
shed2_diphtheria	10	360	-0.70705	1904.1	0.47962	-0.39310	0.18477
shed2_diphtheria	30	60	0.28682	1918.0	0.77428	-0.21891	0.29391
shed2_diphtheria	30	90	-0.71158	1907.2	0.47681	-0.36388	0.17013
shed2_diphtheria	30	180	0.69337	1916.6	0.48816	-0.16381	0.34297
shed2_diphtheria	30	360	-1.89131	1871.4	0.05874	-0.54956	0.00997
shed2_diphtheria	60	90	-0.98818	1906.4	0.32319	-0.40107	0.13232
shed2_diphtheria	60	180	0.40364	1916.9	0.68652	-0.20098	0.30514
shed2_diphtheria	60	360	-2.15648	1870.0	0.03117	-0.58676	-0.02782
shed2_diphtheria	90	180	1.38631	1898.3	0.16582	-0.07732	0.45024
shed2_diphtheria	90	360	-1.17259	1904.7	0.24111	-0.46213	0.11629
shed2_diphtheria	180	360	-2.54726	1855.4	0.01094	-0.63607	-0.08268
shed2_fifth	5	10	-0.92727	1907.8	0.35390	-0.17847	0.06388
shed2_fifth	5	30	0.37451	1915.4	0.70807	-0.09268	0.13643
shed2_fifth	5	60	-0.01766	1917.5	0.98592	-0.11675	0.11466
shed2_fifth	5	90	-0.10638	1916.8	0.91529	-0.12148	0.10898
shed2_fifth	5	180	0.75350	1898.6	0.45124	-0.06845	0.15387
shed2_fifth	5	360	-0.03435	1915.2	0.97260	-0.12102	0.11685
shed2_fifth	10	30	1.30301	1895.1	0.19273	-0.03999	0.19832
shed2_fifth	10	60	0.91729	1902.7	0.35911	-0.06402	0.17652
shed2_fifth	10	90	0.83556	1899.8	0.40351	-0.06876	0.17085
shed2_fifth	10	180	1.69218	1862.2	0.09078	-0.01590	0.21590
shed2_fifth	10	360	0.87759	1915.6	0.38028	-0.06817	0.17859
shed2_fifth	30	60	-0.39566	1917.2	0.69240	-0.13651	0.09068
shed2_fifth	30	90	-0.48768	1917.7	0.62583	-0.14123	0.08498
shed2_fifth	30	180	0.37499	1910.0	0.70771	-0.08813	0.12979
shed2_fifth	30	360	-0.40200	1907.3	0.68773	-0.14084	0.09292
shed2_fifth	60	90	-0.08939	1917.9	0.92878	-0.11948	0.10906
shed2_fifth	60	180	0.77882	1904.3	0.43618	-0.06642	0.15392
shed2_fifth	60	360	-0.01731	1912.3	0.98619	-0.11905	0.11697
shed2_fifth	90	180	0.87554	1906.8	0.38139	-0.06071	0.15862
shed2_fifth	90	360	0.06952	1910.4	0.94458	-0.11338	0.12171
shed2_fifth	180	360	-0.77357	1881.8	0.43928	-0.15835	0.06877
shed2_flu	5	10	0.01916	1913.3	0.98471	-0.10557	0.10766
shed2_flu	5	30	-1.10761	1911.7	0.26817	-0.17605	0.04897
shed2_flu	5	60	-0.39317	1918.0	0.69424	-0.13099	0.08724
shed2_flu	5	90	-0.80006	1912.3	0.42377	-0.15818	0.06652
shed2_flu	5	180	-0.01956	1901.3	0.98440	-0.10550	0.10341
shed2_flu	5	360	0.07574	1916.8	0.93964	-0.10373	0.11206
shed2_flu	10	30	-1.15180	1896.4	0.24955	-0.17455	0.04538
shed2_flu	10	60	-0.42204	1913.7	0.67304	-0.12941	0.08358
shed2_flu	10	90	-0.83723	1897.4	0.40257	-0.15668	0.06293
shed2_flu	10	180	-0.04017	1914.3	0.96796	-0.10379	0.09963
shed2_flu	10	360	0.05824	1916.8	0.95357	-0.10211	0.10836
shed2_flu	30	60	0.72705	1911.2	0.46728	-0.07073	0.15406
shed2_flu	30	90	0.30059	1918.0	0.76376	-0.09783	0.13325
shed2_flu	30	180	1.13627	1875.7	0.25599	-0.04538	0.17038
shed2_flu	30	360	1.19407	1905.1	0.23260	-0.04350	0.17892
shed2_flu	60	90	-0.41865	1911.8	0.67552	-0.13619	0.08828
shed2_flu	60	180	0.39163	1902.1	0.69538	-0.08350	0.12516
shed2_flu	60	360	0.47390	1917.0	0.63563	-0.08173	0.13381
shed2_flu	90	180	0.81559	1877.1	0.41484	-0.06292	0.15250
shed2_flu	90	360	0.88305	1905.9	0.37732	-0.06105	0.16105
shed2_flu	180	360	0.09912	1909.0	0.92105	-0.09784	0.10826
shed2_mers	5	10	-1.11262	1897.0	0.26601	-0.11511	0.03178
shed2_mers	5	30	-0.53978	1909.6	0.58941	-0.09170	0.05212
shed2_mers	5	60	1.09651	1907.8	0.27299	-0.02957	0.10457
shed2_mers	5	90	-0.47022	1892.9	0.63825	-0.09157	0.05615
shed2_mers	5	180	0.58154	1904.7	0.56095	-0.04695	0.08654
shed2_mers	5	360	1.08532	1894.2	0.27792	-0.02942	0.10234
shed2_mers	10	30	0.56635	1915.1	0.57122	-0.05388	0.09763
shed2_mers	10	60	2.18144	1859.9	0.02928	0.00799	0.15034
shed2_mers	10	90	0.60548	1917.8	0.54493	-0.05364	0.10156
shed2_mers	10	180	1.70081	1853.2	0.08915	-0.00941	0.13233
shed2_mers	10	360	2.18718	1833.4	0.02886	0.00807	0.14818
shed2_mers	30	60	1.61466	1881.7	0.10655	-0.01230	0.12688
shed2_mers	30	90	0.05365	1913.4	0.95722	-0.07407	0.07823
shed2_mers	30	180	1.12064	1876.2	0.26259	-0.02969	0.10886
shed2_mers	30	360	1.61187	1859.5	0.10716	-0.01219	0.12469
shed2_mers	60	90	-1.51222	1853.5	0.13065	-0.12681	0.01639
shed2_mers	60	180	-0.54064	1917.8	0.58882	-0.08195	0.04653
shed2_mers	60	360	-0.03225	1915.0	0.97427	-0.06438	0.06230
shed2_mers	90	180	1.03156	1846.5	0.30241	-0.03380	0.10880
shed2_mers	90	360	1.50714	1826.1	0.13195	-0.01632	0.12465
shed2_mers	180	360	0.51888	1916.4	0.60390	-0.04633	0.07966
shed2_sars	5	10	0.01341	1916.2	0.98931	-0.15135	0.15343
shed2_sars	5	30	0.94388	1897.2	0.34535	-0.07635	0.21801
shed2_sars	5	60	1.54128	1888.2	0.12342	-0.03122	0.26039
shed2_sars	5	90	0.87376	1916.8	0.38236	-0.08686	0.22644
shed2_sars	5	180	2.42461	1866.4	0.01542	0.03384	0.32032
shed2_sars	5	360	0.87193	1899.7	0.38336	-0.08198	0.21323
shed2_sars	10	30	0.94551	1907.4	0.34452	-0.07497	0.21456
shed2_sars	10	60	1.55324	1900.6	0.12053	-0.02982	0.25691
shed2_sars	10	90	0.87336	1912.2	0.38258	-0.08563	0.22313
shed2_sars	10	180	2.45285	1882.9	0.01426	0.03528	0.31680
shed2_sars	10	360	0.87232	1909.2	0.38314	-0.08062	0.20978
shed2_sars	30	60	0.62262	1917.1	0.53361	-0.09406	0.18156

blocks	obs.interval.x	obs.interval.y	t	df	p.value	conf.low	conf.high
shed2_sars	30	90	-0.01369	1886.5	0.98908	-0.15028	0.14820
shed2_sars	30	180	1.54246	1910.5	0.12313	-0.02884	0.24134
shed2_sars	30	360	-0.07311	1917.9	0.94173	-0.14493	0.13451
shed2_sars	60	90	-0.59402	1875.8	0.55257	-0.19268	0.10309
shed2_sars	60	180	0.91752	1914.7	0.35899	-0.07109	0.19609
shed2_sars	60	360	-0.69442	1916.5	0.48750	-0.18723	0.08931
shed2_sars	90	180	1.44762	1850.9	0.14789	-0.03807	0.25265
shed2_sars	90	360	-0.05460	1889.5	0.95646	-0.15383	0.14550
shed2_sars	180	360	-1.61248	1908.9	0.10702	-0.24702	0.02410
shed7_covid19	5	10	-0.66403	3655.9	0.50671	-0.15551	0.07682
shed7_covid19	5	30	0.41444	3656.8	0.67858	-0.08970	0.13779
shed7_covid19	5	60	0.46809	3650.0	0.63975	-0.08538	0.13893
shed7_covid19	5	90	1.90097	3635.2	0.05738	-0.00336	0.21757
shed7_covid19	5	180	2.25661	3611.1	0.02409	0.01641	0.23386
shed7_covid19	5	360	4.07944	3474.4	0.00005	0.11182	0.31878
shed7_covid19	10	30	1.07908	3651.6	0.28062	-0.05178	0.17856
shed7_covid19	10	60	1.14117	3639.6	0.25387	-0.04748	0.17972
shed7_covid19	10	90	2.56518	3619.5	0.01035	0.03451	0.25838
shed7_covid19	10	180	2.92595	3589.8	0.00346	0.05427	0.27470
shed7_covid19	10	360	4.75296	3438.5	0.00000	0.14960	0.35969
shed7_covid19	30	60	0.04821	3654.9	0.96155	-0.10839	0.11385
shed7_covid19	30	90	1.48834	3644.2	0.13675	-0.02636	0.19248
shed7_covid19	30	180	1.84106	3624.3	0.06570	-0.00657	0.20875
shed7_covid19	30	360	3.66350	3499.4	0.00025	0.08890	0.29361
shed7_covid19	60	90	1.46151	3654.2	0.14396	-0.02743	0.18809
shed7_covid19	60	180	1.81976	3641.4	0.06888	-0.00761	0.20433
shed7_covid19	60	360	3.67480	3537.0	0.00024	0.08794	0.28911
shed7_covid19	90	180	0.33935	3653.5	0.73437	-0.08615	0.12222
shed7_covid19	90	360	2.14932	3573.8	0.03168	0.00950	0.20689
shed7_covid19	180	360	1.82725	3606.9	0.06774	-0.00658	0.18691
shed7_covid19alpha	5	10	-0.64597	3653.5	0.51834	-0.16978	0.08563
shed7_covid19alpha	5	30	0.08435	3655.8	0.93278	-0.12155	0.13248
shed7_covid19alpha	5	60	0.50649	3650.4	0.61254	-0.09099	0.15438
shed7_covid19alpha	5	90	2.59007	3615.2	0.00963	0.03825	0.27651
shed7_covid19alpha	5	180	3.06533	3579.7	0.00219	0.06597	0.30015
shed7_covid19alpha	5	360	5.24496	3409.9	0.00000	0.18649	0.40914
shed7_covid19alpha	10	30	0.72116	3657.6	0.47086	-0.08171	0.17679
shed7_covid19alpha	10	60	1.15709	3634.5	0.24731	-0.05123	0.19877
shed7_covid19alpha	10	90	3.21825	3584.3	0.00130	0.07794	0.32096
shed7_covid19alpha	10	180	3.69352	3540.7	0.00022	0.10563	0.34465
shed7_covid19alpha	10	360	5.85226	3352.2	0.00000	0.22602	0.45376
shed7_covid19alpha	30	60	0.41376	3640.2	0.67907	-0.09806	0.15052
shed7_covid19alpha	30	90	2.46599	3594.4	0.01371	0.03113	0.27269
shed7_covid19alpha	30	180	2.93180	3553.1	0.00339	0.05883	0.29636
shed7_covid19alpha	30	360	5.06842	3370.0	0.00000	0.17926	0.40544
shed7_covid19alpha	60	90	2.12023	3643.2	0.03405	0.00946	0.24190
shed7_covid19alpha	60	180	2.60037	3619.5	0.00935	0.03724	0.26549
shed7_covid19alpha	60	360	4.82184	3478.7	0.00000	0.15791	0.37433
shed7_covid19alpha	90	180	0.45655	3652.3	0.64802	-0.08461	0.13598
shed7_covid19alpha	90	360	2.64353	3560.2	0.00824	0.03628	0.24460
shed7_covid19alpha	180	360	2.20979	3600.0	0.02718	0.01294	0.21657
shed7_covid19beta	5	10	-0.55486	3654.6	0.57902	-0.16103	0.08999
shed7_covid19beta	5	30	0.12867	3656.4	0.89763	-0.11670	0.13310
shed7_covid19beta	5	60	0.51347	3651.3	0.60766	-0.08933	0.15271
shed7_covid19beta	5	90	2.36737	3625.5	0.01797	0.02450	0.26074
shed7_covid19beta	5	180	3.06112	3582.8	0.00222	0.06483	0.29583
shed7_covid19beta	5	360	5.15470	3420.7	0.00000	0.17912	0.39902
shed7_covid19beta	10	30	0.67593	3657.7	0.49913	-0.08309	0.17052
shed7_covid19beta	10	60	1.07151	3638.5	0.28401	-0.05577	0.19020
shed7_covid19beta	10	90	2.90743	3601.9	0.00367	0.05801	0.29827
shed7_covid19beta	10	180	3.59997	3549.8	0.00032	0.09829	0.33340
shed7_covid19beta	10	360	5.67666	3371.6	0.00000	0.21248	0.43670
shed7_covid19beta	30	60	0.37650	3643.2	0.70657	-0.09886	0.14586
shed7_covid19beta	30	90	2.20564	3609.9	0.02747	0.01493	0.25392
shed7_covid19beta	30	180	2.88684	3560.6	0.00391	0.05523	0.28904
shed7_covid19beta	30	360	4.94221	3387.2	0.00000	0.16945	0.39230
shed7_covid19beta	60	90	1.88412	3648.1	0.05963	-0.00450	0.22636
shed7_covid19beta	60	180	2.58459	3619.8	0.00979	0.03588	0.26138
shed7_covid19beta	60	360	4.71343	3484.6	0.00000	0.15032	0.36444
shed7_covid19beta	90	180	0.67431	3648.5	0.50016	-0.07192	0.14733
shed7_covid19beta	90	360	2.76703	3551.7	0.00569	0.04268	0.25022
shed7_covid19beta	180	360	2.11563	3603.3	0.03445	0.00797	0.20952
shed7_covid19delta	5	10	-0.54450	3658.0	0.58613	-0.20113	0.11370
shed7_covid19delta	5	30	0.41022	3657.5	0.68167	-0.12392	0.18949
shed7_covid19delta	5	60	1.49874	3602.5	0.13403	-0.03503	0.26235
shed7_covid19delta	5	90	4.42101	3464.2	0.00001	0.17790	0.46144
shed7_covid19delta	5	180	5.61800	3335.6	0.00000	0.25684	0.53223
shed7_covid19delta	5	360	8.18191	3107.6	0.00000	0.41965	0.68417
shed7_covid19delta	10	30	0.95861	3657.7	0.33782	-0.07997	0.23297
shed7_covid19delta	10	60	2.07865	3605.1	0.03772	0.00894	0.30582
shed7_covid19delta	10	90	5.03481	3468.5	0.00000	0.22188	0.50490
shed7_covid19delta	10	180	6.25264	3340.7	0.00000	0.30083	0.57568
shed7_covid19delta	10	360	8.84862	3113.1	0.00000	0.46365	0.72761
shed7_covid19delta	30	60	1.07362	3612.5	0.28306	-0.06682	0.22857
shed7_covid19delta	30	90	3.99709	3481.5	0.00007	0.14616	0.42761
shed7_covid19delta	30	180	5.19179	3356.0	0.00000	0.22513	0.49836
shed7_covid19delta	30	360	7.76177	3129.7	0.00000	0.38799	0.65026
shed7_covid19delta	60	90	3.06605	3609.6	0.00219	0.07427	0.33775
shed7_covid19delta	60	180	4.32469	3524.9	0.00002	0.15354	0.40821
shed7_covid19delta	60	360	7.07552	3331.4	0.00000	0.31681	0.55969
shed7_covid19delta	90	180	1.23156	3634.6	0.21819	-0.04432	0.19404
shed7_covid19delta	90	360	4.03455	3510.9	0.00006	0.11938	0.34510
shed7_covid19delta	180	360	2.86522	3600.4	0.00419	0.04969	0.26507
shed7_diphtheria	5	10	-1.81405	3630.0	0.06975	-0.28767	0.01117
shed7_diphtheria	5	30	-1.30512	3641.5	0.19193	-0.24612	0.04940
shed7_diphtheria	5	60	-0.33288	3657.1	0.73924	-0.16566	0.11757
shed7_diphtheria	5	90	0.50954	3645.9	0.61041	-0.10271	0.17484
shed7_diphtheria	5	180	1.02851	3626.2	0.30378	-0.06487	0.20804
shed7_diphtheria	5	360	3.06613	3572.0	0.00218	0.07487	0.34043
shed7_diphtheria	10	30	0.50702	3656.4	0.61217	-0.11436	0.19415
shed7_diphtheria	10	60	1.50912	3619.4	0.13136	-0.03417	0.26258
shed7_diphtheria	10	90	2.34622	3583.1	0.01902	0.02865	0.31999
shed7_diphtheria	10	180	2.86766	3543.1	0.00416	0.06637	0.35330
shed7_diphtheria	10	360	4.84518	3459.2	0.00000	0.20593	0.48587
shed7_diphtheria	30	60	0.99319	3633.2	0.32068	-0.07239	0.22102
shed7_diphtheria	30	90	1.83071	3602.4	0.06723	-0.00954	0.27839
shed7_diphtheria	30	180	2.35084	3566.8	0.01879	0.02821	0.31168
shed7_diphtheria	30	360	4.34140	3489.0	0.00001	0.16781	0.44421
shed7_diphtheria	60	90	0.85618	3651.5	0.39196	-0.07754	0.19776
shed7_diphtheria	60	180	1.38559	3635.7	0.16596	-0.03969	0.23094
shed7_diphtheria	60	360	3.45175	3587.7	0.00056	0.10009	0.36330
shed7_diphtheria	90	180	0.52622	3653.2	0.59877	-0.09682	0.16786
shed7_diphtheria	90	360	2.61713	3622.8	0.00890	0.04304	0.30013
shed7_diphtheria	180	360	2.11658	3643.8	0.03436	0.01003	0.26211
shed7_fifth	5	10	-0.36530	3656.5	0.71491	-0.11482	0.07875
shed7_fifth	5	30	0.51038	3655.0	0.60982	-0.06987	0.11905
shed7_fifth	5	60	0.59984	3643.0	0.54865	-0.06446	0.12129
shed7_fifth	5	90	1.59651	3631.1	0.11046	-0.01707	0.16680
shed7_fifth	5	180	1.16140	3621.4	0.24556	-0.03723	0.14542
shed7_fifth	5	360	4.35632	3374.3	0.00001	0.10307	0.27179
shed7_fifth	10	30	0.87548	3649.3	0.38137	-0.05283	0.13808
shed7_fifth	10	60	0.97000	3632.3	0.33211	-0.04744	0.14033
shed7_fifth	10	90	1.95940	3617.4	0.05014	-0.00006	0.18585

blocks	obs.interval.x	obs.interval.y	t	df	p.value	conf.low	conf.high
shed7_fifth	10	180	1.53136	3605.7	0.12577	-0.02022	0.16448
shed7_fifth	10	360	4.71357	3340.6	0.00000	0.12000	0.29093
shed7_fifth	30	60	0.08197	3653.4	0.93467	-0.08766	0.09531
shed7_fifth	30	90	1.08873	3646.0	0.27634	-0.04026	0.14081
shed7_fifth	30	180	0.64345	3639.1	0.51998	-0.06040	0.11942
shed7_fifth	30	360	3.85480	3420.6	0.00012	0.08002	0.24567
shed7_fifth	60	90	1.02463	3656.2	0.30561	-0.04243	0.13533
shed7_fifth	60	180	0.57062	3653.1	0.56829	-0.06256	0.11393
shed7_fifth	60	360	3.84849	3474.0	0.00012	0.07800	0.24003
shed7_fifth	90	180	-0.46658	3657.2	0.64083	-0.10802	0.06649
shed7_fifth	90	360	2.76115	3504.6	0.00579	0.03264	0.19250
shed7_fifth	180	360	3.29952	3523.9	0.00098	0.05410	0.21256
shed7_flu	5	10	-0.33063	3652.0	0.74094	-0.11739	0.08351
shed7_flu	5	30	0.04358	3658.0	0.96524	-0.09614	0.10051
shed7_flu	5	60	0.34373	3653.0	0.73107	-0.07968	0.11356
shed7_flu	5	90	-0.02193	3657.2	0.98250	-0.09879	0.09661
shed7_flu	5	180	1.40462	3649.5	0.16022	-0.02725	0.16496
shed7_flu	5	360	3.58697	3520.2	0.00034	0.07458	0.25439
shed7_flu	10	30	0.37354	3651.6	0.70877	-0.08126	0.11951
shed7_flu	10	60	0.67289	3636.2	0.50106	-0.06484	0.13260
shed7_flu	10	90	0.31142	3647.0	0.75550	-0.08392	0.11562
shed7_flu	10	180	1.71270	3629.5	0.08685	-0.01242	0.18400
shed7_flu	10	360	3.85996	3464.3	0.00012	0.08927	0.27357
shed7_flu	30	60	0.29960	3653.4	0.76450	-0.08180	0.11131
shed7_flu	30	90	-0.06584	3657.4	0.94751	-0.10091	0.09435
shed7_flu	30	180	1.36103	3650.0	0.17359	-0.02937	0.16270
shed7_flu	30	360	3.54227	3522.0	0.00040	0.07247	0.25213
shed7_flu	60	90	-0.36862	3656.1	0.71243	-0.11395	0.07788
shed7_flu	60	180	1.07944	3657.5	0.28046	-0.04238	0.14620
shed7_flu	60	360	3.28867	3564.5	0.00102	0.05958	0.23550
shed7_flu	90	180	1.43761	3653.8	0.15063	-0.02545	0.16534
shed7_flu	90	360	3.64176	3538.2	0.00027	0.07643	0.25471
shed7_flu	180	360	2.14541	3576.4	0.03199	0.00824	0.18302
shed7_mers	5	10	-0.04698	3658.0	0.96253	-0.07005	0.06677
shed7_mers	5	30	-0.20421	3657.9	0.83820	-0.07531	0.06110
shed7_mers	5	60	-0.11171	3652.7	0.91106	-0.07096	0.06331
shed7_mers	5	90	-0.49673	3656.9	0.61941	-0.08651	0.05153
shed7_mers	5	180	0.96701	3645.3	0.33360	-0.03369	0.09926
shed7_mers	5	360	2.53862	3517.9	0.01117	0.01841	0.14334
shed7_mers	10	30	-0.15708	3657.9	0.87519	-0.07367	0.06274
shed7_mers	10	60	-0.06383	3652.7	0.94911	-0.06932	0.06495
shed7_mers	10	90	-0.45017	3656.9	0.65262	-0.08487	0.05317
shed7_mers	10	180	1.01537	3645.3	0.31000	-0.03205	0.10090
shed7_mers	10	360	2.59010	3517.9	0.00963	0.02005	0.14497
shed7_mers	30	60	0.09605	3654.2	0.92349	-0.06365	0.07021
shed7_mers	30	90	-0.29580	3656.0	0.76740	-0.07920	0.05843
shed7_mers	30	180	1.18026	3647.7	0.23797	-0.02637	0.10616
shed7_mers	30	360	2.77153	3525.6	0.00561	0.02574	0.15022
shed7_mers	60	90	-0.39529	3646.6	0.69265	-0.08142	0.05410
shed7_mers	60	180	1.10153	3656.4	0.27074	-0.02855	0.10178
shed7_mers	60	360	2.71947	3563.8	0.00657	0.02363	0.14576
shed7_mers	90	180	1.46889	3636.7	0.14195	-0.01683	0.11738
shed7_mers	90	360	3.05487	3494.4	0.00227	0.03523	0.16149
shed7_mers	180	360	1.56259	3585.5	0.11824	-0.01225	0.10842
shed7_sars	5	10	-0.28446	3657.1	0.77607	-0.13369	0.09981
shed7_sars	5	30	0.45493	3648.8	0.64919	-0.08681	0.13927
shed7_sars	5	60	0.17857	3654.1	0.85829	-0.10361	0.12438
shed7_sars	5	90	1.21720	3627.8	0.22361	-0.04205	0.17976
shed7_sars	5	180	1.46757	3628.6	0.14231	-0.02790	0.19402
shed7_sars	5	360	3.88299	3506.1	0.00011	0.10334	0.31414
shed7_sars	10	30	0.74265	3642.3	0.45774	-0.07080	0.15714
shed7_sars	10	60	0.46616	3649.5	0.64113	-0.08759	0.14224
shed7_sars	10	90	1.50385	3617.0	0.13271	-0.02606	0.19764
shed7_sars	10	180	1.75196	3617.9	0.07986	-0.01191	0.21191
shed7_sars	10	360	4.15888	3484.9	0.00003	0.11929	0.33208
shed7_sars	30	60	-0.27955	3656.9	0.77984	-0.12699	0.09529
shed7_sars	30	90	0.77400	3651.8	0.43898	-0.06534	0.15059
shed7_sars	30	180	1.03141	3652.2	0.30242	-0.05120	0.16486
shed7_sars	30	360	3.49779	3567.3	0.00047	0.08021	0.28482
shed7_sars	60	90	1.05204	3645.5	0.29285	-0.05050	0.16744
shed7_sars	60	180	1.30694	3646.0	0.19132	-0.03635	0.18171
shed7_sars	60	360	3.76272	3547.4	0.00017	0.09500	0.30172
shed7_sars	90	180	0.26331	3658.0	0.79233	-0.09158	0.12000
shed7_sars	90	360	2.74441	3606.7	0.00609	0.03995	0.23983
shed7_sars	180	360	2.46403	3605.7	0.01378	0.02568	0.22569
shed8_covid19	5	10	-0.58006	4433.6	0.56190	-0.30580	0.16616
shed8_covid19	5	30	-1.65005	4413.6	0.09900	-0.44453	0.03822
shed8_covid19	5	60	1.83698	4403.4	0.06628	-0.01402	0.43114
shed8_covid19	5	90	2.46670	4345.6	0.01367	0.05602	0.48993
shed8_covid19	5	180	6.52838	4044.9	0.00000	0.47245	0.87800
shed8_covid19	5	360	10.57287	3484.8	0.00000	0.82667	1.20306
shed8_covid19	10	30	-1.06705	4429.8	0.28601	-0.37831	0.11164
shed8_covid19	10	60	2.40975	4375.3	0.01600	0.05190	0.50486
shed8_covid19	10	90	3.04158	4304.0	0.00237	0.12184	0.56375
shed8_covid19	10	180	7.05480	3978.5	0.00000	0.53799	0.95210
shed8_covid19	10	360	11.03087	3420.3	0.00000	0.89189	1.27748
shed8_covid19	30	60	3.47764	4324.7	0.00051	0.17961	0.64381
shed8_covid19	30	90	4.11736	4237.1	0.00004	0.24941	0.70284
shed8_covid19	30	180	8.07807	3885.0	0.00000	0.66519	1.09156
shed8_covid19	30	360	11.97849	3335.4	0.00000	1.01865	1.41739
shed8_covid19	60	90	0.61129	4423.1	0.54104	-0.14217	0.27100
shed8_covid19	60	180	4.77410	4216.1	0.00000	0.27503	0.65831
shed8_covid19	60	360	8.97503	3675.5	0.00000	0.63017	0.98245
shed8_covid19	90	180	4.26111	4307.9	0.00002	0.21718	0.58733
shed8_covid19	90	360	8.60832	3803.9	0.00000	0.57292	0.91086
shed8_covid19	180	360	4.42969	4172.2	0.00001	0.18932	0.48996
shed8_covid19alpha	5	10	-0.21516	4437.6	0.82965	-0.30518	0.24482
shed8_covid19alpha	5	30	-1.33257	4421.8	0.18274	-0.47421	0.09042
shed8_covid19alpha	5	60	2.98014	4343.6	0.00290	0.13285	0.64373
shed8_covid19alpha	5	90	2.46784	4352.1	0.01363	0.06630	0.57874
shed8_covid19alpha	5	180	8.23787	3865.6	0.00000	0.74450	1.20956
shed8_covid19alpha	5	360	12.91576	3261.1	0.00000	1.20849	1.64106
shed8_covid19alpha	10	30	-1.11771	4426.6	0.26375	-0.44536	0.12194
shed8_covid19alpha	10	60	3.19334	4331.0	0.00142	0.16156	0.67538
shed8_covid19alpha	10	90	2.68338	4340.0	0.00732	0.09501	0.61039
shed8_covid19alpha	10	180	8.43364	3843.5	0.00000	0.77306	1.24135
shed8_covid19alpha	10	360	13.08414	3242.9	0.00000	1.23693	1.67298
shed8_covid19alpha	30	60	4.29668	4256.9	0.00002	0.31545	0.84491
shed8_covid19alpha	30	90	3.79882	4268.3	0.00015	0.24893	0.77990
shed8_covid19alpha	30	180	9.44290	3731.1	0.00000	0.92622	1.41162
shed8_covid19alpha	30	360	13.95234	3153.8	0.00000	1.38948	1.84386
shed8_covid19alpha	60	90	-0.54472	4437.8	0.58598	-0.30247	0.17093
shed8_covid19alpha	60	180	5.47499	4173.7	0.00000	0.37792	0.79956
shed8_covid19alpha	60	360	10.54257	3558.0	0.00000	0.84373	1.22924
shed8_covid19alpha	90	180	6.05941	4160.4	0.00000	0.44274	0.86627
shed8_covid19alpha	90	360	11.15171	3542.8	0.00000	0.90846	1.29604
shed8_covid19alpha	180	360	5.44735	4113.5	0.00000	0.28660	0.60890
shed8_covid19beta	5	10	-0.31911	4436.8	0.74966	-0.31535	0.22707
shed8_covid19beta	5	30	-1.29073	4423.3	0.19686	-0.45953	0.09467
shed8_covid19beta	5	60	3.02222	4345.5	0.00252	0.13609	0.63869
shed8_covid19beta	5	90	2.45177	4354.8	0.01425	0.06318	0.56745
shed8_covid19beta	5	180	8.14773	3875.7	0.00000	0.72243	1.18027
shed8_covid19beta	5	360	12.77870	3271.4	0.00000	1.17451	1.60026

blocks	obs.interval.x	obs.interval.y	t	df	p.value	conf.low	conf.high
shed8_covid19beta	10	30	-0.97089	4430.4	0.33166	-0.41753	0.14095
shed8_covid19beta	10	60	3.33525	4324.9	0.00086	0.17787	0.68519
shed8_covid19beta	10	90	2.76914	4335.0	0.00564	0.10497	0.61395
shed8_covid19beta	10	180	8.43035	3839.8	0.00000	0.76398	1.22701
shed8_covid19beta	10	360	13.01501	3241.6	0.00000	1.21587	1.64719
shed8_covid19beta	30	60	4.29756	4264.4	0.00002	0.30987	0.82977
shed8_covid19beta	30	90	3.74233	4276.6	0.00018	0.23699	0.75851
shed8_covid19beta	30	180	9.32471	3747.9	0.00000	0.89540	1.37217
shed8_covid19beta	30	360	13.80122	3168.5	0.00000	1.34680	1.79284
shed8_covid19beta	60	90	-0.60603	4437.7	0.54453	-0.30523	0.16108
shed8_covid19beta	60	180	5.32020	4179.2	0.00000	0.35614	0.77179
shed8_covid19beta	60	360	10.31922	3566.4	0.00000	0.81000	1.19000
shed8_covid19beta	90	180	5.97099	4164.8	0.00000	0.42720	0.84487
shed8_covid19beta	90	360	10.99882	3549.8	0.00000	0.88097	1.26318
shed8_covid19beta	180	360	5.36953	4115.5	0.00000	0.27683	0.59524
shed8_covid19delta	5	10	0.25814	4437.8	0.79631	-0.30597	0.39877
shed8_covid19delta	5	30	-1.64522	4408.2	0.10000	-0.67427	0.05896
shed8_covid19delta	5	60	5.92801	4184.6	0.00000	0.63672	1.26599
shed8_covid19delta	5	90	4.81654	4224.8	0.00000	0.46235	1.09711
shed8_covid19delta	5	180	12.58901	3512.6	0.00000	1.54781	2.11886
shed8_covid19delta	5	360	18.03689	2897.2	0.00000	2.19009	2.72433
shed8_covid19delta	10	30	-1.88764	4412.7	0.05914	-0.72177	0.01367
shed8_covid19delta	10	60	5.61597	4172.5	0.00000	0.58904	1.22087
shed8_covid19delta	10	90	4.51183	4213.5	0.00001	0.41468	1.05199
shed8_covid19delta	10	180	12.20987	3498.9	0.00000	1.49999	2.07388
shed8_covid19delta	10	360	17.59663	2888.9	0.00000	2.14218	2.67945
shed8_covid19delta	30	60	7.44065	4021.5	0.00000	0.92727	1.59075
shed8_covid19delta	30	90	6.37626	4068.7	0.00000	0.75304	1.42173
shed8_covid19delta	30	180	13.79597	3345.7	0.00000	1.83671	2.44527
shed8_covid19delta	30	360	18.88476	2798.1	0.00000	2.47779	3.05194
shed8_covid19delta	60	90	-1.21756	4435.7	0.22346	-0.44797	0.10472
shed8_covid19delta	60	180	7.23288	4058.5	0.00000	0.64291	1.12105
shed8_covid19delta	60	360	13.62177	3295.4	0.00000	1.28911	1.72261
shed8_covid19delta	90	180	8.51193	4011.0	0.00000	0.81093	1.29628
shed8_covid19delta	90	360	14.90125	3253.2	0.00000	1.45676	1.89820
shed8_covid19delta	180	360	7.12189	3965.5	0.00000	0.45213	0.79562
shed8_diphtheria	5	10	0.41767	4437.8	0.67621	-0.22796	0.35138
shed8_diphtheria	5	30	-2.72023	4360.8	0.00655	-0.74022	-0.12014
shed8_diphtheria	5	60	-1.34679	4422.2	0.17812	-0.50220	0.09319
shed8_diphtheria	5	90	0.73783	4434.8	0.46066	-0.17765	0.39207
shed8_diphtheria	5	180	2.73496	4361.2	0.00626	0.10714	0.64961
shed8_diphtheria	5	360	7.27041	3955.6	0.00000	0.67309	1.17015
shed8_diphtheria	10	30	-3.10115	4368.3	0.00194	-0.80286	-0.18092
shed8_diphtheria	10	60	-1.74751	4425.6	0.08062	-0.56488	0.03245
shed8_diphtheria	10	90	0.31200	4432.9	0.75505	-0.24038	0.33137
shed8_diphtheria	10	180	2.27997	4353.3	0.02266	0.04437	0.58896
shed8_diphtheria	10	360	6.75207	3940.7	0.00000	0.61022	1.10960
shed8_diphtheria	30	60	1.38932	4413.9	0.16481	-0.09278	0.54413
shed8_diphtheria	30	90	3.43742	4328.0	0.00059	0.23089	0.84388
shed8_diphtheria	30	180	5.39415	4154.6	0.00000	0.51468	1.10243
shed8_diphtheria	30	360	9.70605	3660.5	0.00000	1.07874	1.62486
shed8_diphtheria	60	90	2.07858	4405.1	0.03771	0.01771	0.60572
shed8_diphtheria	60	180	4.06933	4281.9	0.00005	0.30206	0.86370
shed8_diphtheria	60	360	8.52593	3824.4	0.00000	0.86717	1.38509
shed8_diphtheria	90	180	1.98981	4388.5	0.04667	0.00399	0.53835
shed8_diphtheria	90	360	6.54135	4013.2	0.00000	0.57032	1.05851
shed8_diphtheria	180	360	4.67048	4220.5	0.00000	0.31521	0.77128
shed8_fifth	5	10	1.06465	4423.3	0.28709	-0.07391	0.24959
shed8_fifth	5	30	0.12217	4438.0	0.90277	-0.15589	0.17661
shed8_fifth	5	60	1.64895	4405.2	0.09923	-0.02536	0.29383
shed8_fifth	5	90	2.42166	4330.7	0.01549	0.03637	0.34561
shed8_fifth	5	180	4.90089	4166.8	0.00000	0.22269	0.51965
shed8_fifth	5	360	6.96210	3955.6	0.00000	0.36535	0.65177
shed8_fifth	10	30	-0.93962	4423.9	0.34746	-0.23913	0.08418
shed8_fifth	10	60	0.58759	4434.3	0.55683	-0.10840	0.20120
shed8_fifth	10	90	1.35122	4393.5	0.17669	-0.04651	0.25282
shed8_fifth	10	180	3.87596	4266.6	0.00011	0.14002	0.42665
shed8_fifth	10	360	5.98373	4077.0	0.00000	0.28287	0.55857
shed8_fifth	30	60	1.52260	4406.0	0.12793	-0.03563	0.28337
shed8_fifth	30	90	2.29176	4332.1	0.02197	0.02611	0.33515
shed8_fifth	30	180	4.76738	4168.9	0.00000	0.21243	0.50919
shed8_fifth	30	360	6.82533	3958.0	0.00000	0.35509	0.64130
shed8_fifth	60	90	0.75522	4415.1	0.45015	-0.09058	0.20409
shed8_fifth	60	180	3.29729	4309.7	0.00098	0.09606	0.37782
shed8_fifth	60	360	5.42353	4134.3	0.00000	0.23901	0.50964
shed8_fifth	90	180	2.61245	4392.5	0.00902	0.04496	0.31540
shed8_fifth	90	360	4.81119	4262.4	0.00000	0.18816	0.44697
shed8_fifth	180	360	2.20774	4391.1	0.02731	0.01539	0.25939
shed8_flu	5	10	-0.16252	4437.6	0.87090	-0.17065	0.14452
shed8_flu	5	30	-1.39878	4404.3	0.16195	-0.28127	0.04703
shed8_flu	5	60	-0.51253	4430.0	0.60830	-0.20213	0.11835
shed8_flu	5	90	0.88142	4433.2	0.37814	-0.08493	0.22366
shed8_flu	5	180	2.64952	4313.8	0.00809	0.05096	0.34094
shed8_flu	5	360	6.32338	3916.1	0.00000	0.29867	0.56710
shed8_flu	10	30	-1.23717	4411.4	0.21609	-0.26895	0.06084
shed8_flu	10	60	-0.35105	4433.3	0.72557	-0.18983	0.13217
shed8_flu	10	90	1.04207	4429.9	0.29744	-0.07265	0.23752
shed8_flu	10	180	2.80988	4299.9	0.00498	0.06318	0.35484
shed8_flu	10	360	6.47050	3894.6	0.00000	0.31082	0.58107
shed8_flu	30	60	0.88082	4428.9	0.37846	-0.09221	0.24266
shed8_flu	30	90	2.26029	4375.1	0.02385	0.02473	0.34824
shed8_flu	30	180	4.01410	4170.3	0.00006	0.16016	0.46597
shed8_flu	30	360	7.55511	3722.2	0.00000	0.40727	0.69273
shed8_flu	60	90	1.38243	4413.1	0.16691	-0.04652	0.26905
shed8_flu	60	180	3.13576	4249.6	0.00173	0.08914	0.38654
shed8_flu	60	360	6.73469	3822.5	0.00000	0.33656	0.61299
shed8_flu	90	180	1.74424	4355.5	0.08119	-0.01569	0.26885
shed8_flu	90	360	5.42909	3987.1	0.00000	0.23224	0.49479
shed8_flu	180	360	3.86475	4253.4	0.00011	0.11674	0.35713
shed8_mers	5	10	-0.32185	4435.7	0.74758	-0.08944	0.06422
shed8_mers	5	30	-1.95880	4360.1	0.05020	-0.16313	0.00007
shed8_mers	5	60	-1.34387	4378.1	0.17906	-0.13623	0.02542
shed8_mers	5	90	-0.72980	4417.9	0.46555	-0.10793	0.04937
shed8_mers	5	180	-1.61725	4418.2	0.10589	-0.14350	0.01377
shed8_mers	5	360	-0.70354	4411.5	0.48176	-0.10746	0.05070
shed8_mers	10	30	-1.63941	4383.6	0.10120	-0.15134	0.01350
shed8_mers	10	60	-1.02750	4398.6	0.30424	-0.12444	0.03886
shed8_mers	10	90	-0.41101	4429.1	0.68108	-0.09617	0.06283
shed8_mers	10	180	-1.28893	4429.3	0.19749	-0.13173	0.02722
shed8_mers	10	360	-0.38674	4424.6	0.69896	-0.09569	0.06415
shed8_mers	30	60	0.59449	4436.7	0.55221	-0.06003	0.11228
shed8_mers	30	90	1.21776	4418.3	0.22338	-0.03187	0.13637
shed8_mers	30	180	0.38852	4418.0	0.69765	-0.06743	0.10077
shed8_mers	30	360	1.23291	4423.8	0.21767	-0.03137	0.13767
shed8_mers	60	90	0.61437	4427.0	0.53901	-0.05724	0.10950
shed8_mers	60	180	-0.22250	4426.8	0.82394	-0.09281	0.07389
shed8_mers	60	360	0.63250	4431.0	0.52709	-0.05675	0.11080
shed8_mers	90	180	-0.85872	4438.0	0.39054	-0.11683	0.04566
shed8_mers	90	360	0.02162	4437.5	0.98275	-0.08078	0.08258
shed8_mers	180	360	0.87601	4437.5	0.38107	-0.04517	0.11814
shed8_sars	5	10	-0.45917	4433.3	0.64613	-0.24449	0.15170
shed8_sars	5	30	-3.23677	4294.5	0.00122	-0.57140	-0.14031
shed8_sars	5	60	-1.17378	4422.5	0.24055	-0.32115	0.08061

blocks	obs.interval.x	obs.interval.y	t	df	p.value	conf.low	conf.high
shed8_sars	5	90	0.53002	4432.9	0.59613	-0.13981	0.24341
shed8_sars	5	180	1.99363	4382.3	0.04625	0.00312	0.37255
shed8_sars	5	360	5.89066	4077.0	0.00000	0.34291	0.68502
shed8_sars	10	30	-2.77687	4338.9	0.00551	-0.52794	-0.09098
shed8_sars	10	60	-0.70983	4434.8	0.47785	-0.27791	0.13016
shed8_sars	10	90	0.98770	4418.4	0.32335	-0.09672	0.29311
shed8_sars	10	180	2.44082	4347.0	0.01469	0.04609	0.42238
shed8_sars	10	360	6.28675	4009.5	0.00000	0.38561	0.73511
shed8_sars	30	60	2.08980	4369.7	0.03669	0.01458	0.45660
shed8_sars	30	90	3.75889	4241.0	0.00017	0.19504	0.62028
shed8_sars	30	180	5.16363	4094.7	0.00000	0.33726	0.75013
shed8_sars	30	360	8.77664	3675.3	0.00000	0.67551	1.06413
shed8_sars	60	90	1.70599	4399.9	0.08808	-0.02567	0.36982
shed8_sars	60	180	3.16140	4312.3	0.00158	0.11704	0.49918
shed8_sars	60	360	6.98956	3952.3	0.00000	0.45633	0.81214
shed8_sars	90	180	1.47104	4410.5	0.14135	-0.04526	0.31734
shed8_sars	90	360	5.41394	4144.4	0.00000	0.29480	0.62952
shed8_sars	180	360	4.01066	4281.4	0.00006	0.16671	0.48555
shed9_covid19	5	10	1.78041	4488.8	0.07508	-0.00506	0.10506
shed9_covid19	5	30	0.30631	4624.7	0.75938	-0.04846	0.06641
shed9_covid19	5	60	1.58945	4116.9	0.11204	-0.00978	0.09354
shed9_covid19	5	90	8.29750	2758.2	0.00000	0.14425	0.23353
shed9_covid19	5	180	10.78092	2579.8	0.00000	0.19719	0.28486
shed9_covid19	5	360	10.63522	2590.7	0.00000	0.19415	0.28192
shed9_covid19	10	30	-1.55451	4631.6	0.12013	-0.09277	0.01071
shed9_covid19	10	60	-0.35191	4535.4	0.72492	-0.05335	0.03711
shed9_covid19	10	90	7.35780	2968.1	0.00000	0.10188	0.17590
shed9_covid19	10	180	10.39172	2702.9	0.00000	0.15498	0.22707
shed9_covid19	10	360	10.21210	2719.3	0.00000	0.15193	0.22414
shed9_covid19	30	60	1.34105	4354.4	0.17997	-0.01520	0.08101
shed9_covid19	30	90	8.71638	2856.7	0.00000	0.13944	0.22039
shed9_covid19	30	180	11.49324	2637.3	0.00000	0.19246	0.27164
shed9_covid19	30	360	11.32954	2650.8	0.00000	0.18942	0.26870
shed9_covid19	60	90	9.08381	3222.3	0.00000	0.11528	0.17874
shed9_covid19	60	180	12.76193	2856.6	0.00000	0.16855	0.22974
shed9_covid19	60	360	12.54145	2879.5	0.00000	0.16549	0.22682
shed9_covid19	90	180	6.34756	4354.0	0.00000	0.03603	0.06824
shed9_covid19	90	360	5.93435	4398.6	0.00000	0.03291	0.06538
shed9_covid19	180	360	-0.42230	4675.7	0.67283	-0.01688	0.01090
shed9_covid19alpha	5	10	1.30874	4655.7	0.19069	-0.02341	0.11743
shed9_covid19alpha	5	30	-0.44537	4659.2	0.65607	-0.09234	0.05815
shed9_covid19alpha	5	60	2.10045	4287.6	0.03575	0.00456	0.13220
shed9_covid19alpha	5	90	9.57726	2651.5	0.00000	0.20663	0.31303
shed9_covid19alpha	5	180	12.00454	2508.4	0.00000	0.26852	0.37336
shed9_covid19alpha	5	360	11.93527	2512.7	0.00000	0.26678	0.37168
shed9_covid19alpha	10	30	-1.72319	4597.6	0.08492	-0.13703	0.00883
shed9_covid19alpha	10	60	0.68593	4428.2	0.49279	-0.03970	0.08244
shed9_covid19alpha	10	90	8.36870	2697.4	0.00000	0.16296	0.26269
shed9_covid19alpha	10	180	10.95340	2533.5	0.00000	0.22489	0.32297
shed9_covid19alpha	10	360	10.87950	2538.5	0.00000	0.22316	0.32129
shed9_covid19alpha	30	60	2.51669	4145.3	0.01188	0.01889	0.15205
shed9_covid19alpha	30	90	9.61394	2614.4	0.00000	0.22044	0.33340
shed9_covid19alpha	30	180	11.88892	2488.2	0.00000	0.28228	0.39379
shed9_covid19alpha	30	360	11.82413	2492.0	0.00000	0.28055	0.39210
shed9_covid19alpha	60	90	9.38406	2915.2	0.00000	0.15145	0.23146
shed9_covid19alpha	60	180	12.70838	2653.8	0.00000	0.21359	0.29153
shed9_covid19alpha	60	360	12.61218	2661.8	0.00000	0.21185	0.28986
shed9_covid19alpha	90	180	7.23770	4295.6	0.00000	0.04456	0.07766
shed9_covid19alpha	90	360	7.00396	4322.4	0.00000	0.04277	0.07603
shed9_covid19alpha	180	360	-0.24017	4677.3	0.81021	-0.01566	0.01224
shed9_covid19beta	5	10	1.58054	4607.2	0.11405	-0.01325	0.12351
shed9_covid19beta	5	30	-0.25067	4674.3	0.80208	-0.08293	0.06413
shed9_covid19beta	5	60	2.06551	4219.6	0.03894	0.00337	0.12911
shed9_covid19beta	5	90	9.33477	2651.4	0.00000	0.19917	0.30510
shed9_covid19beta	5	180	11.71852	2509.9	0.00000	0.25976	0.36417
shed9_covid19beta	5	360	11.64907	2514.2	0.00000	0.25803	0.36248
shed9_covid19beta	10	30	-1.82088	4573.1	0.06869	-0.13401	0.00495
shed9_covid19beta	10	60	0.37507	4472.5	0.70763	-0.04697	0.06919
shed9_covid19beta	10	90	8.18925	2738.7	0.00000	0.14984	0.24418
shed9_covid19beta	10	180	10.87397	2558.1	0.00000	0.21052	0.30315
shed9_covid19beta	10	360	10.79542	2563.7	0.00000	0.20879	0.30147
shed9_covid19beta	30	60	2.31482	4156.3	0.02067	0.01158	0.13971
shed9_covid19beta	30	90	9.43189	2634.6	0.00000	0.20717	0.31591
shed9_covid19beta	30	180	11.74997	2500.6	0.00000	0.26774	0.37500
shed9_covid19beta	30	360	11.68248	2504.7	0.00000	0.26600	0.37331
shed9_covid19beta	60	90	9.40597	2950.6	0.00000	0.14715	0.22465
shed9_covid19beta	60	180	12.77904	2676.6	0.00000	0.20802	0.28343
shed9_covid19beta	60	360	12.67920	2685.1	0.00000	0.20628	0.28175
shed9_covid19beta	90	180	7.10650	4305.2	0.00000	0.04332	0.07633
shed9_covid19beta	90	360	6.87258	4331.8	0.00000	0.04154	0.07470
shed9_covid19beta	180	360	-0.24017	4677.3	0.81021	-0.01566	0.01224
shed9_covid19delta	5	10	2.09247	4417.5	0.03645	0.00825	0.25329
shed9_covid19delta	5	30	-0.07435	4676.1	0.94073	-0.14035	0.13009
shed9_covid19delta	5	60	4.50943	3723.3	0.00001	0.14469	0.36728
shed9_covid19delta	5	90	11.22479	2433.4	0.00000	0.46097	0.65612
shed9_covid19delta	5	180	12.65914	2388.1	0.00000	0.52981	0.72404
shed9_covid19delta	5	360	12.65010	2388.4	0.00000	0.52938	0.72361
shed9_covid19delta	10	30	-2.20203	4455.3	0.02771	-0.25689	-0.01491
shed9_covid19delta	10	60	2.62583	4290.6	0.00867	0.03173	0.21870
shed9_covid19delta	10	90	10.94503	2493.9	0.00000	0.35114	0.50442
shed9_covid19delta	10	180	12.79332	2419.6	0.00000	0.42010	0.57220
shed9_covid19delta	10	360	12.78162	2420.1	0.00000	0.41967	0.57178
shed9_covid19delta	30	60	4.67057	3769.1	0.00000	0.15150	0.37072
shed9_covid19delta	30	90	11.55635	2437.3	0.00000	0.46803	0.65932
shed9_covid19delta	30	180	13.02269	2390.2	0.00000	0.53688	0.72723
shed9_covid19delta	30	360	13.01344	2390.5	0.00000	0.53645	0.72680
shed9_covid19delta	60	90	10.41270	2626.1	0.00000	0.24559	0.35954
shed9_covid19delta	60	180	12.94740	2488.8	0.00000	0.31476	0.42712
shed9_covid19delta	60	360	12.93123	2489.7	0.00000	0.31433	0.42670
shed9_covid19delta	90	180	7.92232	4254.2	0.00000	0.05146	0.08530
shed9_covid19delta	90	360	7.86440	4261.0	0.00000	0.05101	0.08489
shed9_covid19delta	180	360	-0.05976	4678.0	0.95235	-0.01445	0.01359
shed9_diphtheria	5	10	-0.73387	4654.9	0.46307	-0.22593	0.10286
shed9_diphtheria	5	30	-3.61278	4399.6	0.00031	-0.52081	-0.15440
shed9_diphtheria	5	60	0.22801	4664.0	0.81965	-0.13638	0.17227
shed9_diphtheria	5	90	2.31946	4531.8	0.02041	0.02672	0.31858
shed9_diphtheria	5	180	12.54018	2709.2	0.00000	0.62840	0.86134
shed9_diphtheria	5	360	15.50737	2487.1	0.00000	0.78657	1.01429
shed9_diphtheria	10	30	-2.87380	4524.3	0.00407	-0.46440	-0.08774
shed9_diphtheria	10	60	0.97164	4606.3	0.33128	-0.08089	0.23987
shed9_diphtheria	10	90	3.01424	4409.0	0.00259	0.08187	0.38651
shed9_diphtheria	10	180	12.71246	2661.0	0.00000	0.68202	0.93080
shed9_diphtheria	10	360	15.46894	2467.6	0.00000	0.84002	1.08391
shed9_diphtheria	30	60	3.88099	4286.7	0.00011	0.17594	0.53517
shed9_diphtheria	30	90	5.80090	3997.7	0.00000	0.33780	0.68271
shed9_diphtheria	30	180	14.30665	2561.3	0.00000	0.93411	1.23084
shed9_diphtheria	30	360	16.59126	2427.6	0.00000	1.09171	1.38436
shed9_diphtheria	60	90	2.14503	4604.7	0.03200	0.01331	0.29609
shed9_diphtheria	60	180	12.87251	2751.5	0.00000	0.61619	0.83765
shed9_diphtheria	60	360	16.02591	2504.2	0.00000	0.77450	0.99046
shed9_diphtheria	90	180	11.36979	2867.8	0.00000	0.47354	0.67091
shed9_diphtheria	90	360	14.92991	2551.7	0.00000	0.63219	0.82336
shed9_diphtheria	180	360	8.15552	3945.6	0.00000	0.11816	0.19295

blocks	obs.interval.x	obs.interval.y	t	df	p.value	conf.low	conf.high
shed9_fifth	5	10	0.68877	4464.1	0.49100	-0.02604	0.05424
shed9_fifth	5	30	-0.87250	4665.5	0.38298	-0.06244	0.02398
shed9_fifth	5	60	-0.16508	4512.8	0.86889	-0.04402	0.03718
shed9_fifth	5	90	4.94582	3157.1	0.00000	0.05184	0.11995
shed9_fifth	5	180	7.49806	2865.3	0.00000	0.09342	0.15958
shed9_fifth	5	360	6.76496	2937.9	0.00000	0.08164	0.14828
shed9_fifth	10	30	-1.67923	4548.2	0.09318	-0.07225	0.00558
shed9_fifth	10	60	-0.95424	4674.1	0.34001	-0.05352	0.01848
shed9_fifth	10	90	4.95586	3560.3	0.00000	0.04339	0.10020
shed9_fifth	10	180	8.09348	3145.4	0.00000	0.08516	0.13962
shed9_fifth	10	360	7.18567	3251.8	0.00000	0.07334	0.12837
shed9_fifth	30	60	0.78694	4586.9	0.43136	-0.02358	0.05520
shed9_fifth	30	90	6.32285	3240.0	0.00000	0.07253	0.13773
shed9_fifth	30	180	9.04761	2921.1	0.00000	0.11415	0.17731
shed9_fifth	30	360	8.26537	3000.9	0.00000	0.10236	0.16602
shed9_fifth	60	90	6.02773	3501.3	0.00000	0.06026	0.11837
shed9_fifth	60	180	9.12866	3102.9	0.00000	0.10201	0.15782
shed9_fifth	60	360	8.23394	3204.5	0.00000	0.09019	0.14656
shed9_fifth	90	180	4.67993	4450.3	0.00000	0.02359	0.05761
shed9_fifth	90	360	3.26111	4558.2	0.00112	0.01159	0.04653
shed9_fifth	180	360	-1.46089	4657.4	0.14411	-0.02702	0.00395
shed9_flu	5	10	0.55647	4634.5	0.57791	-0.02588	0.04639
shed9_flu	5	30	-0.13507	4672.6	0.89256	-0.03978	0.03465
shed9_flu	5	60	0.69320	4640.9	0.48822	-0.02344	0.04908
shed9_flu	5	90	2.54212	4179.6	0.01105	0.00968	0.07494
shed9_flu	5	180	4.77510	3884.4	0.00000	0.04509	0.10790
shed9_flu	5	360	7.32565	3527.6	0.00000	0.08263	0.14302
shed9_flu	10	30	-0.70848	4659.4	0.47868	-0.04830	0.02266
shed9_flu	10	60	0.14584	4677.7	0.88406	-0.03191	0.03703
shed9_flu	10	90	2.05165	4388.1	0.04026	0.00142	0.06268
shed9_flu	10	180	4.42870	4111.1	0.00001	0.03692	0.09556
shed9_flu	10	360	7.17638	3736.6	0.00000	0.07454	0.13058
shed9_flu	30	60	0.84714	4663.6	0.39696	-0.02022	0.05099
shed9_flu	30	90	2.75784	4255.6	0.00584	0.01297	0.07677
shed9_flu	30	180	5.05730	3963.6	0.00000	0.04841	0.10971
shed9_flu	30	360	7.69339	3598.5	0.00000	0.08598	0.14479
shed9_flu	60	90	1.87848	4373.0	0.06038	-0.00129	0.06026
shed9_flu	60	180	4.23506	4093.5	0.00002	0.03420	0.09315
shed9_flu	60	360	6.95704	3719.6	0.00000	0.07182	0.12818
shed9_flu	90	180	2.69455	4604.4	0.00707	0.00931	0.05906
shed9_flu	90	360	5.92707	4335.2	0.00000	0.04719	0.09384
shed9_flu	180	360	3.29973	4560.5	0.00098	0.01474	0.05791
shed9_mers	5	10	0.62363	4675.7	0.53290	-0.01741	0.03364
shed9_mers	5	30	0.58744	4677.5	0.55694	-0.01798	0.03336
shed9_mers	5	60	-0.39908	4626.1	0.68986	-0.03285	0.02174
shed9_mers	5	90	1.08900	4632.8	0.27621	-0.01094	0.03829
shed9_mers	5	180	1.78384	4615.4	0.07451	-0.00220	0.04664
shed9_mers	5	360	5.64702	4181.4	0.00000	0.04185	0.08636
shed9_mers	10	30	-0.03300	4677.4	0.97368	-0.02582	0.02496
shed9_mers	10	60	-0.99197	4602.9	0.32126	-0.04070	0.01335
shed9_mers	10	90	0.44775	4650.6	0.65435	-0.01877	0.02988
shed9_mers	10	180	1.14596	4636.5	0.25187	-0.01002	0.03823
shed9_mers	10	360	5.00500	4231.1	0.00000	0.03405	0.07791
shed9_mers	30	60	-0.95608	4615.6	0.33908	-0.04041	0.01392
shed9_mers	30	90	0.47917	4641.8	0.63184	-0.01850	0.03046
shed9_mers	30	180	1.17317	4625.9	0.24079	-0.00975	0.03881
shed9_mers	30	360	5.00438	4205.1	0.00000	0.03431	0.07851
shed9_mers	60	90	1.44050	4493.6	0.14980	-0.00694	0.04540
shed9_mers	60	180	2.09549	4462.6	0.03618	0.00179	0.05377
shed9_mers	60	360	5.69918	3934.5	0.00000	0.04570	0.09362
shed9_mers	90	180	0.72334	4676.5	0.46951	-0.01462	0.03171
shed9_mers	90	360	4.73779	4393.4	0.00000	0.02956	0.07129
shed9_mers	180	360	3.97901	4427.9	0.00007	0.02125	0.06252
shed9_sars	5	10	1.11902	4643.2	0.26319	-0.02410	0.08820
shed9_sars	5	30	1.51295	4635.9	0.13036	-0.01277	0.09909
shed9_sars	5	60	1.92619	4290.4	0.05415	-0.00090	0.10175
shed9_sars	5	90	6.86299	3386.6	0.00000	0.11508	0.20714
shed9_sars	5	180	8.73120	3185.6	0.00000	0.15575	0.24596
shed9_sars	5	360	11.12456	2881.9	0.00000	0.20453	0.29205
shed9_sars	10	30	0.40766	4677.6	0.68354	-0.04232	0.06454
shed9_sars	10	60	0.74137	4462.5	0.45851	-0.03022	0.06697
shed9_sars	10	90	5.89027	3558.4	0.00000	0.08610	0.17202
shed9_sars	10	180	7.88612	3332.1	0.00000	0.12683	0.21077
shed9_sars	10	360	10.46298	2981.2	0.00000	0.17572	0.25676
shed9_sars	30	60	0.29467	4477.9	0.76826	-0.04107	0.05560
shed9_sars	30	90	5.42001	3576.7	0.00000	0.07528	0.16062
shed9_sars	30	180	7.41989	3348.0	0.00000	0.11602	0.19936
shed9_sars	30	360	10.00181	2992.1	0.00000	0.16491	0.24534
shed9_sars	60	90	5.95833	4062.7	0.00000	0.07426	0.14710
shed9_sars	60	180	8.36765	3792.3	0.00000	0.11518	0.18567
shed9_sars	60	360	11.57650	3315.8	0.00000	0.16435	0.23137
shed9_sars	90	180	2.89177	4616.0	0.00385	0.01280	0.06669
shed9_sars	90	360	6.93919	4204.8	0.00000	0.06255	0.11181
shed9_sars	180	360	4.06850	4446.0	0.00005	0.02458	0.07029
