# Supplementary material for: Impacts of observation frequency on proximity contact data and modeled transmission dynamics
Source: PLoS Comput Biol. 2023 Feb 27;19(2):e1010917. doi: 10.1371/journal.pcbi.1010917 (PMC9997969; doi:10.1371/journal.pcbi.1010917)
Supplement: S1 Table — It contains two comma-separated values (CSV) files, one for each of the Snapshot and the Upperbound downsampling method. Each CSV file contains details of the corresponding Welch’s t-test, where block names are underscore-concatenated strings of the underlying population and the type of disease/pathogens. (ZIP) [file pcbi.1010917.s001.zip › Welchs-t-test-on-observation-intervals/Welchs-t-test-on-observation-intervals-upperbound.pdf]

number of t-tests per block: 21 (combination 2 out of 7, that is 7*6/2)
Bonferroni-corrected critical value: 0.00238 (0.05 / 21)
Lines with red background-color are cases rejected by Bonferroni-corrected t-test

blocks	obs.interval.x	obs.interval.y	t	df	p.value	conf.low	conf.high
shed1_covid19	5	10	-9.54508801	2051.649111	3.71E-21	-2.972434372	-1.959189559
shed1_covid19	5	30	-27.53853419	1585.217855	9.27E-137	-10.81755217	-9.379029031
shed1_covid19	5	60	-46.82042378	1521.960497	3.82E-297	-19.27505048	-17.72494952
shed1_covid19	5	90	-60.03834925	1545.204434	0	-23.79908994	-22.29321775
shed1_covid19	5	180	-91.59741104	1740.244961	0	-29.73270631	-28.48609711
shed1_covid19	5	360	-125.4727373	2095.522369	0	-32.07566812	-31.08843444
shed1_covid19	10	30	-19.11716273	1980.431003	6.71E-75	-8.4154678	-6.849489465
shed1_covid19	10	60	-37.68244968	1879.804515	5.79E-232	-16.86870593	-15.19967014
shed1_covid19	10	90	-49.58349859	1918.063746	0	-21.3943667	-19.76631706
shed1_covid19	10	180	-75.08153278	2176.880624	0	-27.33949359	-25.9476859
shed1_covid19	10	360	-97.99105664	2334.576588	0	-29.69890863	-28.53357
shed1_covid19	30	60	-16.84431014	2320.413908	3.74E-60	-9.379822579	-7.423596224
shed1_covid19	30	90	-26.42922408	2331.286272	7.35E-135	-13.9085622	-11.9871643
shed1_covid19	30	180	-43.20064233	2270.182754	4.22E-298	-19.87408369	-18.14813854
shed1_covid19	30	360	-54.3905393	1935.839523	0	-22.25841269	-20.70910867
shed1_covid19	60	90	-8.887286952	2335.371417	1.22E-18	-5.549262916	-3.543044777
shed1_covid19	60	180	-22.86401512	2194.156329	5.88E-104	-11.51936971	-9.699433709
shed1_covid19	60	360	-31.03566579	1837.15669	2.18E-170	-13.90875347	-12.25534909
shed1_covid19	90	180	-13.3415373	2225.893596	4.05E-39	-6.954465464	-5.172030262
shed1_covid19	90	360	-20.7700472	1874.45155	2.09E-86	-9.341907221	-7.72988765
shed1_covid19	180	360	-7.063379087	2136.447024	2.19E-12	-3.159155445	-1.7861437
shed1_covid19alpha	5	10	-10.68299226	2092.292126	5.64E-26	-4.109119261	-2.834470482
shed1_covid19alpha	5	30	-31.76642693	1730.774435	7.28E-175	-13.91790022	-12.2991938
shed1_covid19alpha	5	60	-55.37895447	1795.387207	0	-22.51542482	-20.97517347
shed1_covid19alpha	5	90	-63.535305	1819.068472	0	-25.28272212	-23.76855993
shed1_covid19alpha	5	180	-93.86663955	2180.43899	0	-29.52733499	-28.31881886
shed1_covid19alpha	5	360	-113.0957969	2335.278064	0	-30.8462435	-29.79478215
shed1_covid19alpha	10	30	-21.20780869	2129.137911	1.02E-90	-10.5278593	-8.74564497
shed1_covid19alpha	10	60	-41.88072378	2195.119575	3.05E-282	-19.12915255	-17.417856
shed1_covid19alpha	10	90	-48.92263023	2216.323073	0	-21.89777722	-20.20991509
shed1_covid19alpha	10	180	-70.28262499	2322.62215	0	-26.16140927	-24.74115483
shed1_covid19alpha	10	360	-81.67231917	2130.356982	0	-27.49339701	-26.20403888
shed1_covid19alpha	30	60	-17.10175525	2328.721844	7.36E-62	-9.627090593	-7.64641368
shed1_covid19alpha	30	90	-22.84011734	2321.172403	2.52E-104	-12.39733274	-10.43685529
shed1_covid19alpha	30	180	-35.73910377	2036.503691	1.43E-217	-16.68232845	-14.94673138
shed1_covid19alpha	30	360	-41.41307044	1765.120398	1.70E-262	-18.02711863	-16.39681299
shed1_covid19alpha	60	90	-5.750457931	2336.85422	1.01E-08	-3.728474	-1.83220976
shed1_covid19alpha	60	180	-16.93197366	2108.831011	2.13E-60	-8.009120019	-6.346435537
shed1_covid19alpha	60	360	-21.66684194	1832.113567	7.25E-93	-9.351432962	-7.798994388
shed1_covid19alpha	90	180	-10.52601006	2133.292986	2.70E-25	-5.216712025	-3.57815977
shed1_covid19alpha	90	360	-14.88989863	1856.526425	1.87E-47	-6.558150964	-5.031592626
shed1_covid19alpha	180	360	-4.477728056	2213.812348	7.93E-06	-2.009447672	-0.7854241225
shed1_covid19beta	5	10	-10.61244414	2067.962583	1.18E-25	-4.010070659	-2.75916011
shed1_covid19beta	5	30	-31.15352871	1699.289616	6.19E-169	-13.56861227	-11.96130226
shed1_covid19beta	5	60	-53.84491703	1738.339774	0	-22.14141547	-20.58508026
shed1_covid19beta	5	90	-62.16206585	1759.255577	0	-25.0270291	-23.49604783
shed1_covid19beta	5	180	-91.37754336	2090.206819	0	-29.37880435	-28.14427257
shed1_covid19beta	5	360	-114.8528196	2323.192213	0	-31.03727055	-29.99520808
shed1_covid19beta	10	30	-20.73563122	2116.677608	4.22E-87	-10.26749361	-8.493190146
shed1_covid19beta	10	60	-40.80044017	2160.241767	2.60E-270	-18.84277086	-17.1144941
shed1_covid19beta	10	90	-48.0105456	2181.677844	0	-21.72966761	-20.02417854
shed1_covid19beta	10	180	-68.85672249	2337.113775	0	-26.09963577	-24.65421039
shed1_covid19beta	10	360	-82.80778	2156.758021	0	-27.77415855	-26.48908932
shed1_covid19beta	30	60	-16.85146587	2334.221122	3.21E-60	-9.598861287	-7.59771991
shed1_covid19beta	30	90	-22.75501424	2329.345002	1.14E-103	-12.48733424	-10.50582816
shed1_covid19beta	30	180	-35.59185204	2094.855106	2.06E-217	-16.87798653	-15.11517586
shed1_covid19beta	30	360	-42.61353535	1778.796642	4.79E-274	-18.5682892	-16.9342749
shed1_covid19beta	60	90	-5.858000625	2336.991706	5.35E-09	-3.868500248	-1.928080949
shed1_covid19beta	60	180	-16.90510382	2139.462747	2.83E-60	-8.25652792	-6.540053276
shed1_covid19beta	60	360	-22.66739536	1821.554318	2.07E-100	-9.944942242	-8.361040664
shed1_covid19beta	90	180	-10.42177226	2161.605333	7.57E-25	-5.346763774	-3.653236226
shed1_covid19beta	90	360	-15.73708825	1844.256043	1.86E-52	-7.034199504	-5.475202205
shed1_covid19beta	180	360	-5.422699748	2176.997265	6.52E-08	-2.389267358	-1.120134351
shed1_covid19delta	5	10	-14.98426223	2221.053048	1.99E-48	-7.503389483	-5.766695987
shed1_covid19delta	5	30	-37.8006286	2193.949786	3.07E-241	-17.92059596	-16.15290831
shed1_covid19delta	5	60	-57.80109021	2337.204586	0	-23.44626811	-21.90757805
shed1_covid19delta	5	90	-63.87810707	2329.410572	0	-24.84777016	-23.36761445
shed1_covid19delta	5	180	-74.84582893	2174.356517	0	-26.44617147	-25.09570887
shed1_covid19delta	5	360	-80.61678732	2002.826813	0	-27.03960127	-25.75527053
shed1_covid19delta	10	30	-20.88198563	2336.109626	6.33E-89	-11.37851038	-9.424908427
shed1_covid19delta	10	60	-35.96870036	2237.519147	5.59E-224	-16.91648867	-15.16727202
shed1_covid19delta	10	90	-40.3595892	2160.992008	6.39E-266	-18.32164125	-16.62365789
shed1_covid19delta	10	180	-47.31796558	1909.128567	0	-19.92903092	-18.34276395
shed1_covid19delta	10	360	-50.65435366	1743.779198	0	-20.52758864	-18.99719768
shed1_covid19delta	30	60	-12.42767974	2211.747865	2.52E-34	-6.530166894	-4.750174986
shed1_covid19delta	30	90	-16.03384939	2130.471043	1.03E-54	-7.935777154	-6.206103188
shed1_covid19delta	30	180	-21.14584309	1876.787632	3.40E-89	-9.544264085	-7.924111984
shed1_covid19delta	30	360	-23.45535145	1715.783576	9.34E-106	-10.14342856	-8.577938966
shed1_covid19delta	60	90	-3.753872669	2323.468678	1.78E-04	-2.178188757	-0.6833497044
shed1_covid19delta	60	180	-8.880154349	2155.113324	1.37E-18	-3.777290098	-2.41074409
shed1_covid19delta	60	360	-11.21477608	1981.26192	2.42E-28	-4.371130232	-3.069895409
shed1_covid19delta	90	180	-5.016991347	2232.642435	5.66E-07	-2.313373385	-1.013122341
shed1_covid19delta	90	360	-7.29313205	2073.399683	4.29E-13	-2.905451113	-1.674036066
shed1_covid19delta	180	360	-2.292307973	2285.35084	0.02197837538	-1.162444533	-0.09054691965
shed1_diphtheria	5	10	-12.3068982	2135.221341	1.12E-33	-5.100138878	-3.698151721
shed1_diphtheria	5	30	-33.42835947	1907.185756	3.10E-193	-14.52548167	-12.91554397
shed1_diphtheria	5	60	-52.1069013	1958.834433	0	-21.4808709	-19.92254791
shed1_diphtheria	5	90	-69.58186997	2149.996377	0	-25.34604406	-23.95652004
shed1_diphtheria	5	180	-104.1133516	2278.774315	0	-29.26669691	-28.18458514
shed1_diphtheria	5	360	-127.8679397	1735.50734	0	-30.61983911	-29.69469081
shed1_diphtheria	10	30	-20.44626122	2251.688474	2.29E-85	-10.21538771	-8.42734733
shed1_diphtheria	10	60	-36.7098961	2284.886837	2.61E-232	-17.17342937	-15.43169884
shed1_diphtheria	10	90	-49.87420967	2337.560616	0	-21.04842049	-19.45585302
shed1_diphtheria	10	180	-71.57928283	1948.399303	0	-24.9930111	-23.65998036
shed1_diphtheria	10	360	-83.57056917	1482.755098	0	-26.36271325	-25.15352607
shed1_diphtheria	30	60	-14.31277082	2333.347394	1.33E-44	-7.937684694	-6.024708469
shed1_diphtheria	30	90	-24.10792275	2240.206725	2.30E-114	-11.81991657	-10.04162189
shed1_diphtheria	30	180	-37.96724373	1732.478692	4.86E-230	-15.78027195	-14.22998446
shed1_diphtheria	30	360	-44.62098877	1382.154872	4.62E-270	-17.15936449	-15.71413978
shed1_diphtheria	60	90	-8.945009404	2275.428196	7.52E-19	-4.815434229	-3.08371107
shed1_diphtheria	60	180	-21.03063045	1777.776753	7.23E-88	-8.772236881	-7.275626367
shed1_diphtheria	60	360	-26.73705347	1401.699277	1.34E-127	-10.1492956	-8.761815508
shed1_diphtheria	90	180	-12.10763533	1964.357562	1.35E-32	-4.734316104	-3.414401845
shed1_diphtheria	90	360	-18.08022482	1491.144734	3.20E-66	-6.10333691	-4.908628902
shed1_diphtheria	180	360	-6.875869925	1910.693376	8.32E-12	-1.839966433	-1.02328143
shed1_fifth	5	10	-9.221094794	2047.574616	7.08E-20	-1.587882538	-1.030920881
shed1_fifth	5	30	-22.22837066	1460.483668	1.58E-94	-5.719343179	-4.791767933
shed1_fifth	5	60	-34.62400041	1322.511604	1.58E-187	-11.66117975	-10.41061512
shed1_fifth	5	90	-45.48954214	1288.768588	2.77E-270	-17.01990221	-15.61257642
shed1_fifth	5	180	-76.47476136	1302.727053	0	-26.69239323	-25.35717942

shed1_fifth	5	360	-129.424944	1431.646415	0	-32.58738962	-31.61431978
shed1_fifth	10	30	-15.66039541	1775.547126	7.07E-52	-4.4403684	-3.451939293
shed1_fifth	10	60	-29.43554256	1502.401877	9.55E-151	-10.37465607	-9.078335379
shed1_fifth	10	90	-40.65620822	1430.813293	7.50E-241	-15.73090324	-14.28277198
shed1_fifth	10	180	-70.3568298	1460.597753	0	-25.40446397	-24.02630526
shed1_fifth	10	360	-117.1265231	1721.614565	0	-31.30707145	-30.27583454
shed1_fifth	30	60	-15.18392422	2126.61591	1.62E-49	-6.526902085	-5.033781676
shed1_fifth	30	90	-26.6709646	1983.140589	3.49E-134	-11.87399446	-10.24737307
shed1_fifth	30	180	-52.06332817	2047.808801	0	-21.55156681	-19.98689472
shed1_fifth	30	360	-82.90620564	2331.31153	0	-27.48027152	-26.21032677
shed1_fifth	60	90	-11.31770525	2302.418866	6.14E-29	-6.195255552	-4.365428209
shed1_fifth	60	180	-33.11864265	2326.792768	2.13E-197	-15.87639422	-14.10138355
shed1_fifth	60	360	-54.29193083	2183.276954	0	-21.82583384	-20.30408069
shed1_fifth	90	180	-20.16105591	2330.848968	1.87E-83	-10.6528571	-8.764236914
shed1_fifth	90	360	-37.45530106	2045.684551	2.87E-234	-16.61108299	-14.95814778
shed1_fifth	180	360	-14.96935917	2108.994163	3.19E-48	-6.872075305	-5.280061447
shed1_flu	5	10	-8.540531214	2065.512819	2.55E-17	-1.801346993	-1.128567537
shed1_flu	5	30	-23.36203601	1504.415044	3.03E-103	-6.855833134	-5.793739515
shed1_flu	5	60	-38.06096072	1362.285637	1.60E-216	-13.97381393	-12.60396385
shed1_flu	5	90	-52.3011184	1348.29954	0	-19.62486816	-18.20590107
shed1_flu	5	180	-81.82242664	1388.262834	0	-27.58167951	-26.29011536
shed1_flu	5	360	-130.2144976	1594.729225	0	-32.11850969	-31.165251
shed1_flu	10	30	-16.76990847	1836.745418	7.68E-59	-5.428190586	-4.291467534
shed1_flu	10	60	-32.47174325	1572.820862	1.88E-177	-12.53816268	-11.10970057
shed1_flu	10	90	-46.39253672	1544.869646	4.92E-295	-18.18824056	-16.71261414
shed1_flu	10	180	-73.81831091	1623.924985	0	-26.14772836	-24.79415198
shed1_flu	10	360	-114.2748155	1980.545944	0	-30.69481365	-29.6590325
shed1_flu	30	60	-16.57826717	2173.100041	3.23E-58	-7.787891722	-6.140313406
shed1_flu	30	90	-29.244219	2132.725919	2.25E-158	-13.43490642	-11.74629016
shed1_flu	30	180	-51.06225712	2234.430994	0	-21.40267286	-19.81954936
shed1_flu	30	360	-75.11251775	2301.803883	0	-25.97805839	-24.65612965
shed1_flu	60	90	-11.6321383	2334.624678	1.92E-30	-6.575027186	-4.677964267
shed1_flu	60	180	-29.67159607	2328.472274	2.20E-164	-14.54893361	-12.74508349
shed1_flu	60	360	-45.56798881	2032.791526	0.00E-02	-19.14285806	-17.56312484
shed1_flu	90	180	-17.08285122	2314.04573	1.03E-61	-8.941210579	-7.099815062
shed1_flu	90	360	-30.76579651	1988.373359	2.42E-170	-13.53774292	-11.91524854
shed1_flu	180	360	-12.20443307	2106.212028	3.77E-33	-5.462172104	-3.949793708
shed1_mers	5	10	-4.966438444	2206.933207	7.34E-07	-0.5340995915	-0.2317123743
shed1_mers	5	30	-13.85760958	1597.623405	2.54E-41	-1.877204321	-1.411684568
shed1_mers	5	60	-20.49734237	1340.615842	1.94E-81	-4.061607181	-3.352068033
shed1_mers	5	90	-25.44033567	1262.552055	1.34E-115	-6.618276471	-5.670612418
shed1_mers	5	180	-38.08601322	1211.340969	2.51E-209	-14.23407067	-12.8394336
shed1_mers	5	360	-63.32307005	1205.554859	0	-24.94361843	-23.44441576
shed1_mers	10	30	-10.12296332	1834.362715	1.78E-23	-1.505953304	-1.017123619
shed1_mers	10	60	-17.98601361	1448.631339	1.93E-65	-3.686448529	-2.961414718
shed1_mers	10	90	-23.56417093	1322.421988	8.56E-103	-6.24119687	-5.281880053
shed1_mers	10	180	-36.79831614	1238.588522	6.46E-201	-13.85513592	-12.45255639
shed1_mers	10	360	-62.0139587	1229.087446	0	-24.56440801	-23.05781421
shed1_mers	30	60	-10.02874951	1958.273951	4.07E-23	-2.465705284	-1.659081041
shed1_mers	30	90	-17.26687177	1641.290502	1.63E-61	-5.011172315	-3.988827685
shed1_mers	30	180	-32.25777084	1389.976768	6.98E-171	-12.61550735	-11.16910804
shed1_mers	30	360	-57.17170921	1360.202315	0	-23.32330695	-21.77583835
shed1_mers	60	90	-8.286056176	2149.491308	2.03E-16	-3.014517402	-1.860696273
shed1_mers	60	180	-25.00540467	1710.280143	7.10E-118	-10.60094475	-9.058884309
shed1_mers	60	360	-49.08763265	1643.136737	0	-21.30579166	-19.66856731
shed1_mers	90	180	-17.41582717	2046.518733	1.87E-63	-8.224724703	-6.559890682
shed1_mers	90	360	-40.37848375	1960.758719	5.89E-260	-18.92623666	-17.17290864
shed1_mers	180	360	-20.59354896	2325.478115	1.07E-86	-11.67208435	-9.642445569
shed1_sars	5	10	-9.964266869	2089.974523	7.01E-23	-2.851893443	-1.913918522
shed1_sars	5	30	-28.4934606	1621.032253	4.10E-145	-10.18867394	-8.876283323
shed1_sars	5	60	-47.20648156	1534.600455	3.35E-301	-18.10608604	-16.66143533
shed1_sars	5	90	-63.57133444	1572.67795	0	-23.07352352	-21.69228845
shed1_sars	5	180	-93.19794009	1736.846988	0	-28.71579377	-27.53206948
shed1_sars	5	360	-138.4164624	2178.82435	0	-31.81799704	-30.92901151
shed1_sars	10	30	-19.70655822	1993.330915	3.97E-79	-7.861082927	-6.438062372
shed1_sars	10	60	-38.06425932	1863.045269	4.98E-235	-15.77376474	-14.22794466
shed1_sars	10	90	-52.76516574	1922.634686	0	-20.74336869	-19.25663131
shed1_sars	10	180	-77.34396887	2138.057371	0	-26.39369528	-25.08835601
shed1_sars	10	360	-108.7412735	2322.451917	0	-29.5134003	-28.46779628
shed1_sars	30	60	-17.1311758	2308.646522	5.03E-62	-8.750012066	-6.952552037
shed1_sars	30	90	-28.84985264	2329.414952	9.48E-157	-13.72389692	-11.97695778
shed1_sars	30	180	-45.70019813	2301.43305	0	-19.38921216	-17.79369383
shed1_sars	30	360	-61.57668093	1898.307973	0	-22.5366613	-21.14538998
shed1_sars	60	90	-10.6080161	2331.59569	1.04E-25	-5.923279803	-4.075010796
shed1_sars	60	180	-24.69289983	2215.184019	4.01E-119	-11.59312286	-9.887219025
shed1_sars	60	360	-36.18248134	1774.599486	3.37E-215	-14.74806935	-13.23141783
shed1_sars	90	180	-13.62508214	2260.354199	1.10E-40	-6.567312913	-4.914738369
shed1_sars	90	360	-24.21467062	1830.420201	1.34E-112	-9.718789425	-8.262407156
shed1_sars	180	360	-10.03085952	2045.481513	3.79E-23	-3.884893707	-2.614251592
shed2_covid19	5	10	-2.90059992	1813.992142	0.003769348387	-0.4766582905	-0.09209170946
shed2_covid19	5	30	-7.740869686	1452.032003	1.84E-14	-1.259936268	-0.7504803989
shed2_covid19	5	60	-11.94707897	1193.634532	3.71E-31	-2.529753786	-1.816079547
shed2_covid19	5	90	-15.00418011	1089.557304	2.12E-46	-4.054292838	-3.116540495
shed2_covid19	5	180	-21.80081632	1021.428031	8.01E-87	-8.087646339	-6.751936995
shed2_covid19	5	360	-34.11363822	997.6873994	1.13E-169	-15.50814524	-13.82102142
shed2_covid19	10	30	-5.2069857	1677.811427	2.16E-07	-0.9923584284	-0.4493082382
shed2_covid19	10	60	-10.04013139	1331.992972	6.47E-23	-2.257544819	-1.519538514
shed2_covid19	10	90	-13.54388092	1170.138729	6.26E-39	-3.779237094	-2.822846239
shed2_covid19	10	180	-20.76003606	1060.559174	1.33E-80	-7.809844117	-6.460989216
shed2_covid19	10	360	-33.2457131	1022.005498	7.08E-165	-15.22898268	-13.53143398
shed2_covid19	30	60	-5.655414671	1675.555092	1.82E-08	-1.57268672	-0.7627299469
shed2_covid19	30	90	-9.993973143	1405.693276	9.12E-23	-3.086660915	-2.073755752
shed2_covid19	30	180	-18.11515241	1181.733834	6.48E-65	-7.109318986	-5.71984768
shed2_covid19	30	360	-30.98449799	1098.113125	5.59E-152	-14.52436961	-12.79438039
shed2_covid19	60	90	-4.906645745	1769.325956	1.01E-06	-1.977110611	-0.8478893895
shed2_covid19	60	180	-13.94321669	1429.706136	1.59E-41	-5.985040339	-4.508709661
shed2_covid19	60	360	-27.22267634	1262.557586	8.98E-129	-13.39189814	-11.59143519
shed2_covid19	90	180	-9.421704401	1703.76101	1.39E-20	-4.632593581	-3.036156419
shed2_covid19	90	360	-22.87481945	1479.609788	2.09E-99	-12.02923079	-10.12910254
shed2_covid19	180	360	-13.37697169	1817.543806	5.40E-39	-8.30698969	-6.182593643
shed2_covid19alpha	5	10	-3.565112334	1744.295532	3.73E-04	-0.6701143054	-0.1944690279
shed2_covid19alpha	5	30	-9.568501567	1336.723292	5.02E-21	-1.982007897	-1.307575437
shed2_covid19alpha	5	60	-14.61072425	1131.780309	2.03E-44	-4.073988654	-3.10934468
shed2_covid19alpha	5	90	-16.83487168	1081.432744	1.17E-56	-5.432729705	-4.298520295
shed2_covid19alpha	5	180	-24.4884464	1023.51516	1.31E-104	-10.39513603	-8.852780633
shed2_covid19alpha	5	360	-37.90333516	1009.380148	3.22E-196	-17.66976318	-15.93023682
shed2_covid19alpha	10	30	-6.557577167	1613.730356	7.34E-11	-1.575170545	-0.8498294547
shed2_covid19alpha	10	60	-12.38548993	1283.943593	2.32E-33	-3.659807689	-2.658942311
shed2_covid19alpha	10	90	-14.93011601	1191.766497	2.46E-46	-5.015914691	-3.850751976
shed2_covid19alpha	10	180	-23.04488052	1082.629749	6.20E-96	-9.974291625	-8.409041708
shed2_covid19alpha	10	360	-36.49951649	1055.65781	2.56E-189	-17.2476362	-15.48778046

shed2_covid19alpha	30	60	-6.886918241	1668.80621	8.05E-12	-2.501342801	-1.392407199
shed2_covid19alpha	30	90	-10.03486353	1504.777734	5.55E-23	-3.850418275	-2.591248391
shed2_covid19alpha	30	180	-19.13220836	1265.459636	7.23E-72	-8.797360315	-7.160973018
shed2_covid19alpha	30	360	-32.61331066	1200.685428	1.34E-167	-16.06691079	-14.24350588
shed2_covid19alpha	60	90	-3.481499342	1862.549317	5.10E-04	-1.991619273	-0.5562973937
shed2_covid19alpha	60	180	-13.32911285	1584.177498	1.71E-38	-6.919981234	-5.144602099
shed2_covid19alpha	60	360	-26.58621514	1471.172072	1.67E-127	-14.18286744	-12.23379923
shed2_covid19alpha	90	180	-9.967010311	1748.704415	8.51E-23	-5.694684472	-3.821982195
shed2_covid19alpha	90	360	-22.97090635	1632.320444	2.08E-101	-12.9534163	-10.9153337
shed2_covid19alpha	180	360	-12.29161676	1889.303612	1.84E-33	-8.321033383	-6.031049951
shed2_covid19beta	5	10	-3.521348162	1757.664884	4.40E-04	-0.671446899	-0.191053101
shed2_covid19beta	5	30	-9.359686027	1373.265165	3.15E-20	-1.903843648	-1.244073019
shed2_covid19beta	5	60	-14.09772604	1150.479433	9.45E-42	-3.838776944	-2.90080639
shed2_covid19beta	5	90	-16.87428517	1088.21309	6.31E-57	-5.41279649	-4.285120177
shed2_covid19beta	5	180	-24.09069192	1027.184208	5.19E-102	-10.16679136	-8.635291977
shed2_covid19beta	5	360	-37.69113222	1011.959165	6.11E-195	-17.4949299	-15.76340344
shed2_covid19beta	10	30	-6.308698374	1647.969983	3.61E-10	-1.497981826	-0.7874348403
shed2_covid19beta	10	60	-11.83341931	1307.492829	9.15E-31	-3.425701982	-2.451381352
shed2_covid19beta	10	90	-14.96845441	1197.34465	1.45E-46	-4.996746783	-3.838669884
shed2_covid19beta	10	180	-22.65116425	1085.795791	2.44E-93	-9.746797533	-8.1927858
shed2_covid19beta	10	360	-36.29379846	1057.60567	5.53E-188	-17.07365049	-15.32218284
shed2_covid19beta	30	60	-6.5598013	1670.368352	7.17E-11	-2.332788764	-1.258877903
shed2_covid19beta	30	90	-10.33657206	1483.896781	3.12E-24	-3.896494426	-2.653505574
shed2_covid19beta	30	180	-18.97820115	1252.373556	8.62E-71	-8.636203314	-7.017963352
shed2_covid19beta	30	360	-32.66207582	1188.940829	1.53E-167	-15.95955182	-14.15086485
shed2_covid19beta	60	90	-4.114484167	1846.463701	4.05E-05	-2.18424047	-0.7740928635
shed2_covid19beta	60	180	-13.52178946	1560.972685	1.77E-39	-6.906149416	-5.156350584
shed2_covid19beta	60	360	-26.99140141	1447.83755	2.60E-130	-14.22300191	-12.29574809
shed2_covid19beta	90	180	-9.609672362	1749.185735	2.42E-21	-5.48115738	-3.623009286
shed2_covid19beta	90	360	-22.80829074	1630.223234	3.64E-100	-12.79325833	-10.76715833
shed2_covid19beta	180	360	-12.46100643	1888.060655	2.62E-34	-8.365749984	-6.090500016
shed2_covid19delta	5	10	-4.364003723	1767.871424	1.35E-05	-1.185209598	-0.4502070684
shed2_covid19delta	5	30	-10.18837431	1392.151354	1.46E-23	-3.08693945	-2.090143883
shed2_covid19delta	5	60	-15.97174287	1192.599565	3.47E-52	-6.029412247	-4.710171086
shed2_covid19delta	5	90	-20.04774695	1127.03696	1.06E-76	-8.618278752	-7.081721248
shed2_covid19delta	5	180	-26.37771104	1082.369172	8.16E-119	-12.83220943	-11.05529057
shed2_covid19delta	5	360	-41.98781614	1084.516312	1.44E-229	-19.73852126	-17.97606207
shed2_covid19delta	10	30	-6.475587629	1660.616968	1.24E-10	-2.307201813	-1.234464854
shed2_covid19delta	10	60	-12.96549691	1370.355512	2.40E-36	-5.24081949	-3.863347177
shed2_covid19delta	10	90	-17.38863217	1259.846666	7.21E-61	-7.825700399	-6.238882935
shed2_covid19delta	10	180	-23.98073648	1181.693881	7.21E-104	-12.03631393	-10.21576941
shed2_covid19delta	10	360	-39.18555171	1185.493389	4.06E-216	-18.94280064	-17.13636602
shed2_covid19delta	30	60	-7.117231344	1742.363843	1.60E-12	-3.547691176	-2.014808824
shed2_covid19delta	30	90	-11.97656829	1583.063865	1.06E-31	-6.123154187	-4.39976248
shed2_covid19delta	30	180	-18.9120537	1442.581144	1.95E-71	-10.32555606	-8.384860603
shed2_covid19delta	30	360	-33.1137122	1449.883814	1.54E-179	-17.23248383	-15.30501617
shed2_covid19delta	60	90	-5.047173242	1866.055707	4.92E-07	-3.443970387	-1.51644628
shed2_covid19delta	60	180	-12.14112446	1746.09618	1.27E-32	-7.635940622	-5.511976045
shed2_covid19delta	60	360	-25.05175591	1753.878426	1.18E-118	-14.54344473	-12.43155527
shed2_covid19delta	90	180	-7.089128042	1872.977346	1.90E-12	-5.226299851	-2.961200149
shed2_covid19delta	90	360	-19.15694412	1877.679282	7.38E-75	-12.13418386	-9.880399476
shed2_covid19delta	180	360	-11.18804849	1917.854818	3.41E-28	-8.125446462	-5.701636871
shed2_diphtheria	5	10	-4.312522406	1834.154219	1.70E-05	-0.9759163589	-0.3657503078
shed2_diphtheria	5	30	-13.49808217	1437.797691	3.50E-39	-3.282073332	-2.449176668
shed2_diphtheria	5	60	-21.58174964	1211.30994	1.15E-87	-6.68180317	-5.56819683
shed2_diphtheria	5	90	-28.20786171	1133.97319	4.59E-133	-10.13739627	-8.818853734
shed2_diphtheria	5	180	-44.14590553	1091.991614	4.30E-245	-17.61959784	-16.11998549
shed2_diphtheria	5	360	-68.2110777	1131.036809	0	-23.76553364	-22.43654969
shed2_diphtheria	10	30	-9.79151662	1635.695639	4.87E-22	-2.634447569	-1.755135765
shed2_diphtheria	10	60	-18.62905559	1339.274241	4.68E-69	-6.028518909	-4.879814425
shed2_diphtheria	10	90	-25.63073101	1226.041076	2.27E-116	-9.481444592	-8.133138742
shed2_diphtheria	10	180	-41.65889802	1162.963105	7.36E-233	-16.96187943	-15.43603724
shed2_diphtheria	10	360	-64.7852964	1221.660801	0	-23.10946738	-21.75094928
shed2_diphtheria	30	60	-9.983248901	1726.747373	7.41E-23	-3.899721506	-2.619028494
shed2_diphtheria	30	90	-17.73931256	1548.877148	3.05E-64	-7.343666856	-5.881333144
shed2_diphtheria	30	180	-33.75960354	1426.844353	3.95E-184	-14.81788964	-13.19044369
shed2_diphtheria	30	360	-53.93822985	1540.895496	0	-20.97129297	-19.49954036
shed2_diphtheria	60	90	-8.028723759	1854.188892	1.73E-15	-4.172221187	-2.534028813
shed2_diphtheria	60	180	-23.58503196	1744.357268	5.57E-107	-11.63832564	-9.851257697
shed2_diphtheria	60	360	-40.43993816	1848.566275	1.08E-256	-17.79934237	-16.15274096
shed2_diphtheria	90	180	-15.0910738	1882.116292	1.16E-48	-8.352282405	-6.431050928
shed2_diphtheria	90	360	-29.828922	1917.858026	6.10E-161	-14.51860063	-12.7272327
shed2_diphtheria	180	360	-12.67461189	1886.303472	2.17E-35	-7.195450501	-5.267049499
shed2_fifth	5	10	-1.835024883	1901.671451	0.06665811278	-0.2370461059	0.007879439234
shed2_fifth	5	30	-7.83442874	1412.14911	9.20E-15	-0.9195560209	-0.5512773124
shed2_fifth	5	60	-10.51526252	1231.408586	7.98E-25	-1.474567493	-1.010849174
shed2_fifth	5	90	-13.1090318	1115.336173	1.28E-36	-2.301744985	-1.702421682
shed2_fifth	5	180	-18.54224598	1016.724101	2.42E-66	-5.043039186	-4.077794147
shed2_fifth	5	360	-28.63278738	983.5874679	1.24E-131	-11.43667514	-9.969574861
shed2_fifth	10	30	-6.482207948	1490.505914	1.23E-10	-0.8087013998	-0.4329652668
shed2_fifth	10	60	-9.424587273	1284.034739	1.94E-20	-1.362954438	-0.8932955624
shed2_fifth	10	90	-12.26415621	1146.702985	1.40E-32	-2.189464589	-1.585535411
shed2_fifth	10	180	-18.02261746	1028.486032	2.56E-63	-4.929888422	-3.961778244
shed2_fifth	10	360	-28.28970618	988.607329	1.83E-129	-11.32303495	-9.854048379
shed2_fifth	30	60	-3.657194167	1789.528648	2.62E-04	-0.7793434868	-0.2352398465
shed2_fifth	30	90	-7.490104857	1525.197511	1.16E-13	-1.59838335	-0.9349499834
shed2_fifth	30	180	-14.91549407	1185.771486	3.08E-46	-4.328136017	-3.321863983
shed2_fifth	30	360	-26.17601768	1056.655065	8.04E-117	-10.71490957	-9.220507095
shed2_fifth	60	90	-4.133259056	1781.094705	3.74E-05	-1.119710448	-0.3990395525
shed2_fifth	60	180	-12.45822875	1340.938878	8.64E-34	-3.84013119	-2.795285476
shed2_fifth	60	360	-24.41363596	1127.264736	5.13E-106	-10.22072971	-8.70010362
shed2_fifth	90	180	-9.029431236	1579.472346	4.89E-19	-3.114081134	-2.002585533
shed2_fifth	90	360	-21.78539553	1251.539573	1.80E-89	-9.484604978	-7.917478355
shed2_fifth	180	360	-13.85082602	1650.151213	2.36E-41	-7.012571565	-5.272845102
shed2_flu	5	10	-3.697794724	1849.628495	2.24E-04	-0.3507127657	-0.1076205676
shed2_flu	5	30	-8.672583343	1454.042215	1.11E-17	-0.8979240912	-0.5666592422
shed2_flu	5	60	-12.0082139	1182.745056	1.99E-31	-1.691756863	-1.21657647
shed2_flu	5	90	-16.23464019	1063.873868	4.04E-53	-3.143091673	-2.465241661
shed2_flu	5	180	-23.56141803	1000.708217	5.33E-98	-6.889022213	-5.829727787
shed2_flu	5	360	-36.27053085	980.8898911	2.18E-183	-14.17440643	-12.71934357
shed2_flu	10	30	-5.672049297	1632.7154	1.67E-08	-0.6771076969	-0.3291423031
shed2_flu	10	60	-9.870344291	1283.973782	3.37E-22	-1.468478973	-0.981521027
shed2_flu	10	90	-14.72675062	1113.254132	5.53E-45	-2.918076379	-2.231923621
shed2_flu	10	180	-22.59811418	1020.529224	5.28E-92	-6.662520634	-5.597896032
shed2_flu	10	360	-35.55700757	991.3056772	3.58E-179	-13.94718202	-12.48823465
shed2_flu	30	60	-5.27999975	1649.895435	1.46E-07	-0.990035559	-0.453714441
shed2_flu	30	90	-11.25917305	1323.132902	3.80E-28	-2.432871177	-1.710878823
shed2_flu	30	180	-20.29484562	1108.155569	4.10E-78	-6.171110124	-5.083056543
shed2_flu	30	360	-33.8036358	1037.601689	1.70E-169	-13.45264679	-11.97651988
shed2_flu	60	90	-6.635123422	1690.928317	4.35E-11	-1.749065174	-0.9509348262
shed2_flu	60	180	-16.88325061	1300.223887	5.64E-58	-5.475180887	-4.33523578

shed2_flu	60	360	-31.06781566	1142.37307	4.17E-154	-12.75009041	-11.23532626
shed2_flu	90	180	-11.26613665	1616.405618	2.14E-28	-4.174169567	-2.9362471
shed2_flu	90	360	-26.26594027	1341.614094	4.99E-123	-11.43758414	-9.847832529
shed2_flu	180	360	-15.57057018	1748.082229	2.72E-51	-7.980265607	-6.194734393
shed2_mers	5	10	-1.037180666	1902.194051	0.2997834927	-0.1114203631	0.03433702976
shed2_mers	5	30	-5.536944003	1599.738477	3.59E-08	-0.3569006161	-0.1701827173
shed2_mers	5	60	-9.687012964	1369.012559	1.65E-21	-0.6814212412	-0.4519120922
shed2_mers	5	90	-11.50941372	1171.215349	4.07E-29	-1.063175478	-0.7534911891
shed2_mers	5	180	-16.43702724	1032.136856	4.20E-54	-2.4031706	-1.8905794
shed2_mers	5	360	-23.72453072	979.7838118	1.12E-98	-6.214336256	-5.264830411
shed2_mers	10	30	-4.601098362	1687.133323	4.52E-06	-0.3209137268	-0.1290862732
shed2_mers	10	60	-8.866552465	1441.045238	2.18E-18	-0.6449659153	-0.4112840847
shed2_mers	10	90	-10.91135175	1212.398432	1.66E-26	-1.026185114	-0.7133982195
shed2_mers	10	180	-16.0827182	1046.751446	3.54E-52	-2.365568518	-1.851098149
shed2_mers	10	360	-23.53991953	983.9516776	1.52E-97	-6.176302339	-5.225780994
shed2_mers	30	60	-4.553027615	1795.750344	5.64E-06	-0.433700705	-0.172549295
shed2_mers	30	90	-7.57845339	1475.713699	6.14E-14	-0.8116866444	-0.4778966889
shed2_mers	30	180	-14.01032121	1148.308477	2.74E-41	-2.147078913	-1.619587754
shed2_mers	30	360	-22.44228173	1013.215534	7.18E-91	-5.954855758	-4.997227575
shed2_mers	60	90	-3.728892373	1725.205957	1.99E-04	-0.5213780749	-0.1619552584
shed2_mers	60	180	-11.39631026	1276.011074	1.01E-28	-1.85223427	-1.308182396
shed2_mers	60	360	-20.99704927	1051.353457	5.08E-82	-5.656337723	-4.68949561
shed2_mers	90	180	-8.343982783	1544.716779	1.57E-16	-1.52969766	-0.9473856735
shed2_mers	90	360	-19.17196579	1142.817096	2.99E-71	-5.325675836	-4.336824164
shed2_mers	180	360	-13.17746267	1462.749496	1.49E-37	-4.12751617	-3.057900497
shed2_sars	5	10	-3.501342534	1834.158	4.74E-04	-0.4859200453	-0.1369966213
shed2_sars	5	30	-9.193082288	1464.721707	1.27E-19	-1.320810544	-0.8562727892
shed2_sars	5	60	-15.57630873	1162.142834	7.96E-50	-3.144480657	-2.440936009
shed2_sars	5	90	-19.24479488	1067.977953	4.33E-71	-5.093118601	-4.150631399
shed2_sars	5	180	-29.04652356	1011.48043	2.20E-135	-10.59328465	-9.252548686
shed2_sars	5	360	-47.05942549	999.9641807	8.39E-256	-18.90249959	-17.38916707
shed2_sars	10	30	-6.199349232	1666.220498	7.13E-10	-1.022941801	-0.5312248654
shed2_sars	10	60	-13.48875682	1267.782529	7.58E-39	-2.842128913	-2.120371087
shed2_sars	10	90	-17.69036224	1126.499315	5.49E-62	-4.788493267	-3.832340066
shed2_sars	10	180	-27.93308495	1039.940079	1.47E-128	-10.28664557	-8.936271092
shed2_sars	10	360	-45.99077762	1022.204261	3.65E-251	-18.59531386	-17.07343614
shed2_sars	30	60	-8.526671154	1590.532397	3.45E-17	-2.096189402	-1.312143931
shed2_sars	30	90	-13.80824156	1327.741214	1.26E-40	-4.035317942	-3.031348725
shed2_sars	30	180	-25.03702345	1141.52918	1.26E-110	-9.526687135	-8.142062865
shed2_sars	30	360	-43.11986397	1101.944388	8.70E-239	-17.83346383	-16.2811195
shed2_sars	60	90	-6.326208444	1754.370212	3.18E-10	-2.39626399	-1.262069343
shed2_sars	60	180	-18.87978339	1419.181149	4.17E-71	-7.871047354	-6.389369313
shed2_sars	60	360	-36.74223013	1326.918213	1.88E-204	-16.17286417	-14.53338583
shed2_sars	90	180	-12.92698005	1707.604495	1.54E-36	-6.105345553	-4.49673778
shed2_sars	90	360	-30.22993641	1589.382499	5.61E-159	-14.40145511	-12.64646155
shed2_sars	180	360	-16.15236311	1889.238156	4.49E-55	-9.221343475	-7.224489859
shed7_covid19	5	10	-3.163124594	3584.56877	0.00157390361	-0.3239676748	-0.07603232519
shed7_covid19	5	30	-10.61433936	2884.873026	7.61E-26	-1.059783043	-0.7292879956
shed7_covid19	5	60	-16.30731412	2359.382256	9.87E-57	-2.104602598	-1.652774452
shed7_covid19	5	90	-19.28617117	2169.85358	1.26E-76	-2.999826104	-2.446075535
shed7_covid19	5	180	-26.10230408	1980.850919	2.47E-129	-5.812771959	-5.000342795
shed7_covid19	5	360	-32.43557785	1914.528004	2.61E-184	-9.415534766	-8.341842283
shed7_covid19	10	30	-7.928017989	3143.578813	3.07E-15	-0.866304671	-0.5227663672
shed7_covid19	10	60	-14.2669839	2524.90827	1.79E-44	-1.909413324	-1.447963725
shed7_covid19	10	90	-17.6185239	2280.637362	3.06E-65	-2.803764267	-2.242137373
shed7_covid19	10	180	-24.97075591	2031.307223	3.01E-120	-5.615465663	-4.797649091
shed7_covid19	10	360	-31.58457031	1943.051574	4.56E-177	-9.21757568	-8.139801369
shed7_covid19	30	60	-7.559442151	3229.993399	5.24E-14	-1.239413577	-0.7288924339
shed7_covid19	30	90	-11.8993244	2823.964232	6.81E-32	-2.129706754	-1.527123847
shed7_covid19	30	180	-20.90614924	2299.588007	5.35E-89	-4.935249363	-4.088794353
shed7_covid19	30	360	-28.47733966	2096.821154	4.40E-151	-8.533982823	-7.434323188
shed7_covid19	60	90	-4.892299032	3483.692118	1.04E-06	-1.182610133	-0.5059144569
shed7_covid19	60	180	-15.36046515	2780.626065	3.59E-51	-3.97821374	-3.077523965
shed7_covid19	60	360	-24.04144977	2392.476738	1.41E-114	-7.57095942	-6.42904058
shed7_covid19	90	180	-11.00998319	3182.336721	1.07E-27	-3.161515865	-2.20569725
shed7_covid19	90	360	-20.35716251	2686.243594	8.62E-86	-6.74867215	-5.56280326
shed7_covid19	180	360	-10.26526855	3392.166329	2.29E-24	-4.135307275	-2.80895502
shed7_covid19alpha	5	10	-4.010638714	3504.932243	6.18E-05	-0.4295727365	-0.1474764439
shed7_covid19alpha	5	30	-11.88025763	2743.20525	8.89E-32	-1.36240747	-0.976390344
shed7_covid19alpha	5	60	-17.6718691	2245.938616	1.54E-65	-2.760422823	-2.208976084
shed7_covid19alpha	5	90	-20.66619977	2106.430393	1.52E-86	-3.845290379	-3.178753336
shed7_covid19alpha	5	180	-27.68496134	1970.37712	8.81E-143	-6.948163708	-6.028885472
shed7_covid19alpha	5	360	-33.7266635	1917.133244	3.56E-196	-10.52367662	-9.367033767
shed7_covid19alpha	10	30	-8.487849954	3111.553276	3.21E-17	-1.084359745	-0.6773888892
shed7_covid19alpha	10	60	-15.20930032	2455.225786	5.42E-50	-2.47932705	-1.913022677
shed7_covid19alpha	10	90	-18.62289569	2249.766096	4.19E-72	-3.56293639	-2.884058145
shed7_covid19alpha	10	180	-26.19724757	2044.64656	1.11E-130	-6.66413175	-5.73586825
shed7_covid19alpha	10	360	-32.54644056	1963.596381	3.36E-186	-10.23872876	-9.07493244
shed7_covid19alpha	30	60	-8.258785714	3159.062005	2.14E-16	-1.627565554	-1.003035539
shed7_covid19alpha	30	90	-12.61686429	2792.63578	1.56E-35	-2.706694986	-1.978550916
shed7_covid19alpha	30	180	-21.62096507	2346.800656	1.09E-94	-5.801559021	-4.836692345
shed7_covid19alpha	30	360	-28.84738098	2155.58589	5.04E-155	-9.372551924	-8.179360644
shed7_covid19alpha	60	90	-4.866977062	3511.182235	1.18E-06	-1.44117462	-0.6134701886
shed7_covid19alpha	60	180	-15.06812402	2931.100481	1.78E-49	-4.524831922	-3.482818351
shed7_covid19alpha	60	360	-23.28841377	2560.626064	5.70E-109	-8.088845007	-6.832466469
shed7_covid19alpha	90	180	-10.54230625	3305.062303	1.40E-25	-3.530079457	-2.422926008
shed7_covid19alpha	90	360	-19.24554398	2880.042727	9.56E-78	-7.088778715	-5.777887952
shed7_covid19alpha	180	360	-9.312551536	3470.521923	2.15E-20	-4.184625468	-2.729035735
shed7_covid19beta	5	10	-3.897135223	3506.874744	9.92E-05	-0.4147893204	-0.1371232479
shed7_covid19beta	5	30	-11.35699045	2819.334172	2.93E-29	-1.245061533	-0.8784357346
shed7_covid19beta	5	60	-17.23960195	2282.600313	1.05E-62	-2.558581846	-2.035953673
shed7_covid19beta	5	90	-20.47295091	2109.314525	4.10E-85	-3.737659401	-3.084198523
shed7_covid19beta	5	180	-27.70671004	1966.770531	6.23E-143	-6.936100877	-6.019090379
shed7_covid19beta	5	360	-33.63787313	1916.061149	2.46E-195	-10.4037587	-9.257443485
shed7_covid19beta	10	30	-7.945413948	3194.420605	2.66E-15	-0.9797040181	-0.5918806813
shed7_covid19beta	10	60	-14.74277607	2506.273311	3.08E-47	-2.290162594	-1.752460357
shed7_covid19beta	10	90	-18.473441	2252.87384	4.60E-71	-3.467760511	-2.802184844
shed7_covid19beta	10	180	-26.27733586	2038.561592	2.59E-131	-6.664479675	-5.738799014
shed7_covid19beta	10	360	-32.49620421	1961.577377	1.06E-185	-10.1312759	-8.978013714
shed7_covid19beta	30	60	-8.232425061	3152.743236	2.66E-16	-1.529782722	-0.9412555297
shed7_covid19beta	30	90	-13.02596191	2726.003908	1.13E-37	-2.702809035	-1.99555162
shed7_covid19beta	30	180	-22.21673474	2290.437442	3.43E-99	-5.893886519	-4.93780747
shed7_covid19beta	30	360	-29.20100001	2123.208009	7.08E-158	-9.3577514	-8.179953518
shed7_covid19beta	60	90	-5.464013786	3457.608285	4.98E-08	-1.513275896	-0.7140465085
shed7_covid19beta	60	180	-15.97894494	2834.128463	4.25E-55	-4.693302453	-3.667353285
shed7_covid19beta	60	360	-23.91924083	2496.657842	5.09E-114	-8.150920804	-6.915745863
shed7_covid19beta	90	180	-10.94937308	3273.075727	1.99E-27	-3.615810408	-2.517522926
shed7_covid19beta	90	360	-19.42739269	2860.59536	4.53E-79	-7.067605303	-5.771738959
shed7_covid19beta	180	360	-9.088965124	3480.319015	1.64E-19	-4.076306317	-2.629704612
shed7_covid19delta	5	10	-4.096590189	3551.221645	4.29E-05	-0.5356898761	-0.1889002879
shed7_covid19delta	5	30	-13.59145276	2593.098869	1.08E-40	-2.086578633	-1.560415903

shed7_covid19delta	5	60	-19.92852972	2210.916533	2.12E-81	-4.027479586	-3.305853747
shed7_covid19delta	5	90	-23.96119054	2082.601531	2.91E-112	-5.775160568	-4.901342164
shed7_covid19delta	5	180	-29.80680798	1987.18078	1.10E-161	-8.863576005	-7.76921088
shed7_covid19delta	5	360	-34.14575158	1954.24317	8.28E-201	-11.27642293	-10.05144592
shed7_covid19delta	10	30	-10.50260854	2865.567224	2.41E-25	-1.734002396	-1.188401975
shed7_covid19delta	10	60	-17.61014558	2365.152282	2.53E-65	-3.672327957	-2.936415212
shed7_covid19delta	10	90	-22.03596727	2187.244726	2.37E-97	-5.418782089	-4.53313048
shed7_covid19delta	10	180	-28.26317038	2053.119968	1.02E-148	-8.506015997	-7.402180725
shed7_covid19delta	10	360	-32.75871966	2006.577904	6.50E-189	-10.91836151	-9.684917183
shed7_covid19delta	30	60	-8.651586712	3260.289539	7.84E-18	-2.260883211	-1.425455587
shed7_covid19delta	30	90	-14.21110634	2886.114385	2.37E-44	-3.999704493	-3.029803704
shed7_covid19delta	30	180	-21.71772066	2525.704718	5.01E-96	-7.079142964	-5.906649386
shed7_covid19delta	30	360	-26.76826856	2388.127321	2.87E-138	-9.488059343	-8.192814974
shed7_covid19delta	60	90	-6.023122034	3510.613317	1.89E-09	-2.215717084	-1.127452315
shed7_covid19delta	60	180	-14.333403	3113.795504	3.59E-45	-5.285782345	-4.013671207
shed7_covid19delta	60	360	-19.79737782	2903.470833	6.29E-82	-7.690294501	-6.304241018
shed7_covid19delta	90	180	-8.561003181	3469.438719	1.66E-17	-3.660198621	-2.296085532
shed7_covid19delta	90	360	-14.19781474	3278.07871	1.91E-44	-6.061148528	-4.590217592
shed7_covid19delta	180	360	-5.711851914	3608.968632	1.21E-08	-3.153346081	-1.541735886
shed7_diphtheria	5	10	-5.828634535	3355.847882	6.12E-09	-0.6776864599	-0.3365211904
shed7_diphtheria	5	30	-14.60172582	2573.843463	1.87E-46	-2.034906362	-1.55307178
shed7_diphtheria	5	60	-21.60301102	2190.656501	5.48E-94	-4.025144399	-3.35518347
shed7_diphtheria	5	90	-26.1096258	2042.296463	6.48E-130	-6.14517132	-5.286522669
shed7_diphtheria	5	180	-36.81647366	1926.045344	4.00E-225	-12.41073824	-11.15538198
shed7_diphtheria	5	360	-48.43916702	1893.223835	0	-19.74539697	-18.20870139
shed7_diphtheria	10	30	-9.765335715	3079.675416	3.32E-22	-1.545272717	-1.028497774
shed7_diphtheria	10	60	-17.9488279	2484.876873	7.84E-68	-3.530811142	-2.835309076
shed7_diphtheria	10	90	-23.24867546	2221.892492	3.37E-107	-5.648102104	-4.769384235
shed7_diphtheria	10	180	-34.84777933	2008.941323	1.62E-208	-11.9105388	-10.64137377
shed7_diphtheria	10	360	-46.7997742	1948.199169	0.00E-02	-19.24394336	-17.69594735
shed7_diphtheria	30	60	-9.604693144	3235.534829	1.48E-21	-2.28325909	-1.509090637
shed7_diphtheria	30	90	-16.32385346	2763.90533	2.93E-57	-4.392951829	-3.450764018
shed7_diphtheria	30	180	-29.81773243	2278.275313	3.13E-165	-10.64601657	-9.332125506
shed7_diphtheria	30	360	-42.5238383	2128.79185	1.48E-286	-17.97549417	-16.39062604
shed7_diphtheria	60	90	-7.559510992	3424.586161	5.16E-14	-2.55107016	-1.50029596
shed7_diphtheria	60	180	-22.77102306	2737.355945	2.95E-105	-8.789781931	-7.396010419
shed7_diphtheria	60	360	-36.29822471	2453.07611	2.73E-231	-16.1127255	-14.46104499
shed7_diphtheria	90	180	-15.93010988	3205.418448	4.81E-55	-6.813975766	-5.320450463
shed7_diphtheria	90	360	-29.94552492	2836.255608	1.93E-171	-14.12953143	-12.39287294
shed7_diphtheria	180	360	-14.37024673	3512.249624	1.52E-45	-8.175518506	-6.212459636
shed7_fifth	5	10	-2.357487054	3623.073181	0.01845198538	-0.2222009285	-0.02042202229
shed7_fifth	5	30	-8.195740543	3142.956013	3.59E-16	-0.6433194171	-0.394931949
shed7_fifth	5	60	-12.87558013	2616.723415	7.92E-37	-1.193222264	-0.8778159869
shed7_fifth	5	90	-15.34768611	2336.628892	1.01E-50	-1.698434524	-1.313587333
shed7_fifth	5	180	-21.98187562	2038.051497	2.79E-96	-3.552747856	-2.970749412
shed7_fifth	5	360	-28.34880307	1909.881165	7.39E-148	-7.119111508	-6.197828382
shed7_fifth	10	30	-6.086419452	3319.52526	1.29E-09	-0.5259660582	-0.2696623571
shed7_fifth	10	60	-11.14541688	2766.18562	2.98E-28	-1.075044921	-0.7533703799
shed7_fifth	10	90	-13.92467051	2441.354939	1.81E-42	-1.579699201	-1.189699706
shed7_fifth	10	180	-21.04046435	2083.169733	2.84E-89	-3.433145624	-2.847728693
shed7_fifth	10	360	-27.76696146	1927.469573	5.01E-143	-6.99888151	-6.07543543
shed7_fifth	30	60	-5.74042652	3349.450145	1.03E-08	-0.6927703051	-0.3400165802
shed7_fifth	30	90	-9.303246516	2928.429553	2.59E-20	-1.194883531	-0.7788869606
shed7_fifth	30	180	-17.83716164	2315.138944	8.74E-67	-3.044142557	-2.441103344
shed7_fifth	30	360	-25.76212527	2019.476408	8.75E-127	-6.606701269	-5.671987255
shed7_fifth	60	90	-4.018361373	3473.382806	5.98E-05	-0.7000551385	-0.2409284681
shed7_fifth	60	180	-13.78056212	2699.644269	8.39E-42	-2.543000823	-1.909458193
shed7_fifth	60	360	-23.10096678	2183.076857	8.19E-106	-6.100285591	-5.145616049
shed7_fifth	90	180	-10.26440093	3101.859469	2.49E-24	-2.09112269	-1.42035272
shed7_fifth	90	360	-20.62504945	2387.035726	4.14E-87	-5.642336981	-4.662581052
shed7_fifth	180	360	-12.41991322	3057.456549	1.37E-34	-3.932964025	-2.860478598
shed7_flu	5	10	-3.631691687	3480.981471	2.86E-04	-0.3189132136	-0.09529443664
shed7_flu	5	30	-10.44082044	2874.606285	4.51E-25	-0.900261784	-0.6155852105
shed7_flu	5	60	-15.24076225	2380.805478	4.07E-50	-1.668944781	-1.288432268
shed7_flu	5	90	-18.44753328	2189.097588	9.52E-71	-2.408478414	-1.945619947
shed7_flu	5	180	-23.61757897	2004.574063	5.23E-109	-4.236866937	-3.587176779
shed7_flu	5	360	-29.6689597	1909.066822	2.10E-159	-7.667785657	-6.716913796
shed7_flu	10	30	-7.109270814	3286.728611	1.42E-12	-0.7027317962	-0.3989075481
shed7_flu	10	60	-12.62364063	2672.584213	1.58E-35	-1.469102158	-1.074067241
shed7_flu	10	90	-16.26974626	2390.530092	1.57E-56	-2.207378549	-1.732512161
shed7_flu	10	180	-22.07418933	2105.855108	2.65E-97	-4.034066333	-3.375769732
shed7_flu	10	360	-28.63651793	1955.601032	6.49E-151	-7.463631896	-6.506859907
shed7_flu	30	60	-6.535093434	3277.673634	7.34E-11	-0.9370121804	-0.5045178743
shed7_flu	30	90	-10.98875764	2881.54817	1.51E-27	-1.672348487	-1.165902879
shed7_flu	30	180	-18.153511	2376.544968	4.52E-69	-3.494807615	-2.813389106
shed7_flu	30	360	-25.94222763	2082.854176	8.19E-129	-6.920836901	-5.948015558
shed7_flu	60	90	-4.839743621	3493.806271	1.36E-06	-0.9812756579	-0.4154456535
shed7_flu	60	180	-13.13278283	2867.646484	2.66E-38	-2.796642434	-2.070024233
shed7_flu	60	360	-22.29756031	2337.435897	5.12E-100	-6.21615438	-5.211168024
shed7_flu	90	180	-8.802878079	3263.296432	2.12E-18	-2.1214083	-1.348537055
shed7_flu	90	360	-18.93229027	2597.485382	5.51E-75	-5.534751223	-4.49584987
shed7_flu	180	360	-11.33945671	3208.305271	2.99E-29	-3.847528844	-2.713126894
shed7_mers	5	10	-2.929321794	3567.423124	0.003418557241	-0.1860871998	-0.03686361991
shed7_mers	5	30	-8.938584741	2989.860925	6.75E-19	-0.5237246242	-0.3352917693
shed7_mers	5	60	-12.21798801	2601.934528	2.02E-33	-0.8212217165	-0.5940788299
shed7_mers	5	90	-15.12443954	2308.680871	2.39E-49	-1.228425012	-0.9464383758
shed7_mers	5	180	-19.43250943	2067.104793	2.17E-77	-2.130850065	-1.740188187
shed7_mers	5	360	-23.98424819	1927.259832	1.86E-111	-3.956441331	-3.358312768
shed7_mers	10	30	-6.310797145	3281.128814	3.15E-10	-0.4168416721	-0.2192239017
shed7_mers	10	60	-9.956546839	2851.739499	5.57E-23	-0.7135827785	-0.4787669482
shed7_mers	10	90	-13.28081254	2480.243306	6.18E-39	-1.120056886	-0.8318556819
shed7_mers	10	180	-18.10395942	2156.112604	2.63E-68	-2.02162856	-1.626458871
shed7_mers	10	360	-23.13869194	1964.420952	5.98E-105	-3.846442578	-3.245360701
shed7_mers	30	60	-4.171376631	3466.262301	3.10E-05	-0.4088756265	-0.1474085265
shed7_mers	30	90	-8.314875515	3026.269932	1.37E-16	-0.8130698132	-0.5027771813
shed7_mers	30	180	-14.35138837	2475.341364	6.30E-45	-1.711786921	-1.300234937
shed7_mers	30	360	-20.68767764	2102.106557	1.09E-86	-3.53385542	-2.921882285
shed7_mers	60	90	-4.442993175	3445.426811	9.15E-06	-0.5473755308	-0.2121873107
shed7_mers	60	180	-11.18199124	2821.373703	1.96E-28	-1.443180377	-1.012557328
shed7_mers	60	360	-18.51166006	2266.13595	2.33E-71	-3.262202682	-2.63725087
shed7_mers	90	180	-7.200479633	3273.982128	7.41E-13	-1.07902143	-0.6171534329
shed7_mers	90	360	-15.5810139	2537.017581	2.42E-52	-2.893377743	-2.246512968
shed7_mers	180	360	-9.632012808	3115.65573	1.17E-21	-2.072365249	-1.371350598
shed7_sars	5	10	-3.349819334	3558.13692	8.17E-04	-0.3439136216	-0.08996615986
shed7_sars	5	30	-10.7609824	2886.877184	1.66E-26	-1.081433777	-0.7480744196
shed7_sars	5	60	-15.98044336	2368.154013	1.14E-54	-2.071185919	-1.618431567
shed7_sars	5	90	-19.58214129	2184.7955	8.47E-79	-3.01006842	-2.462062728
shed7_sars	5	180	-25.40097804	1998.716918	1.37E-123	-5.423711436	-4.646233919
shed7_sars	5	360	-31.76483641	1915.727802	3.03E-178	-9.256875863	-8.180282607
shed7_sars	10	30	-7.836778128	3191.30835	6.25E-15	-0.8724024898	-0.5232259255
shed7_sars	10	60	-13.74357708	2569.253306	1.58E-41	-1.860127752	-1.395609953

shed7_sars	10	90	-17.71354606	2323.432007	5.89E-66	-2.798006546	-2.24024482
shed7_sars	10	180	-24.09207457	2066.530347	3.10E-113	-5.2102242	-4.425841373
shed7_sars	10	360	-30.83100602	1950.568532	2.27E-170	-9.042434311	-7.960844377
shed7_sars	30	60	-7.120336813	3243.32125	1.32E-12	-1.186159683	-0.6739496069
shed7_sars	30	90	-11.94282141	2858.850064	4.06E-32	-2.120337704	-1.522285247
shed7_sars	30	180	-19.8635239	2351.809836	2.70E-81	-4.526976123	-3.713461035
shed7_sars	30	360	-27.75248608	2099.917765	1.12E-144	-8.355272568	-7.252377705
shed7_sars	60	90	-5.201658196	3502.029545	2.09E-07	-1.227194942	-0.5553187192
shed7_sars	60	180	-14.39314575	2859.302142	2.12E-45	-3.624763459	-2.75556441
shed7_sars	60	360	-23.55321735	2390.760069	1.75E-110	-7.44605607	-6.301484914
shed7_sars	90	180	-9.774007229	3243.797508	2.94E-22	-2.760074861	-1.837739347
shed7_sars	90	360	-19.79205907	2663.923936	1.88E-81	-6.575218098	-5.389809224
shed7_sars	180	360	-11.04964062	3309.840728	6.73E-28	-4.337236621	-3.029976494
shed8_covid19	5	10	-5.878535101	4186.952601	4.46E-09	-1.068607381	-0.5340953217
shed8_covid19	5	30	-19.78120816	2941.799788	7.21E-82	-4.808910236	-3.941540214
shed8_covid19	5	60	-31.64064128	2512.731515	3.15E-185	-11.2731927	-9.957437929
shed8_covid19	5	90	-41.58133386	2413.22882	1.93E-285	-17.79510476	-16.19228263
shed8_covid19	5	180	-58.67122277	2354.759441	0	-29.46512593	-27.55919839
shed8_covid19	5	360	-70.08960001	2346.738968	0	-36.06675132	-34.10351895
shed8_covid19	10	30	-15.45535747	3357.247737	4.15E-52	-4.027256484	-3.120491264
shed8_covid19	10	60	-28.67815636	2699.595209	4.05E-158	-10.48498491	-9.142943013
shed8_covid19	10	90	-39.09162054	2538.141457	4.51E-262	-17.00457649	-15.3801082
shed8_covid19	10	180	-56.48070657	2442.445008	0	-28.67289366	-26.74872796
shed8_covid19	10	360	-67.87523156	2429.28296	0	-35.2742552	-33.29331236
shed8_covid19	30	60	-16.25076882	3741.131556	1.96E-57	-6.992935085	-5.487245095
shed8_covid19	30	90	-28.08180364	3306.676758	8.33E-156	-13.49949449	-11.73744245
shed8_covid19	30	180	-46.36144551	3004.330725	0	-25.15775484	-23.11611903
shed8_covid19	30	360	-57.47812056	2960.611482	0	-31.75752479	-29.66229503
shed8_covid19	60	90	-12.37763658	4258.834139	1.35E-34	-7.388663801	-5.368092956
shed8_covid19	60	180	-30.9354822	3905.342844	3.89E-188	-19.03108001	-16.76261368
shed8_covid19	60	360	-41.41475954	3838.091618	0.00E-02	-25.62822555	-23.31141409
shed8_covid19	90	180	-18.46435892	4302.205101	2.54E-73	-12.74148068	-10.29545625
shed8_covid19	90	360	-28.47837144	4255.593482	1.94E-163	-19.33690106	-16.84598183
shed8_covid19	180	360	-9.560190648	4433.877507	1.89E-21	-7.92088626	-5.225059686
shed8_covid19alpha	5	10	-6.410630653	4178.940238	1.61E-10	-1.348176194	-0.7166886711
shed8_covid19alpha	5	30	-22.37377232	2877.359494	2.34E-102	-6.61597352	-5.549792245
shed8_covid19alpha	5	60	-37.16310933	2511.90819	2.77E-241	-15.48560305	-13.93331587
shed8_covid19alpha	5	90	-47.13702685	2437.487915	0	-22.34327539	-20.55852641
shed8_covid19alpha	5	180	-63.25656773	2401.940976	0	-32.32138485	-30.37771425
shed8_covid19alpha	5	360	-72.06441766	2397.626076	0	-37.1110095	-35.14484635
shed8_covid19alpha	10	30	-17.81442336	3272.024158	7.90E-68	-5.606312618	-4.494588283
shed8_covid19alpha	10	60	-33.86378977	2702.3866	7.64E-210	-14.46897891	-12.88507515
shed8_covid19alpha	10	90	-44.18469683	2580.804144	0.00E-02	-21.32462474	-19.5123122
shed8_covid19alpha	10	180	-60.38465273	2522.328323	0	-31.30162199	-29.33261225
shed8_covid19alpha	10	360	-69.12261197	2515.215375	0	-36.09110347	-34.09988752
shed8_covid19alpha	30	60	-18.77557307	3846.922763	2.53E-75	-9.527380014	-7.725773139
shed8_covid19alpha	30	90	-30.05083382	3524.159481	7.86E-177	-16.37068937	-14.36534666
shed8_covid19alpha	30	180	-46.12656019	3343.268837	0	-26.34066159	-24.19267174
shed8_covid19alpha	30	360	-54.33472683	3320.213901	0	-31.12922609	-28.960864
shed8_covid19alpha	60	90	-11.48941403	4344.777988	4.04E-30	-7.891775502	-5.59110738
shed8_covid19alpha	60	180	-26.89496355	4210.651448	3.60E-147	-17.85308132	-15.42709886
shed8_covid19alpha	60	360	-34.36249049	4189.279324	3.42E-228	-22.6404858	-20.19645114
shed8_covid19alpha	90	180	-15.03896581	4403.190748	6.94E-50	-11.18905183	-8.608245469
shed8_covid19alpha	90	360	-22.15304135	4393.379009	3.59E-103	-15.97591738	-13.37813668
shed8_covid19alpha	180	360	-6.915288959	4437.363819	5.33E-12	-6.133058544	-3.423698213
shed8_covid19beta	5	10	-6.367475938	4172.502257	2.13E-10	-1.321448229	-0.6992724913
shed8_covid19beta	5	30	-22.00169321	2893.754929	2.49E-99	-6.333574422	-5.297056208
shed8_covid19beta	5	60	-36.35480974	2510.57431	7.27E-233	-14.94170892	-13.41234513
shed8_covid19beta	5	90	-46.62414371	2436.233506	0	-21.79638879	-20.03694454
shed8_covid19beta	5	180	-62.7480136	2395.607138	0	-32.06773356	-30.12415833
shed8_covid19beta	5	360	-72.05530392	2391.39648	0	-37.10974701	-35.14340614
shed8_covid19beta	10	30	-17.40514014	3302.703605	5.51E-65	-5.346231333	-4.263678577
shed8_covid19beta	10	60	-33.0801788	2703.506924	8.51E-202	-13.94712651	-12.38620683
shed8_covid19beta	10	90	-43.68804345	2581.229417	0.00E-02	-20.79977609	-19.01283652
shed8_covid19beta	10	180	-59.93913259	2513.918549	0	-31.0698352	-29.10133597
shed8_covid19beta	10	360	-69.17173704	2506.925895	0	-36.11170611	-34.12072633
shed8_covid19beta	30	60	-18.55387257	3813.253341	1.21E-73	-9.245293147	-7.478130277
shed8_covid19beta	30	90	-30.06490679	3491.03724	8.41E-177	-16.08616632	-14.11653639
shed8_covid19beta	30	180	-46.41794119	3285.0461	0	-26.34848041	-24.21278085
shed8_covid19beta	30	360	-55.11967427	3262.583556	0	-31.38947932	-29.2330432
shed8_covid19beta	60	90	-11.65290083	4344.056477	6.37E-31	-7.873531762	-5.605747517
shed8_covid19beta	60	180	-27.48836615	4183.090853	4.63E-153	-18.12561375	-15.71222409
shed8_covid19beta	60	360	-35.39241184	4160.64866	4.28E-240	-23.16542727	-20.73367183
shed8_covid19beta	90	180	-15.55904223	4390.9102	3.55E-53	-11.46191105	-8.896647513
shed8_covid19beta	90	360	-23.09279425	4379.559307	1.94E-111	-16.50118355	-13.91863626
shed8_covid19beta	180	360	-7.275227119	4437.350084	4.07E-13	-6.386264601	-3.67499666
shed8_covid19delta	5	10	-8.971079672	4009.362208	4.42E-19	-2.387134352	-1.530883666
shed8_covid19delta	5	30	-29.69506019	2775.80815	1.61E-168	-11.92467039	-10.44740168
shed8_covid19delta	5	60	-45.15624042	2565.022903	0	-22.13000061	-20.28801741
shed8_covid19delta	5	90	-53.8113978	2523.957825	0	-27.80041058	-25.84553537
shed8_covid19delta	5	180	-65.65680044	2510.637863	0	-34.42396859	-32.42738276
shed8_covid19delta	5	360	-71.58825459	2512.89614	0	-37.30726893	-35.3179563
shed8_covid19delta	10	30	-23.25050294	3268.353649	8.63E-111	-10.00513236	-8.448921693
shed8_covid19delta	10	60	-39.60943454	2889.391436	1.87E-274	-20.20293251	-18.29706749
shed8_covid19delta	10	90	-48.38623357	2812.140365	0	-25.87155339	-23.85637453
shed8_covid19delta	10	180	-60.02954569	2786.891759	0	-32.49449937	-30.43883397
shed8_covid19delta	10	360	-65.76292762	2791.178681	0	-35.37790466	-33.32930255
shed8_covid19delta	30	60	-17.43701215	4199.732093	8.54E-66	-11.1499051	-8.896040842
shed8_covid19delta	30	90	-26.12433574	4077.280191	3.10E-139	-16.81043793	-14.46343595
shed8_covid19delta	30	180	-36.61191308	4030.7967	1.30E-253	-23.43056308	-21.0487162
shed8_covid19delta	30	360	-41.47056348	4038.918157	0.00E-02	-26.31445404	-23.93869912
shed8_covid19delta	60	90	-8.492648712	4420.026073	2.72E-17	-6.90992962	-4.317998308
shed8_covid19delta	60	180	-18.25853745	4405.284922	7.45E-72	-13.52842602	-10.90490732
shed8_covid19delta	60	360	-22.62090093	4408.133052	2.79E-107	-16.41259875	-13.79460846
shed8_covid19delta	90	180	-9.574654799	4435.748206	1.65E-21	-7.954667018	-5.250738387
shed8_covid19delta	90	360	-13.78838419	4436.458246	2.22E-42	-10.83892229	-8.140356986
shed8_covid19delta	180	360	-4.148047002	4437.932817	3.42E-05	-4.251394965	-1.522478909
shed8_diphtheria	5	10	-7.593615792	4052.282092	3.84E-14	-1.691747312	-0.9974418771
shed8_diphtheria	5	30	-30.02724084	2705.161892	3.24E-171	-10.54121208	-9.248878012
shed8_diphtheria	5	60	-45.34164216	2460.529112	0	-21.64739291	-19.85260709
shed8_diphtheria	5	90	-59.02420457	2425.099112	0	-30.10959843	-28.17328445
shed8_diphtheria	5	180	-80.17794388	2413.18779	0	-41.73002799	-39.73753958
shed8_diphtheria	5	360	-100.6217546	2439.218413	0	-49.06401776	-47.18823449
shed8_diphtheria	10	30	-24.86321825	3111.021663	1.28E-124	-9.224743589	-7.876157312
shed8_diphtheria	10	60	-41.4569172	2672.580359	1.62E-290	-20.32325292	-18.48755789
shed8_diphtheria	10	90	-55.21589226	2606.848524	0	-28.78399246	-26.80970123
shed8_diphtheria	10	180	-76.11808507	2584.661281	0	-40.40389629	-38.37448209
shed8_diphtheria	10	360	-95.80585425	2633.092664	0	-47.73901252	-45.82405055
shed8_diphtheria	30	60	-19.94019278	3977.470317	2.14E-84	-11.92223636	-9.787673553
shed8_diphtheria	30	90	-33.46955974	3802.637527	1.72E-215	-20.3738165	-18.1189763
shed8_diphtheria	30	180	-52.50180315	3736.354537	0	-31.99036401	-29.68711346

shed8_diphtheria	30	360	-68.04532357	3876.30313	0	-39.3326255	-37.12953666
shed8_diphtheria	60	90	-12.77440754	4410.08948	1.01E-36	-9.679284974	-7.103597909
shed8_diphtheria	60	180	-29.92811876	4385.72781	3.03E-179	-21.29286402	-18.67470355
shed8_diphtheria	60	360	-42.41888633	4428.464218	0	-28.64138542	-26.11086683
shed8_diphtheria	90	180	-16.72885862	4434.041585	5.77E-61	-12.95087957	-10.23380511
shed8_diphtheria	90	360	-28.27437345	4433.092181	7.64E-162	-20.30105197	-17.6683174
shed8_diphtheria	180	360	-10.83849389	4420.405896	4.93E-27	-8.729492427	-6.055192258
shed8_fifth	5	10	-4.133567172	4298.228387	3.64E-05	-0.5711219588	-0.203652816
shed8_fifth	5	30	-15.21739548	3207.544752	1.52E-50	-2.362441228	-1.823144357
shed8_fifth	5	60	-25.26081787	2579.837271	5.40E-126	-5.937619494	-5.082200325
shed8_fifth	5	90	-32.5679636	2409.903501	3.99E-193	-10.19187497	-9.034251159
shed8_fifth	5	180	-51.16105072	2307.145563	0	-22.83390123	-21.14808075
shed8_fifth	5	360	-69.07679418	2290.11736	0	-33.93265796	-32.05923393
shed8_fifth	10	30	-11.91184803	3566.748939	4.14E-32	-1.98610642	-1.424704391
shed8_fifth	10	60	-23.10339002	2734.920339	4.96E-108	-5.557281441	-4.687763604
shed8_fifth	10	90	-30.97526193	2493.355456	1.63E-178	-9.809715092	-8.641636259
shed8_fifth	10	180	-50.04576664	2345.876061	0	-22.45011162	-20.75709558
shed8_fifth	10	360	-68.03009654	2321.380932	0	-33.54850932	-31.6686078
shed8_fifth	30	60	-14.03347843	3597.289787	1.35E-43	-3.894524106	-2.939710128
shed8_fifth	30	90	-23.92041348	3006.220909	6.63E-116	-8.13670592	-6.90383462
shed8_fifth	30	180	-44.89102588	2591.490678	0	-20.76736914	-19.02902726
shed8_fifth	30	360	-63.09611976	2520.236868	0	-31.86356355	-29.94274275
shed8_fifth	60	90	-11.49101642	4048.496988	4.27E-30	-4.803216326	-3.403089981
shed8_fifth	60	180	-34.73446476	3236.376008	5.77E-225	-17.41140808	-15.55075408
shed8_fifth	60	360	-53.03984563	3055.989372	0	-28.50212092	-26.46995115
shed8_fifth	90	180	-24.05922831	3906.455078	2.18E-119	-13.38659752	-11.36925834
shed8_fifth	90	360	-42.12654298	3669.054502	0.00E-02	-24.47114526	-22.29462051
shed8_fifth	180	360	-17.27703093	4387.776609	9.32E-65	-12.25373821	-9.756171704
shed8_flu	5	10	-5.793167676	4072.74997	7.43E-09	-0.7409563162	-0.366250891
shed8_flu	5	30	-18.45437846	2845.045434	6.43E-72	-3.254471165	-2.629312619
shed8_flu	5	60	-27.20578661	2467.002419	9.23E-143	-7.159739433	-6.197017324
shed8_flu	5	90	-32.62727864	2364.844202	4.07E-193	-10.96107359	-9.71820569
shed8_flu	5	180	-45.87597282	2287.710631	0	-21.90846334	-20.11225738
shed8_flu	5	360	-57.31874507	2272.865445	0	-30.61361692	-28.58818488
shed8_flu	10	30	-14.23526507	3325.441674	1.11E-44	-2.717236386	-2.05934019
shed8_flu	10	60	-24.40296411	2675.547457	6.36E-119	-6.616918715	-5.632630834
shed8_flu	10	90	-30.46829457	2488.825357	1.48E-173	-10.4158583	-9.156213768
shed8_flu	10	180	-44.37880845	2346.381983	0.00E-02	-21.36068367	-19.55282984
shed8_flu	10	360	-55.96057252	2318.883154	0	-30.06518159	-28.029413
shed8_flu	30	60	-13.27080056	3719.372442	2.64E-39	-4.288507659	-3.184465314
shed8_flu	30	90	-21.40525213	3182.672329	4.44E-95	-8.07537748	-6.720118015
shed8_flu	30	180	-37.77708599	2690.848672	6.20E-251	-19.00632492	-17.13061202
shed8_flu	30	360	-49.87466668	2590.507637	0	-27.70713876	-25.61087926
shed8_flu	60	90	-9.321043161	4156.290238	1.82E-20	-4.431350017	-2.891172505
shed8_flu	60	180	-27.91427514	3358.086806	2.18E-154	-15.33864601	-13.32531795
shed8_flu	60	360	-40.48643094	3136.99556	1.08E-288	-24.03263914	-21.81240591
shed8_flu	90	180	-19.36049623	3929.145721	7.81E-80	-11.75130629	-9.590135152
shed8_flu	90	360	-32.06980643	3660.336444	4.27E-199	-20.43881329	-18.08370923
shed8_flu	180	360	-12.529977	4373.79849	2.06E-35	-9.934661978	-7.246419103
shed8_mers	5	10	-5.918654082	3831.516205	3.53E-09	-0.3933798342	-0.1976111568
shed8_mers	5	30	-14.1931316	2851.873481	3.13E-44	-1.241199871	-0.93988121
shed8_mers	5	60	-18.60563741	2502.342631	1.79E-72	-2.295434162	-1.857718991
shed8_mers	5	90	-22.59726775	2359.250885	1.64E-102	-3.841910895	-3.228359375
shed8_mers	5	180	-32.7009461	2257.089384	2.45E-192	-10.29315027	-9.128471354
shed8_mers	5	360	-45.2036572	2236.537537	0.00E-02	-20.5941048	-18.88157088
shed8_mers	10	30	-9.57438269	3565.459016	1.85E-21	-0.9578533287	-0.6322367614
shed8_mers	10	60	-15.35842335	2865.159695	3.23E-51	-2.008469661	-1.553692502
shed8_mers	10	90	-20.30117598	2543.163918	4.83E-85	-3.552557491	-2.926721788
shed8_mers	10	180	-31.52908241	2307.39426	9.57E-182	-10.0009132	-8.829717431
shed8_mers	10	360	-44.41155908	2259.709837	0.00E-02	-20.30082858	-18.5838561
shed8_mers	30	60	-7.59396831	3855.525297	3.87E-14	-1.240606759	-0.7314653128
shed8_mers	30	90	-14.3858056	3139.603003	1.72E-45	-2.777781718	-2.111407471
shed8_mers	30	180	-28.32996467	2479.66717	3.35E-153	-9.216941334	-8.023599207
shed8_mers	30	360	-42.22137283	2339.340599	4.42E-290	-19.51337407	-17.78122052
shed8_mers	60	90	-7.748910064	3980.121913	1.17E-14	-1.827590051	-1.089527066
shed8_mers	60	180	-24.24605119	2802.732534	4.69E-118	-8.251625021	-7.016843448
shed8_mers	60	360	-39.33352975	2491.535124	1.22E-263	-18.54173805	-16.78078447
shed8_mers	90	180	-18.52320866	3340.586881	5.39E-73	-6.829368524	-5.521982827
shed8_mers	90	360	-35.05555668	2764.880727	4.48E-223	-17.10899616	-15.29640925
shed8_mers	180	360	-19.03996015	3904.908862	2.31E-77	-11.05952421	-8.994529844
shed8_sars	5	10	-6.654091265	4038.980078	3.23E-11	-1.033961292	-0.5633360049
shed8_sars	5	30	-21.97746781	2807.804276	6.51E-99	-4.876463212	-4.077590842
shed8_sars	5	60	-31.84935404	2448.548655	1.50E-186	-10.70080693	-9.459553432
shed8_sars	5	90	-41.26841875	2361.970121	8.66E-281	-17.18825095	-15.62886616
shed8_sars	5	180	-57.28440378	2311.015275	0	-29.20775203	-27.27422995
shed8_sars	5	360	-68.76408328	2303.258779	0	-36.40674731	-34.38784729
shed8_sars	10	30	-17.14575818	3295.990069	3.44E-63	-4.099015221	-3.257741535
shed8_sars	10	60	-28.68479883	2655.860587	7.19E-158	-9.916005624	-8.647057439
shed8_sars	10	90	-38.70934743	2492.283072	4.37E-257	-16.40066792	-14.8191519
shed8_sars	10	180	-55.15303962	2395.170436	0	-28.41804915	-26.46663554
shed8_sars	10	360	-66.64560764	2380.349958	0	-35.61666928	-33.58062801
shed8_sars	30	60	-15.43449084	3703.898057	3.90E-52	-6.314907648	-4.891398658
shed8_sars	30	90	-27.3937099	3221.283421	1.06E-148	-12.78552912	-11.07753394
shed8_sars	30	180	-45.34371828	2884.715961	0	-24.79158291	-22.73634502
shed8_sars	30	360	-56.77486113	2830.833781	0	-31.98814675	-29.8523938
shed8_sars	60	90	-12.69791517	4209.113504	2.79E-36	-7.305464872	-5.351291885
shed8_sars	60	180	-31.45528019	3749.205596	6.03E-193	-19.29276798	-17.02885364
shed8_sars	60	360	-42.47491848	3652.054199	0.00E-02	-26.48573827	-24.14849596
shed8_sars	90	180	-18.91657832	4238.183647	1.11E-76	-13.0587518	-10.60611307
shed8_sars	90	360	-29.54055783	4159.622097	2.83E-174	-20.24897481	-17.72850267
shed8_sars	180	360	-10.13932336	4429.410167	6.73E-24	-8.540021558	-5.772591054
shed9_covid19	5	10	-6.364168599	3561.942713	2.21E-10	-0.3868319234	-0.2046210681
shed9_covid19	5	30	-14.6919647	2469.258496	6.54E-47	-2.204936718	-1.685661572
shed9_covid19	5	60	-21.06703779	2375.584349	1.81E-90	-5.698974797	-4.728375631
shed9_covid19	5	90	-24.53980896	2360.105029	1.23E-118	-8.62081789	-7.344994076
shed9_covid19	5	180	-27.67236629	2352.195103	3.72E-146	-12.18130619	-10.56912116
shed9_covid19	5	360	-31.04688878	2350.38449	1.21E-177	-14.60484289	-12.86951608
shed9_covid19	10	30	-12.05002249	2796.399043	1.23E-32	-1.917995631	-1.381149668
shed9_covid19	10	60	-19.67886469	2468.524954	3.35E-80	-5.408003962	-4.427893474
shed9_covid19	10	90	-23.49693023	2413.756459	4.08E-110	-8.32871664	-7.045642334
shed9_covid19	10	180	-26.85707622	2385.745279	4.77E-139	-11.88845157	-10.27052279
shed9_covid19	10	360	-30.28518796	2379.331906	1.08E-170	-14.31178512	-12.57112087
shed9_covid19	30	60	-11.71565294	3556.055324	3.93E-31	-3.815343489	-2.721408648
shed9_covid19	30	90	-17.25709823	3077.031101	9.05E-64	-6.723594039	-5.351619636
shed9_covid19	30	180	-21.89116023	2807.71217	3.29E-98	-10.27455974	-8.585269317
shed9_covid19	30	360	-25.58882388	2744.446402	1.06E-129	-12.6954724	-10.88828829
shed9_covid19	60	90	-6.794379141	4363.778408	1.23E-11	-3.568288093	-1.970173445
shed9_covid19	60	180	-12.86806403	3831.959048	3.96E-37	-7.100312864	-5.222764059
shed9_covid19	60	360	-16.84369703	3666.140934	2.24E-61	-9.515642669	-7.531365878
shed9_covid19	90	180	-6.482458276	4441.771888	1.00E-10	-4.418247679	-2.366367706
shed9_covid19	90	360	-10.49448819	4293.63947	1.86E-25	-6.829251986	-4.679295022

shed9_covid19	180	360	-3.915986552	4652.75218	9.13E-05	-3.544444978	-1.179486645
shed9_covid19alpha	5	10	-7.314002413	3244.085247	3.25E-13	-0.5950194431	-0.3434420954
shed9_covid19alpha	5	30	-16.6187675	2448.843566	7.55E-59	-3.220685894	-2.540852568
shed9_covid19alpha	5	60	-23.12747913	2375.344526	6.74E-107	-7.504053289	-6.330989446
shed9_covid19alpha	5	90	-26.55417525	2362.83694	3.48E-136	-10.51774301	-9.071145879
shed9_covid19alpha	5	180	-28.55174221	2356.762819	3.05E-154	-13.02923466	-11.35452602
shed9_covid19alpha	5	360	-31.17877424	2355.246622	5.81E-179	-14.79421884	-13.04338799
shed9_covid19alpha	10	30	-13.31861025	2877.587267	2.57E-39	-2.766569304	-2.056507619
shed9_covid19alpha	10	60	-21.23636123	2519.274926	3.33E-92	-7.04370768	-5.852873517
shed9_covid19alpha	10	90	-25.03154103	2457.331432	2.29E-123	-10.05573561	-8.594691742
shed9_covid19alpha	10	180	-27.24916038	2427.202355	7.07E-143	-12.56625112	-10.87904802
shed9_covid19alpha	10	360	-29.92294419	2419.678087	1.17E-167	-14.33096555	-12.56817975
shed9_covid19alpha	30	60	-11.74487399	3733.550979	2.65E-31	-4.710616714	-3.36288756
shed9_covid19alpha	30	90	-17.03488921	3308.043347	1.93E-62	-7.709425276	-6.117925151
shed9_covid19alpha	30	180	-20.2700005	3075.826215	7.55E-86	-10.21178334	-8.410438882
shed9_covid19alpha	30	360	-23.11870405	3015.746336	5.29E-109	-11.97419547	-10.1018729
shed9_covid19alpha	60	90	-6.076815424	4484.276342	1.33E-09	-3.805071717	-1.948774437
shed9_covid19alpha	60	180	-10.14259898	4184.432464	6.74E-24	-6.293875242	-4.254842706
shed9_covid19alpha	60	360	-13.06032486	4081.820553	3.21E-38	-8.052276647	-5.950287455
shed9_covid19alpha	90	180	-4.258070217	4580.340043	2.10E-05	-3.501252835	-1.293618959
shed9_covid19alpha	90	360	-7.1369025	4516.051523	1.11E-12	-5.257310158	-2.991407791
shed9_covid19alpha	180	360	-2.800524789	4668.720324	0.005122942114	-2.935833905	-0.5180122484
shed9_covid19beta	5	10	-7.275075383	3265.390962	4.31E-13	-0.5837566525	-0.3359014671
shed9_covid19beta	5	30	-16.30261954	2455.765012	8.17E-57	-3.059234759	-2.402303702
shed9_covid19beta	5	60	-22.87032609	2374.874446	8.61E-105	-7.442441378	-6.266960332
shed9_covid19beta	5	90	-26.505527	2363.056247	9.40E-136	-10.40616457	-8.972467906
shed9_covid19beta	5	180	-28.43884585	2356.788545	3.35E-153	-12.91469975	-11.24854811
shed9_covid19beta	5	360	-31.1707193	2355.094397	6.97E-179	-14.79489933	-13.04356221
shed9_covid19beta	10	30	-12.96399881	2896.711899	2.11E-37	-2.614416098	-1.927464244
shed9_covid19beta	10	60	-21.03077181	2512.51902	1.40E-90	-6.991129473	-5.798614116
shed9_covid19beta	10	90	-25.00302598	2455.440785	4.13E-123	-9.953334912	-8.505639447
shed9_covid19beta	10	180	-27.15946335	2425.126049	4.76E-142	-12.46090105	-10.78268869
shed9_covid19beta	10	360	-29.94433219	2416.928155	7.79E-168	-14.34080924	-12.57799418
shed9_covid19beta	30	60	-12.08108873	3651.671184	5.61E-33	-4.793195593	-3.454667654
shed9_covid19beta	30	90	-17.37881294	3263.025202	9.04E-65	-7.743615723	-6.173478294
shed9_covid19beta	30	180	-20.54415991	3035.099043	6.37E-88	-10.24330541	-8.458403993
shed9_covid19beta	30	360	-23.53011585	2971.401354	2.08E-112	-12.12079514	-10.25612793
shed9_covid19beta	60	90	-6.014812028	4502.86944	1.94E-09	-3.758540822	-1.910689947
shed9_covid19beta	60	180	-10.07894996	4200.787383	1.27E-23	-6.243649383	-4.21019677
shed9_covid19beta	60	360	-13.16694141	4086.010581	8.30E-39	-8.11643153	-6.012628299
shed9_covid19beta	90	180	-4.277816923	4575.342345	1.93E-05	-3.488679443	-1.295935942
shed9_covid19beta	90	360	-7.344765558	4500.996337	2.43E-13	-5.358978495	-3.100850565
shed9_covid19beta	180	360	-2.986825998	4666.333547	0.002833559064	-3.043762691	-0.6314509844
shed9_covid19delta	5	10	-8.510782865	3373.854395	2.56E-17	-1.186207212	-0.7419979158
shed9_covid19delta	5	30	-19.98488287	2501.520425	1.48E-82	-5.896997198	-4.843173742
shed9_covid19delta	5	60	-25.64051898	2422.908357	1.72E-128	-10.24218509	-8.786874743
shed9_covid19delta	5	90	-28.13855766	2404.593098	7.78E-151	-12.60907466	-10.96613901
shed9_covid19delta	5	180	-28.54262672	2398.604404	1.62E-154	-13.39668984	-11.67425033
shed9_covid19delta	5	360	-30.6476424	2396.086753	2.51E-174	-14.62977811	-12.87022189
shed9_covid19delta	10	30	-15.55982251	3021.423318	1.42E-52	-4.961196729	-3.850769083
shed9_covid19delta	10	60	-22.40250405	2696.730099	4.40E-102	-9.298828271	-7.80202643
shed9_covid19delta	10	90	-25.26909653	2619.252242	2.93E-126	-11.66340316	-9.983605392
shed9_covid19delta	10	180	-25.81884979	2593.816294	5.13E-131	-12.45018481	-10.69255024
shed9_covid19delta	10	360	-27.9501933	2583.109836	2.00E-150	-13.68290898	-11.88888589
shed9_covid19delta	30	60	-9.152532523	4244.937064	8.44E-20	-5.032207322	-3.256681567
shed9_covid19delta	30	90	-13.02294978	3961.386411	5.40E-38	-7.383658475	-5.45138426
shed9_covid19delta	30	180	-14.04620312	3850.043646	9.60E-44	-8.16553477	-6.165234461
shed9_covid19delta	30	360	-16.16809102	3800.505406	6.45E-57	-9.396086632	-7.363742427
shed9_covid19delta	60	90	-4.093608899	4608.760085	4.32E-05	-3.361680973	-1.184472873
shed9_covid19delta	60	180	-5.293182921	4547.461375	1.26E-07	-4.139834108	-1.902046234
shed9_covid19delta	60	360	-7.327334711	4514.487747	2.77E-13	-5.368705483	-3.102234688
shed9_covid19delta	90	180	-1.240395038	4667.313134	0.214891666	-1.929878011	0.4341515153
shed9_covid19delta	90	360	-3.217821282	4655.601822	0.00130053531	-3.157990985	-0.7667953397
shed9_covid19delta	180	360	-1.946518203	4675.822073	0.05165233656	-2.437765964	0.008706135148
shed9_diphtheria	5	10	-10.79854709	3370.058001	9.53E-27	-1.679947002	-1.163642742
shed9_diphtheria	5	30	-28.49392116	2475.74109	1.07E-154	-10.3264357	-8.996641219
shed9_diphtheria	5	60	-42.47879994	2400.695165	1.51E-294	-22.26494359	-20.30001367
shed9_diphtheria	5	90	-50.88337731	2387.794601	0	-29.73104317	-27.52451239
shed9_diphtheria	5	180	-63.33668653	2382.386235	0	-38.93815922	-36.59944762
shed9_diphtheria	5	360	-73.311987	2383.457144	0	-44.34762188	-42.03699351
shed9_diphtheria	10	30	-23.23398645	2918.946768	1.04E-109	-8.935117154	-7.544370025
shed9_diphtheria	10	60	-38.81476061	2603.777074	1.90E-260	-20.86402186	-18.85734567
shed9_diphtheria	10	90	-47.55185562	2548.650473	0	-28.32787565	-26.08409016
shed9_diphtheria	10	180	-60.0473596	2525.487288	0	-37.53395475	-35.16006234
shed9_diphtheria	10	360	-69.82083624	2530.075999	0	-42.94362802	-40.59739762
shed9_diphtheria	30	60	-19.38304019	4091.857685	3.81E-80	-12.79636793	-10.44551241
shed9_diphtheria	30	90	-29.09386608	3818.889007	2.89E-168	-20.24434142	-17.68813722
shed9_diphtheria	30	180	-41.26154471	3686.666368	2.65E-306	-29.44282618	-26.77170373
shed9_diphtheria	30	360	-49.67975915	3713.661098	0	-34.85405516	-32.2074833
shed9_diphtheria	60	90	-9.806681416	4615.051001	1.75E-22	-8.813716222	-5.876882069
shed9_diphtheria	60	180	-21.28243508	4540.103725	6.93E-96	-18.0050053	-14.96764427
shed9_diphtheria	60	360	-28.4859108	4557.750234	2.09E-164	-23.41772828	-20.40192984
shed9_diphtheria	90	180	-11.20442984	4661.945893	9.02E-29	-10.74045854	-7.541592741
shed9_diphtheria	90	360	-17.96711961	4667.897009	7.57E-70	-16.15373054	-12.97532928
shed9_diphtheria	180	360	-6.500154083	4677.304599	8.86E-11	-7.05925388	-3.787754667
shed9_fifth	5	10	-4.232071333	4175.573197	2.37E-05	-0.1732144878	-0.06353764893
shed9_fifth	5	30	-11.8049233	2636.240153	2.24E-31	-0.8984993404	-0.6425263006
shed9_fifth	5	60	-16.79159807	2387.670855	6.71E-60	-2.953745469	-2.335998121
shed9_fifth	5	90	-20.22761474	2360.536717	4.95E-84	-5.239085116	-4.313051636
shed9_fifth	5	180	-27.3459653	2347.111661	3.66E-143	-11.25936542	-9.752600391
shed9_fifth	5	360	-31.52392328	2344.378641	3.77E-182	-15.79373184	-13.943875
shed9_fifth	10	30	-9.687496914	2943.997441	7.20E-22	-0.7841306265	-0.5201428777
shed9_fifth	10	60	-15.9530421	2439.334726	1.42E-54	-2.837050844	-2.215940609
shed9_fifth	10	90	-19.67842925	2383.410407	5.28E-80	-5.121832372	-4.193552244
shed9_fifth	10	180	-27.01302172	2355.727874	3.12E-140	-11.14168024	-9.633533438
shed9_fifth	10	360	-31.25389192	2350.091898	1.27E-179	-15.6759186	-13.8249361
shed9_fifth	30	60	-11.0912407	3082.195687	4.66E-28	-2.205712281	-1.543005668
shed9_fifth	30	90	-16.42116027	2674.831625	8.28E-58	-4.483859071	-3.52725204
shed9_fifth	30	180	-25.02456621	2466.051279	2.37E-123	-10.49834207	-8.9725981
shed9_fifth	30	360	-29.64160112	2423.279257	5.01E-165	-15.03096458	-13.16561662
shed9_fifth	60	90	-7.532573797	4069.953463	6.09E-14	-2.685895578	-1.576497585
shed9_fifth	60	180	-18.96038484	3097.505982	6.17E-76	-8.674043847	-7.048178375
shed9_fifth	60	360	-24.60739595	2849.677487	2.11E-121	-13.19797398	-11.24988926
shed9_fifth	90	180	-12.72246547	3882.020323	2.34E-36	-6.612913876	-4.846915184
shed9_fifth	90	360	-19.15195786	3438.687325	9.11E-78	-11.12596426	-9.05950583
shed9_fifth	180	360	-7.176708914	4493.567585	8.31E-13	-5.554630719	-3.171010306
shed9_flu	5	10	-4.482869271	4169.66132	7.56E-06	-0.1541760949	-0.06035381963
shed9_flu	5	30	-11.84863531	2670.835422	1.33E-31	-0.7306737376	-0.5231724163
shed9_flu	5	60	-16.64704542	2385.522486	5.92E-59	-2.559466716	-2.020020464
shed9_flu	5	90	-21.00708556	2358.38021	5.93E-90	-4.880817814	-4.047387314
shed9_flu	5	180	-26.76332248	2347.432636	6.11E-138	-9.242973055	-7.980958569

shed9_flu	5	360	-31.21404512	2344.889489	3.52E-179	-12.77023337	-11.26053586
shed9_flu	10	30	-9.488436367	3015.598704	4.60E-21	-0.6270435853	-0.412272654
shed9_flu	10	60	-15.78402894	2435.407735	1.64E-53	-2.453620207	-1.911337058
shed9_flu	10	90	-20.4570902	2379.172095	8.21E-86	-4.774472328	-3.939202885
shed9_flu	10	180	-26.40449949	2356.480315	8.20E-135	-9.136315682	-7.873086027
shed9_flu	10	360	-30.91454106	2351.208601	2.19E-176	-12.66347638	-11.15276293
shed9_flu	30	60	-11.38117651	2979.133883	2.08E-29	-1.949292912	-1.376348113
shed9_flu	30	90	-17.59042667	2609.917747	1.52E-65	-4.264924823	-3.409434151
shed9_flu	30	180	-24.5292369	2457.201424	4.92E-119	-8.623387459	-7.346698011
shed9_flu	30	360	-29.34604758	2421.58067	3.19E-162	-12.14945453	-10.62746855
shed9_flu	60	90	-8.614810648	3999.481836	9.93E-18	-2.669199261	-1.679518687
shed9_flu	60	180	-18.09377255	3159.885146	1.05E-69	-7.007324016	-5.637120429
shed9_flu	60	360	-23.81893797	2921.812808	9.42E-115	-10.52625631	-8.925025742
shed9_flu	90	180	-10.76987552	4050.433902	1.09E-26	-4.902940928	-3.392785568
shed9_flu	90	360	-17.19041154	3640.402994	9.65E-64	-8.412527475	-6.690036628
shed9_flu	180	360	-6.788582104	4535.008578	1.28E-11	-4.386298435	-2.420539171
shed9_mers	5	10	-3.626187328	4402.116743	2.91E-04	-0.08493334288	-0.02532306738
shed9_mers	5	30	-10.99826567	2950.644644	1.33E-27	-0.3519733164	-0.245462581
shed9_mers	5	60	-14.2619329	2495.783082	2.00E-44	-0.8380503471	-0.6354539264
shed9_mers	5	90	-16.12892941	2377.241877	1.28E-55	-1.869780047	-1.464407987
shed9_mers	5	180	-21.57655227	2349.339176	2.38E-94	-4.665162921	-3.887828532
shed9_mers	5	360	-27.76371483	2343.457772	6.46E-147	-8.966762744	-7.783664606
shed9_mers	10	30	-8.636247512	3328.475602	8.86E-18	-0.2988916322	-0.188287855
shed9_mers	10	60	-13.05401207	2599.972378	9.04E-38	-0.7840124205	-0.5792354427
shed9_mers	10	90	-15.55350962	2402.775248	4.76E-52	-1.815198864	-1.40873276
shed9_mers	10	180	-21.28274176	2356.24435	4.44E-92	-4.610320253	-3.83241479
shed9_mers	10	360	-27.57220136	2346.435015	3.34E-145	-8.911822184	-7.728348756
shed9_mers	30	60	-7.703753748	3448.061499	1.72E-14	-0.5495165553	-0.3265518208
shed9_mers	30	90	-12.90242576	2625.332477	5.66E-37	-1.576337338	-1.160414798
shed9_mers	30	180	-19.9267635	2416.679592	6.35E-82	-4.369221566	-3.58633399
shed9_mers	30	360	-26.6908215	2372.499217	1.81E-137	-8.669872761	-7.483118692
shed9_mers	60	90	-8.10411525	3414.952957	7.33E-16	-1.155422969	-0.7052607913
shed9_mers	60	180	-17.31779957	2645.817458	9.89E-64	-3.94054192	-3.138945259
shed9_mers	60	360	-24.9811945	2471.751814	5.26E-123	-8.238050324	-7.038872753
shed9_mers	90	180	-11.69373787	3517.542247	5.11E-31	-3.046908847	-2.171894572
shed9_mers	90	360	-21.05466074	2876.959433	1.20E-91	-7.332836757	-6.083402559
shed9_mers	180	360	-11.3630179	4039.768937	1.78E-29	-4.805902187	-3.391533711
shed9_sars	5	10	-5.827255498	3656.130339	6.12E-09	-0.338682992	-0.1681546149
shed9_sars	5	30	-15.93551343	2474.271646	1.68E-54	-2.252826441	-1.759139371
shed9_sars	5	60	-24.22986496	2368.615802	4.73E-116	-6.971086089	-5.927204509
shed9_sars	5	90	-29.02840894	2355.912225	1.20E-158	-10.90045147	-9.520916049
shed9_sars	5	180	-37.51261827	2349.174723	9.68E-242	-17.88822631	-16.11091899
shed9_sars	5	360	-44.41122641	2347.597092	0.00E-02	-22.85762255	-20.92442873
shed9_sars	10	30	-13.50287159	2774.263963	2.74E-40	-2.007062693	-1.498065512
shed9_sars	10	60	-23.11520727	2435.017365	4.83E-107	-6.721330254	-5.670122738
shed9_sars	10	90	-28.19426147	2393.845207	2.92E-151	-10.64980848	-9.264721435
shed9_sars	10	180	-36.86377323	2371.998561	1.39E-235	-17.63696377	-15.85534393
shed9_sars	10	360	-43.80706061	2366.882363	1.64E-307	-22.6061865	-20.66902718
shed9_sars	30	60	-15.16883196	3315.627445	2.67E-50	-5.017472824	-3.868851963
shed9_sars	30	90	-22.03199137	2914.401924	1.25E-99	-8.93489356	-7.474508149
shed9_sars	30	180	-31.94341705	2688.597586	4.27E-190	-15.91397128	-14.07320821
shed9_sars	30	360	-39.15460734	2634.866066	1.06E-264	-20.88088669	-18.88919878
shed9_sars	60	90	-8.547204894	4353.439943	1.72E-17	-4.624338675	-2.898738248
shed9_sars	60	180	-20.10736163	3776.451314	1.64E-85	-11.57915939	-9.521695307
shed9_sars	60	360	-27.604769	3591.387458	2.76E-152	-16.53863767	-14.34512301
shed9_sars	90	180	-11.85034185	4405.429079	6.51E-32	-7.91203239	-5.665745388
shed9_sars	90	360	-19.3122369	4228.678752	1.05E-79	-12.86609804	-10.49458572
shed9_sars	180	360	-7.312595344	4645.189812	3.07E-13	-6.202830132	-3.580075851
