# Supplementary material for: Impacts of observation frequency on proximity contact data and modeled transmission dynamics
Source: PLoS Comput Biol. 2023 Feb 27;19(2):e1010917. doi: 10.1371/journal.pcbi.1010917 (PMC9997969; doi:10.1371/journal.pcbi.1010917)
Supplement: S1 Appendix — Auxiliary results for 12 diseases, as well as a diagram illustrating the SEIR-ABM. (PDF) [file pcbi.1010917.s002.pdf]

Illustration of the ABM-SEIR model

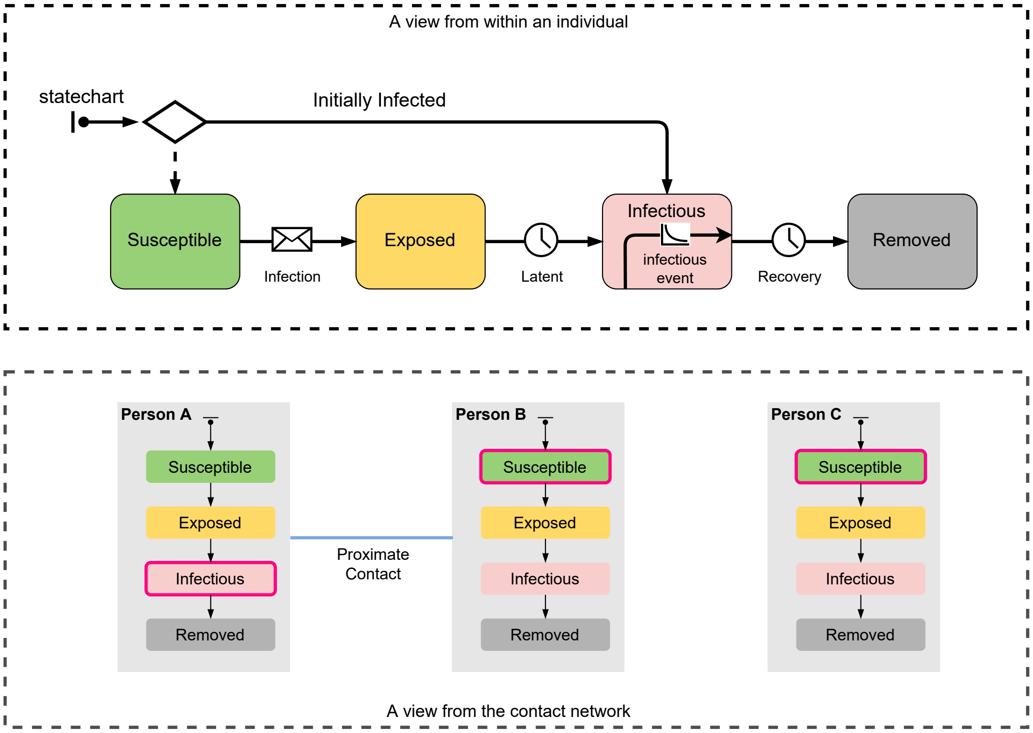

Fig 1: Illustration of the ABM-SEIR model

An agent-based SEIR model extends a System Dynamics SEIR model by shifting the modeling to the state transitions within each individual (agent) and the time-varying connectedness between individuals (as edges in temporal graphs). In the view from within an individual, a statechart use states and transitions between states to reflect the status and relations between status related to disease infection. In the demonstrated statechart, one state will be active at any time, denoting that the individual currently occupies the corresponding status in the natural history of infection. For an infectious individual, their infectious activities — such as sneezing and coughing — are abstracted as infectious events. The transition from the *Exposed* state to the *Infectious* state is parameterized by the latent period realized from a disease's range of latent period. The transition from the *Infectious* state to the *Removed* state is parameterized by the infectious period realized from a disease's range of infectious period. From the perspective of the depicted contact network, the infectious *Person A* may only infect *Person B* but not *Person C* because *Person A* and *Person C* are not in proximate contact at the moment.

Comments on the selected 12 diseases/pathogens

Table 1: Comments on Diseases

Disease/Pathogen Name	Comments
chickenpox	Chickenpox is an airborne infection caused by the varicella-zoster virus (VZV). Chickenpox is highly contagious and spreads mainly through close contact with infectious individuals [1].
COVID-19 Wild Type	COVID-19 is caused by the SARS-CoV-2 virus. Variants of SARS-CoV-2 emerged over time. This pathogen represents the original lineage (non-variant wild type) of the SARS-CoV-2 virus [2].
COVID-19 Alpha Variant	The Alpha variant (B.1.1.7) is a SARS-CoV-2 variant of concern [2]. The Alpha variant is considered more transmissible (+29%, 95% CI: 24–33) than the wild type [3].
COVID-19 Beta Variant	The Beta variant (B.1.351) is a SARS-CoV-2 variant of concern [2]. The Beta variant is considered more transmissible (+25%, 95% CI: 20–30) than the wild type [3].
COVID-19 Delta Variant	The Delta variant (B.1.617.2) is a SARS-CoV-2 variant of concern [2]. The Delta variant is considered far more transmissible (+97%, 95% CI: 76–117) than the wild- type [3].
diphtheria	Diphtheria is caused by strains of bacteria called *Corynebacterium diphtheriae*. Diphtheria bacteria usually spread through respiratory droplets or through touching infectious individuals [4].
fifth	Fifth disease is caused by parvovirus B19. The disease was given the nickname “fifth disease” as one of the six infectious exanthemata of childhood. Parvovirus B19 spreads primarily through respiratory droplets and secondarily household contacts; vertical transmission is also possible [5].
flu	Influenza A is caused by influenza virus type A. Influenza virus type A has many variants. Influenza A viruses are the only influenza viruses known to cause flu pandemics. Infectious individuals can spread flu to others up to about two meters away, primarily through respiratory droplets [6].
measles	Measles is caused by the measles virus. Measles is highly contagious—it can spread to the susceptible through aerosols, respiratory droplets, and contaminated surfaces [7].
MERS	Middle East respiratory syndrome (MERS) is caused by the MERS-CoV virus. MERS spreads through close contact with infectious individuals [8].
pertussis	Pertussis, also known as whooping cough, is caused by a type of bacteria called *Bordetella pertussis*. Pertussis is highly contagious and spreads via respiratory droplets to the susceptible who share breathing space with infectious individuals [9].
SARS	Severe acute respiratory syndrome (SARS) is caused by SARS-CoV infection. SARS is considered to mainly spread by close contact, although spread by respiratory droplets and surfaces is also possible [10].

Descriptive information of SHED data sets

Node degree distriubtion

The degree distribution reflects the fraction of nodes (individuals) in the contact network with node degree x, that is, being in contact with x other individuals. The degree distribution of a contact network is often expressed with the empirical complementary cumulative distribution function (ECCDF) of node degrees [11, 12], or formally $\bar{F}(x) = P(X > x) = 1 - F(X)$, where $F(X) = P(X \leq x)$ is the empirical cumulative distribution function.

Observations of a proximate contact network with sampling interval ξ and sampling method μ are temporal graphs $\left\{G_{t_i;\xi,\mu} = \left(V_{t_i;\xi,\mu}, E_{t_i;\xi,\mu}\right)\right\}$, $t_i \in [i\xi, (i+1)\xi)$, $i = 0, 1, \cdots, n-1$ over effective study period $T = \xi n$. We aggregated these temporal graphs with two distinct methods to describe the structure of an observed contact network parameterized by sampling interval ξ, sampling method μ, and underlying population V.

The graph union method examines the ECCDF of node degrees in the graph union for $\{G_{t_i;\xi,\mu}\}$, defined as $\bar{F}_{\breve{G}_{\xi,\mu}}(x) = P(X_{\breve{G}_{\xi,\mu}} > x)$, where the graph union $\breve{G}_{\xi,\mu} = \bigcup_{t_i} G_{t_i;\xi,\mu} = \bigcup_{t_i} \left(V_{t_i;\xi,\mu}, E_{t_i;\xi,\mu}\right) = \left(\bigcup_{t_i} V_{t_i;\xi,\mu}, \bigcup_{t_i} E_{t_i;\xi,\mu}\right)$. In other words, the graph union method aggregates temporal graphs into a aggregated graph with distinct individuals who have ever been observed in any of those temporal graphs. In such aggregated graph, a pair of individuals are connected as long as they have been in contact with each other in any of those temporal graphs. The graph-union aggregated degree distribution relates to the fraction of individuals for whom the upper bound of the number of secondary cases that this individual can cause throughout a realization of the simulation is x. The upper bound of the number of secondary cases one case would produce corresponds to the hypothetical scenario when an infectious infected every contact they have ever had in an otherwise susceptible population. Research has shown that the graph union method of temporal aggregation, if used for resampling and generating new networks, can result in overestimation of the cumulative incidence of disease induced by those networks [13].

The degree union method examines the ECCDF of degrees that a node has within a temporal graph, that is, the ECCDF of the number of distinct contacts an individual has within a sampling interval. Formally, the degree-union aggregated degree distribution is the ECCDF of node degrees, defined as $\bar{F}_{\bar{G}_{\xi,\mu}}(x) = P(X_{\bar{G}_{\xi,\mu}} > x) = \frac{1}{\sum_i \|V_{t_i}^+\|} \sum_{i, v \in V_{t_i;\xi,\mu}^+} \mathbb{I}_{\deg(v) > x}$, $i = 0, 1, \cdots, n-1$, where $V_{t_i;\xi,\mu}^+ = \left\{v \in V_{t_i;\xi,\mu} \,\middle|\, \deg(v) > 0\right\}$, $\deg(v) = \left\|V_{t_i;\xi,\mu}^{(v)}\right\|$, and $V_{t_i;\xi,\mu}^{(v)} = \{w \mid e_{vw} \in E_{t_i;\xi,\mu},\ t_i \in [\xi i, \xi i + \xi)\}$. In other words, the degree union method calculates node degrees in each temporal graph, and then pools the node degrees from different temporal graphs and examines the ECCDF of the pooled node degrees. The degree-union aggregated degree distribution relates to the faction of individuals that have been in contact with x distinct individuals during a sampling interval ξ. The infectious period of an infectious usually spans over multiple consecutive sampling intervals.

For graph-union aggregated degree distributions, the `Snapshot` method underestimate node degrees as the sampling interval ξ increases (Fig 2), while the `Upperbound` method is free from this underestimation (Fig 3). In contrast, for degree-union aggregated degree distributions, the `Upperbound` method overestimates node degrees as the sampling interval ξ increases (Fig 4), while the `Snapshot` method is free from this overestimation (Fig 5).

The differences between a "diffuse" underlying population (such as SHED9) versus a "closer" underlying population (such as SHED1) is reflected by the number of distinct contacts that an individual could have within a sample interval ξ (or a short period of time). For example, in both Figs 4 and 5, SHED1 has a larger fraction of individuals than SHED9 that have multiple distinct contacts (with node degree larger than one) within a single sampling interval. For example, in above 50% of the cases, an individual from SHED1 has at least one contact per six hours (360 minutes)—this chance is reduced to roughly 12.5% for an individual from SHED9 (Fig 5).

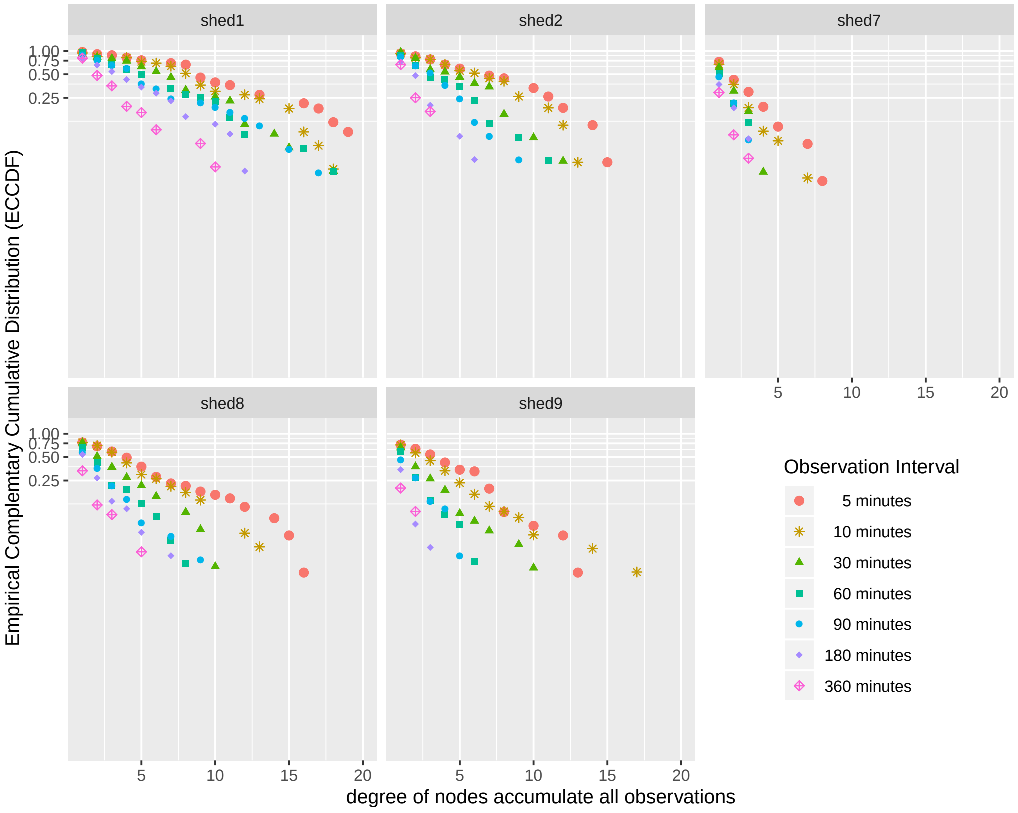

Fig 2: The graph union method aggregated degree distribution for the `Snapshot` method

Comparison of distortions on the graph-union-aggregated degree distribution when with successive levels of downsampling. Degree distributions of networks are shown in the form of empirical complementary cumulative distribution functions (ECCDFs) without smoothing and interpolation, where the x-axis is node-degree x and y-axis is $P(X > x)$. Each leftmost point has its x-value denoting a degree of 1, and its y-value $P(X > 1)$ denoting the proportion of nodes having node degree $x \geq 2$. Nodes with degree of 0—that is, lacking connection to other nodes—are filtered out before plotting the ECCDFs, thus $1 - P(X > 1) = P(X \leq 1) = P(X = 1)$.

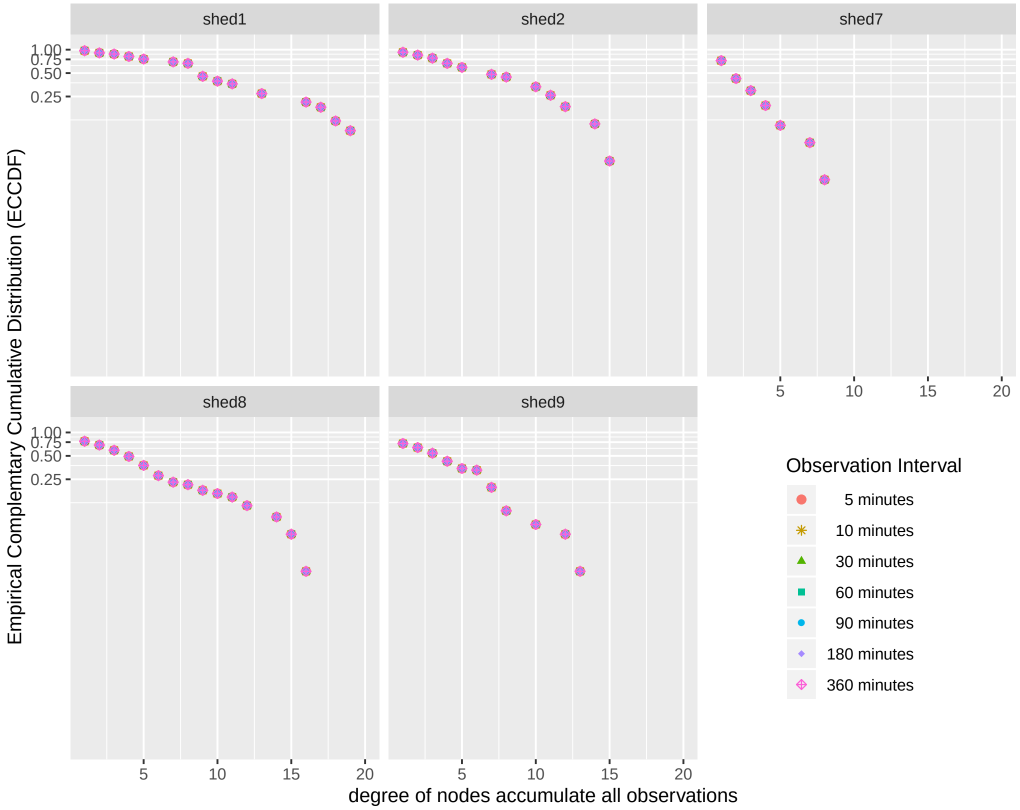

Fig 3: The graph union method aggregated degree distribution for the `Upperbound` method

See the caption of Fig 2.

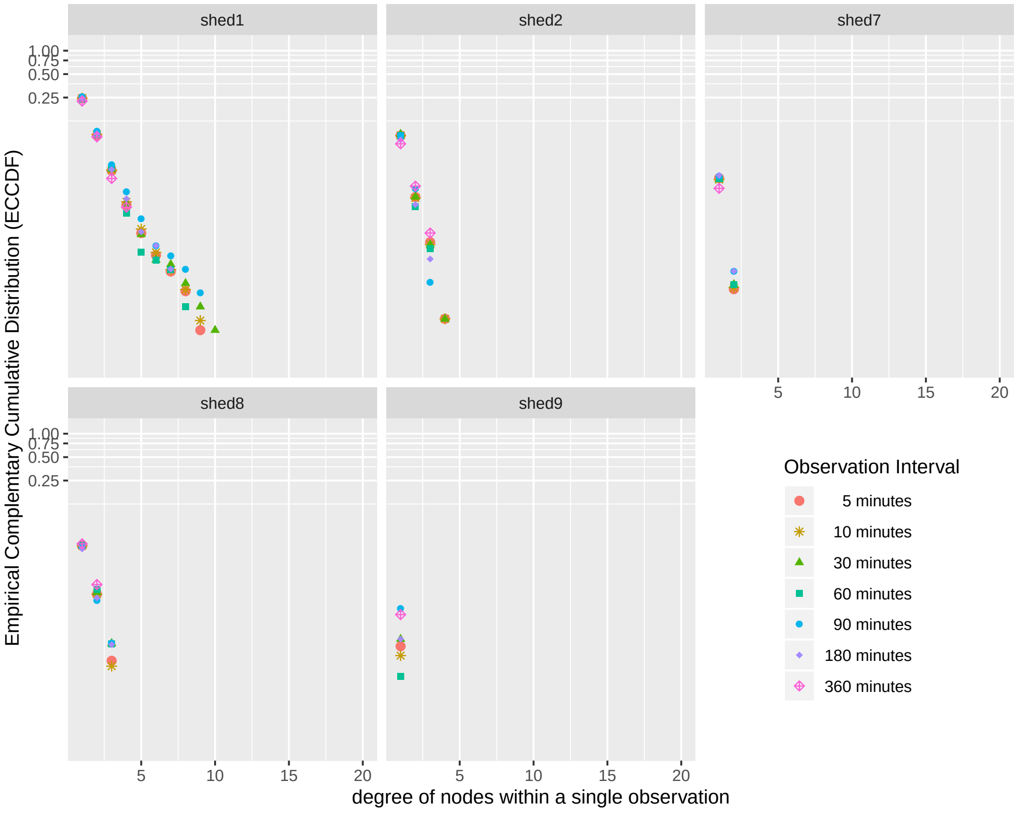

Fig 4: The degree union method aggregated degree distribution for the `Snapshot` method
Comparison of distortions on the degree-union-aggregated degree distribution when with successive levels of downsampling. Degree distributions of networks are shown in the form of empirical complementary cumulative distribution functions (ECCDFs) without smoothing and interpolation, where the x-axis is node-degree x and y-axis is $P(X > x)$. Each leftmost point has its x-value denoting a degree of 1, and its y-value $P(X > 1)$ denoting the proportion of nodes having node degree $x \geq 2$. Nodes with degree of 0—that is, lacking connection to other nodes—are filtered out before plotting the ECCDFs, thus $1 - P(X > 1) = P(X \leq 1) = P(X = 1)$.

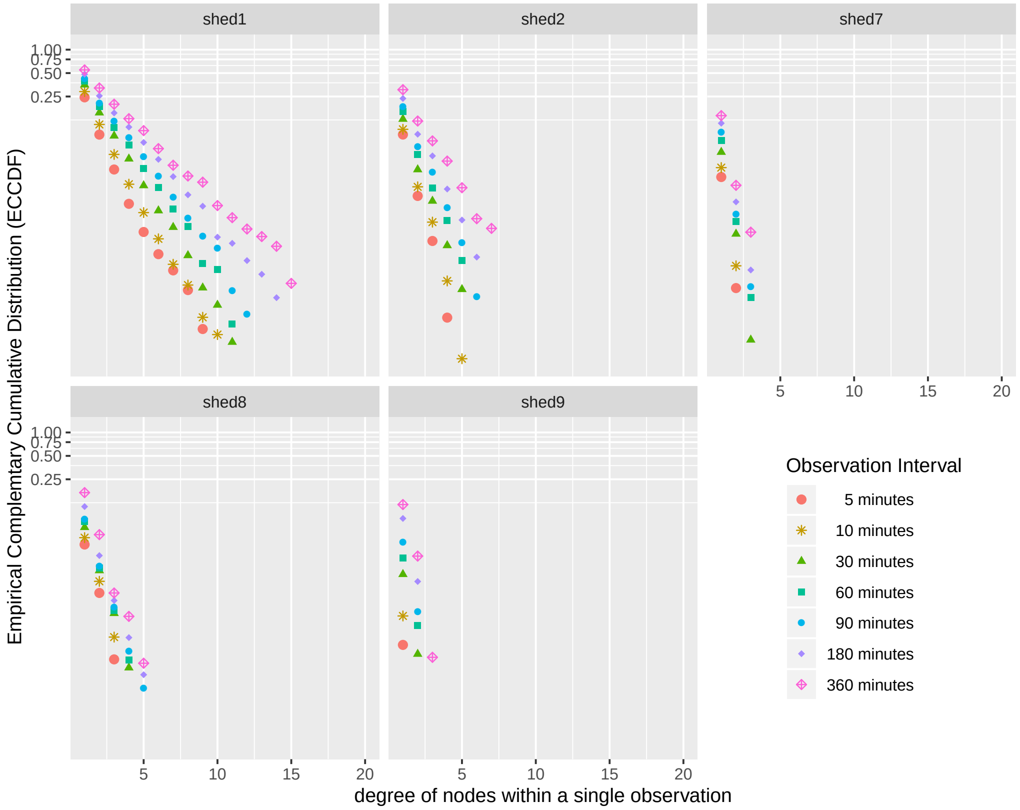

Fig 5: The degree union method aggregated degree distribution for the `Upperbound` method
See the caption of Fig 4.

Results based on the selected 12 diseases/pathogens

Impacts on population-level simulation results

The impacts of the observation frequency on simulation results from the `ABM-SEIR` model can be considered at the population and/or individual level. Cumulative cases and attack rates were used to measure the impacts of observation frequency on simulation results at the population level—population-level results of a transmission model are often used to evaluate the size of the outbreak or the overall severeness of an upcoming wave. We performed Welch's t-test on cumulative cases with different ξ to draw quantitative conclusions as to the impact of observation frequency on the mean of cumulative cases. We used the Prentice modified Friedman tests on cumulative cases with different ξ to test the impact of observation frequency on the distribution of cumulative cases.

Cumulative cases

Figs 6 and 7 show grids of violin plots visualizing the empirical distributions of the cumulative cases in realizations of the agent-based SEIR model taking downsampled contact data at different duty cycle intervals, with one grid for each of the `Snapshot` and the `Upperbound` downsampling methods. Each grid of the violin plots characterizes how cumulative cases varies by diseases (row) and underlying populations (column). Each cell of a grid is a violin plot consists of violins arranged by increasing duty cycle interval, with 5 minutes being the left-most and 360 minutes being the right-most. Each violin in a violin plot illustrates the distribution of cumulative cases for realizations given the duty cycle interval (x-axis value), the disease/pathogen (row-label), and the underlying population (column-label)—aggregated over random seeds and index infectives.

Violin plots of cumulative cases illustrate the risk of outbreak occurrence. In general cases, the `Snapshot` method preserves the distribution of cumulative regardless of increasing duty cycle interval. Meanwhile, the `Upperbound` method suffers from systematically overestimating the plausibility of having an outbreak, except for diseases with low R_0 (MERS). For diseases having relatively high R_0 (e.g., chickenpox, measles, pertussis) and close population (`SHED1-2`), the `Snapshot` method risks underestimating plausible outbreaks with sparse observations—those sampled an hour or more apart—while the `Upperbound` method retains the risk of corresponding outbreak occurrence at the cost of results varying between either universal infection or no further infection after the initial infection.

Welch's *t*-test We validated our interpretation of Figs 6 and 7 with the Bonferroni-corrected Welch's t-test [14, 15], provided by R package *stats*, version 4.0.2. For each sampling method of the `Snapshot` and `Upperbound`, we tested cumulative cases with different duty cycle intervals blocked by diseases and underlying populations. Resulting 60 blocks, with each group (observation frequencies) having at least 960 samples (cumulative cases of realizations), sufficiently large to consider the robustness of t-test given the distribution of cumulative cases' departure from normality [16, 17], as shown in Figs 6 and 7. Setting the alpha-value as 5%, our null hypothesis is that given a disease/pathogen other than high R_0 diseases/pathogens (chickenpox, measles, pertussis) and a underlying population, the mean of cumulative cases resulted by proximate contacts collected with different observation frequencies are equal. For each block, pairwise by duty cycle intervals resulting 21 comparisons per block and $\alpha_{\text{altered}} = 0.05/21 = 0.00238$. It turned out for the `Snapshot` method null hypotheses were not rejected for pairs of observation intervals less than or equal to 30 minutes, except for SHED8-diphtheria between pairs of duty cycle intervals 10–30 $\big(t(4368.3) = -3.10115,\, p = 0.00194\big)$, SHED8-SARS 5–30 $\big(t(4294.5) = -3.23677,\, p = 0.00122)\big)$, and SHED9-diphtheria 5–30 $\big(t(4399.6) = -3.61278,\, p = 0.00031\big)$. For the `Upperbound` method hypotheses are rejected, except for SHED2-fifth 5–10 $\big(t(1910.7) = -1.8350,\, p = 0.06666\big)$, SHED2-MERS 5–10 $\big(t(1902.2) = -1.037,\, p = 0.29989\big)$; SHED9-COVID19Delta 90–180 $\big(t(4667.3) = -1.2404,\, p = 0.21490\big)$, 180–360 $\big(t(4675.8) = -1.9465,\, p = 0.05165\big)$. Full results of Welch's t-tests can be found in S1 Table.

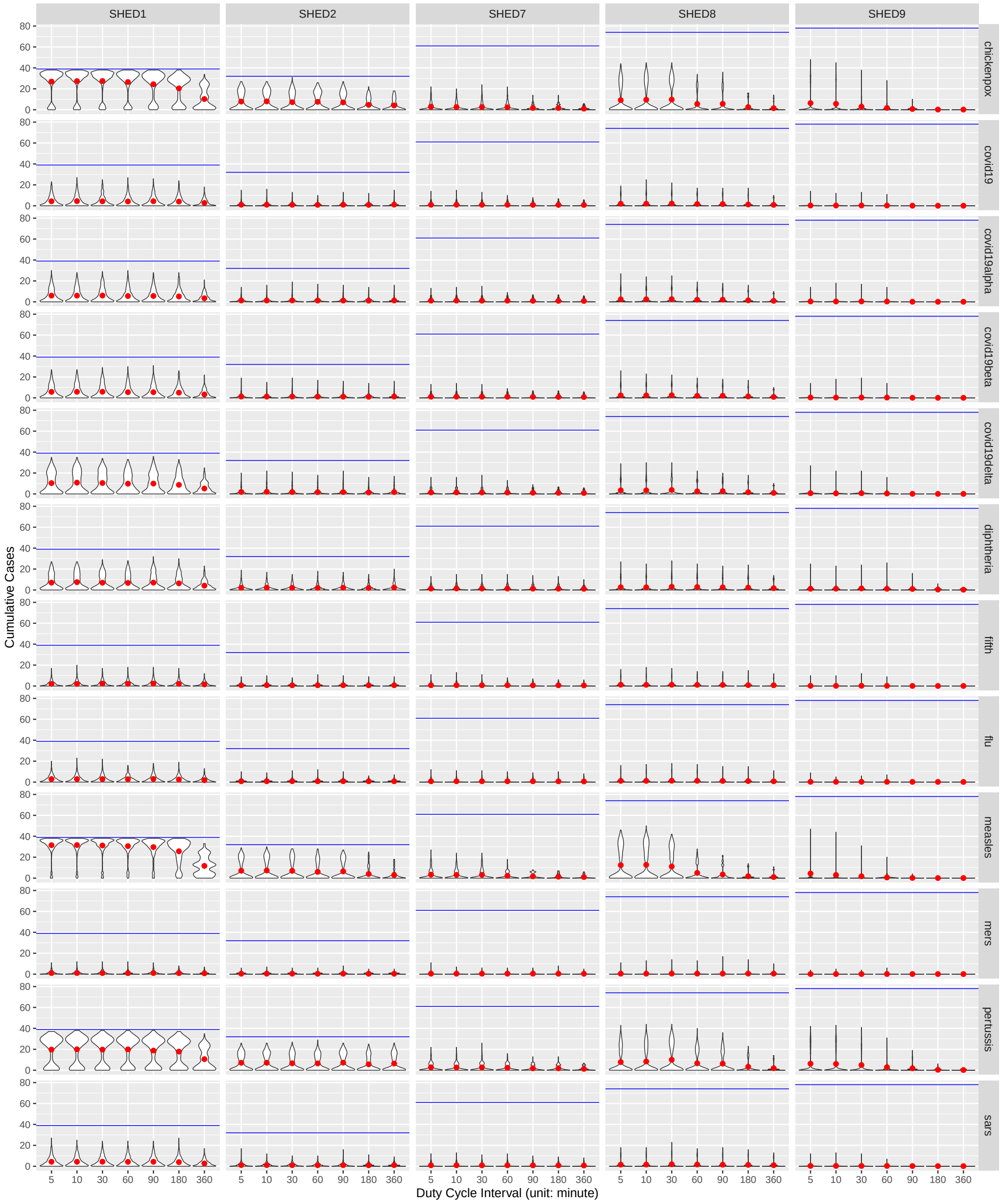

Fig 6: Grids of violin plots of cumulative cases for the `Snapshot` method
Violin plots visualize the empirical distributions of the cumulative cases varying by informing the agent-based SEIR model with proximity contact data downsampled at different inter-observation intervals, given different diseases, within different underlying populations. The size of underlying population is denoted with blue lines, and the red dots indicates the mean values of the corresponding violin plots.

Prentice-modified Friedman tests We further validated our interpretation of Figs 6 and 7 with the Prentice modified Friedman test [18–20], provided by R package *muStat*, version 1.7.0. We tested cumulative cases grouped by sampling interval and blocked by data collection, sampling method, population (dataset), disease, and initial infection node. Resulting $\chi^2 = 222081$, with 6 degrees of freedom (reflecting the fact that the sampling interval $\xi \in \{5, 10, 30, 60, 90, 180, 360\}$ has 7 choices in total), and $p < 2.2\text{e}{-16}$, with the null hypothesis being that the sampling interval does not differentiate the distribution of cumulative cases, for the same data collection, sampling method,

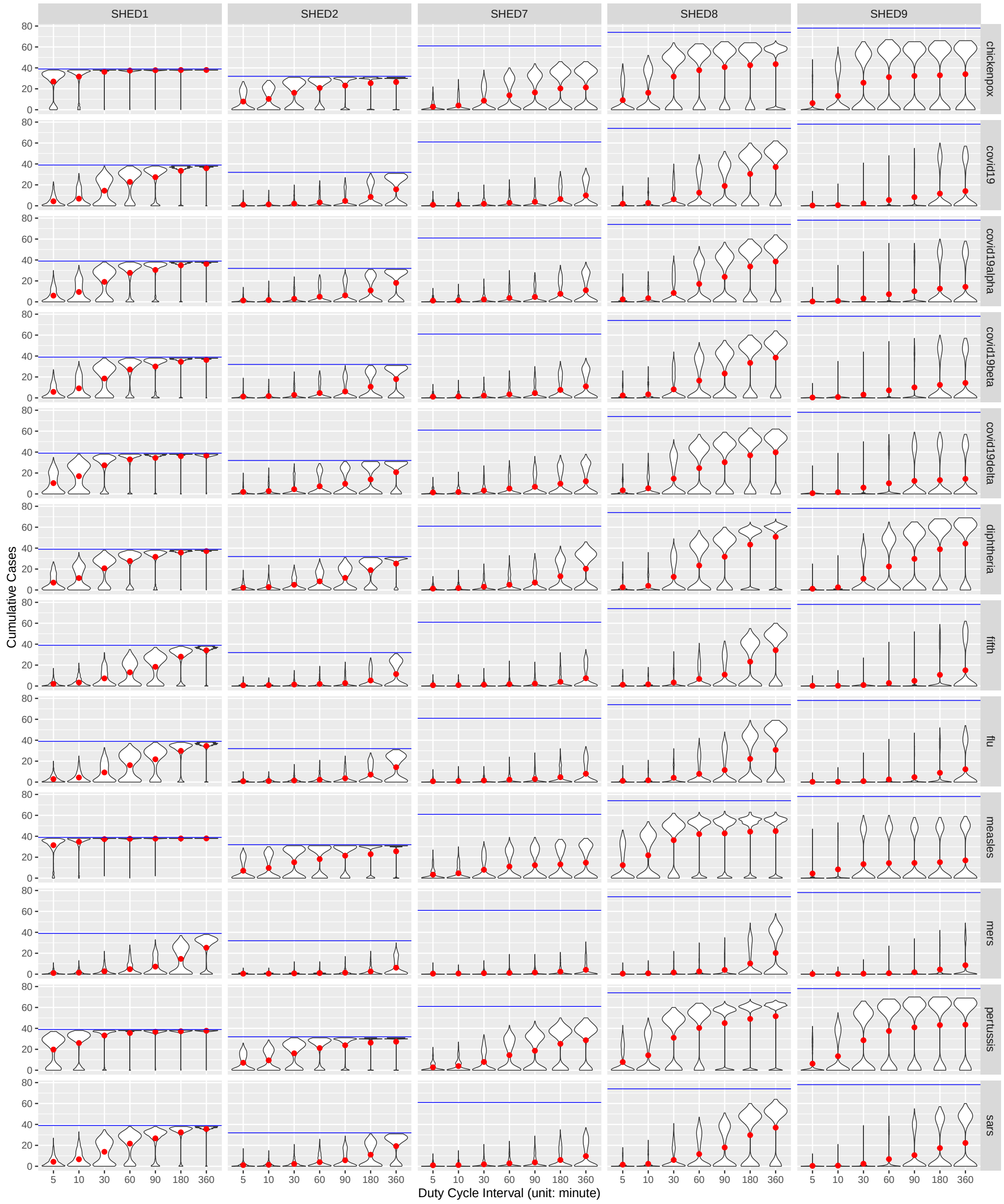

Fig 7: Grids of violin plots of cumulative cases for the `Upperbound` method
See the caption of Fig 6.

dataset, disease, and initial infection node.

Attack rate

The accuracy-precision view measures the deviation with respect to the attack rate of simulations parameterized by the downsampled contact observations $\mathcal{D}_{\xi'}$, $\xi' \in \{10, 30, 60, 90, 180, 360\}$ from the baseline $\mathcal{D}_{\xi_0}$, $\xi_0 = 5$. Subplots are arranged as grids according to the combinations of underlying population V and disease $\mathcal{M}$. Within each subplot specific for a given combination of $\{\mathcal{D}, \mathcal{V}, \mathcal{M}\}$, deviation of the median attack rate is shown on the horizontal axis (reflecting accuracy), and deviation of the inter-quartile range (IQR) for attack rate is depicted on the vertical axis (negatively

correlated with precision). Each datapoint within such a subplot is associated with a specific sample interval ξ of $\mathcal{D}$, whose value is denoted by both color and shape for visual clarity. In both Figs 8 and 9, points with the same color and shape tend to cluster instead of mixing with other colors, indicating that sample interval impacts govern both the accuracy and precision of the attack rate more than the initial infection node. In Fig 8, when downsampling with the `Snapshot` method, points are closer to the origin for communicable diseases/pathogens with low R_0 and for "diffuse" population such as $\{\text{SHED7}, \text{SHED8}, \text{SHED9}\}$, indicating the advantage of `Snapshot` at maintaining an estimate of attack rate as downsampling interval increases. For diseases with high R_0 (chickenpox, measles, pertussis) and "closer" communities $\{\text{SHED1}, \text{SHED2}\}$, `Snapshot` underestimates the attack rate as ξ increases, whereas `Upperbound` slightly overestimates. `Upperbound` reduces IQR deviation of estimated attack rate while the `Snapshot` increases interquartile range (IQR) deviation.

In Figs 8 and 9, we summarized two statistics: median and IQR, across the values of the attack rate drawn from an ensemble of 30 realizations for each scenario defined by observations of contact network $\mathcal{D}$, initial infectious individual $\mathcal{V}$, and a type of communicable disease $\mathcal{M}$. The accuracy-precision deviation of simulation results in terms of attack rate depends on the underlying population structure ("closer" or "diffuse"), the type of communicable disease, the sampling method (`Snapshot` or `Upperbound`), and the sample interval. The sampling interval is denoted with color. Fig 8 depicts median and IQR specifically for the `Snapshot` sampling method, while Fig 9 depicts for the `Upperbound` sampling method. Casual inspection of the skewed nature of the distributions towards higher values of the horizontal axis within each subplot (indicating increasing median deviation in incidence) confirms that increasing the sample interval results in over-estimation of the attack rate, as is suggested in [13]. By contrast, the clustering of the points by color in each subplot suggests that the initial infection node exerts a smaller impact on the two statistics we have chosen to reflect the accuracy-precision tradeoffs.

Comparing within subplots column-wise, the `Snapshot` method performs well with "diffuse" communities, resulting in both low deviation of median and low deviation of IQR. When used with close networks, `Snapshot` tends to overestimate the attack rate but underestimate the IQR. `Upperbound` exhibits greater deviation than `Snapshot`, and is more consistent as sampling interval increases given other factors—from left to right. When sampling interval is brief and sampling rate high, attack rate exhibits low median and IQR deviation from the ground truth, because the reconstructed contact network is less distorted. As the sampling interval increases and sampling rate decreases, `Upperbound` tends to become both less accurate and less precise. As the sample interval increases further, the overestimation of the attack rate reaches a limit as people directly or indirectly connected to the initially infectious person are reliably infected for high R_0 pathogens, or people remain uninfected for pathogens with low R_0.

Comparing within subplots row-wise, disease-specific patterns are also visible: estimates for the attack rate of diseases with low R_0, such as MERS, seem relatively insensitive to the sampling interval. Pathogens/communicable diseases with sufficiently high R_0 tend to behave similarly as sampling interval increases, regardless of their differences in R_0 value.

Outbreaks and outbreak timing

Outbreak timing and behavior are commonly studied characteristics of communicable diseases, yet the quantifiable definition of an outbreak varies due to challenges regarding data collection and characterization of the appropriate cohort to be counted. Instead of imposing a quantitative definition, this work employs cumulative cases over time as a measure to reflect outbreak dynamics of disease in simulations for a given underlying population V and observed contact data $\mathcal{D}$.

Each realization of the ABM-SEIR simulates an observation of cumulative cases over time as a continuous time series $\zeta^{(i)}(t; \mathcal{V}, \mathcal{D}_{\xi,\eta}, \mathcal{M})$, where $i = 1, 2, \cdots, N_{\text{MC}}$ is the index of Monte Carlo replication and t is the time in simulation, ranging from zero up to the time of termination of that realization. Recall that a realization terminates once its last infectious individual finished their infectious period and became *Removed.* The continuous time series of cumulative cases vary under the circumstances specified by the initial infectious individual $\mathcal{V}$ (implies the underlying population

V), observed contact data $\mathcal{D}_{\xi,\eta}$ (implies the sampling method η and sampling interval ξ), and disease $\mathcal{M}$ (parameterizes latent period and infectious period). The average of $\zeta^{(i)}$ over different initial infectious individual $\mathcal{V}$ and the Monte Carlo replication index i, denoted by $\hat{\mu}_\zeta(t; \mathcal{D}_{\xi,\eta}, \mathcal{M}) = \frac{1}{N_{\text{MC}}\|V\|} \sum_{\mathcal{V}\in V,\, i=1,2,\cdots,N_{\text{MC}}} \zeta^{(i)}(t; \mathcal{V}, \mathcal{D}_{\xi,\eta}, \mathcal{M})$, is the sample mean of cumulative cases given the underlying population V and observed contact data $\mathcal{D}_{\xi,\eta}$ for the disease $\mathcal{M}$. Assuming a homogeneous chance for each individual of the underlying population V to become the initial infectious individual, we can use $\hat{\mu}_\zeta(t; \mathcal{D}_{\xi,\eta}, \mathcal{M}) \in [0, \|V\|]$ to estimate the expected cumulative cases.

To compare across underlying populations of different population size $\|V\|$, we defined the normalized expected cumulative cases (NECC) as $\hat{\varrho}(t; \mathcal{D}_{\xi,\eta}, \mathcal{M}) = \frac{1}{\|V\|} \hat{\mu}_\zeta(t; \mathcal{D}_{\xi,\eta}, \mathcal{M})$, where $\hat{\varrho}(t; \mathcal{D}_{\xi,\eta}, \mathcal{M}) \in [0, 1]$, with 0 indicating no infection and 1 indicating having entire population infected by time t. For a disease $\mathcal{M}$, given the underlying population V and observed contact data $\mathcal{D}_{\xi,\eta}$, the NECC reflects the estimated expected fraction of maximum potential cumulative cases at time t.

We selected four representatives as shown in Fig 10. Similar diseases appear to have similar dynamics, as shown, for example, in Fig 10A and Fig 10B; diseases with extremely low and high R_0 tend to behave quite differently regardless of sampling method, interval, and dataset, as can be seen by contrasting Fig 10C and Fig 10D. In general, the NECC curves of the `Snapshot` method exhibit modest estimations on cumulative cases (on the vertical axis) over time. As the downsampling interval increases, the NECC curves of the `Snapshot` method also tend to end earlier on the horizontal axis. This early termination is because disconnections halt the pathogen spread among infectious and susceptible due to missed contacts. For a given underlying population, the early termination of disease spread seems consistent with modest estimations on cumulative cases, except for the scenarios when NECC curves terminated early due to the entire population having been infected. This analysis demonstrates that:

- As would be expected, given an initially susceptible population, a pathogen with a tendency to catalyze an outbreak will often exhibit an apparent, sharp increases in infections during the outbreak period. Weakly spreading pathogens have an initial ascent followed by a long tail. More notable is that this tendency holds largely invariant of sampling method, population, and sampling interval.

- In a pattern that is maintained—*mutatis mutandis*—across populations, sampling method, and interval, similar diseases exhibit clinically similar curves: SARS (Fig 10B) is known to have similar characteristics to flu (Fig 10A), and they exhibit similar patterns for our measure. The discrepancy is small for "closer" populations.

- In a pattern that again holds independent of sampling method and interval as well as population (dataset), diseases with different R_0 behave differently. Pertussis (Fig 10C) has the highest R_0 amongst the diseases we simulated, while MERS (Fig 10D) has the lowest. Their pattern is distinct—pertussis tends to have a clearer outbreak. By contrast, SARS exhibits a steep curve in the beginning and a long tail, indicating limited disease spread.

- Discrepancies between `Snapshot` and `Upperbound` from the baseline increased with the sampling interval ξ. Discrepancies induced by the sampling interval exert less impact than the characteristics of the study population, with "diffuse" communities (like SHED9) exhibiting substantial discrepancies for both `Snapshot` and `Upperbound`.

- When the sampling interval is large, the `Snapshot` method outperforms `Upperbound` in terms of having NECC closer to the baseline, at the cost of shortening the disease spreading period.

Impacts on individual-level simulation results

We measure the impacts of observation frequencies on the simulation results at the individual level with transmission pathways and individual infection risks. Individual risk of infection can suggest vulnerable group to prioritize resource allocation and ensure health equity [21]. Individual risk

of infection is asymptotically approached by the fraction of realizations in which an individual is infected. The difference of individual risk of infection can be compared pairwise in terms of weighted-Minkowski distance among scenarios with different datasets $\mathcal{D}$ for the same underlying population V and disease $\mathcal{M}$. We calculated the Kullback-Leibler divergence on individual infection probabilities with different ξ to draw quantitative conclusions on the impact of downsampling frequency on simulation results at the population level. Higher KL-divergence values from the `Snapshot` method for `SHED9` were observed for chickenpox, COVID-19, diphtheria, measles, and pertussis, and are indicated by reddish colors of the corresponding column on Fig 11. Lower KL-divergence values associated with MERS, regardless of dataset and downsampling frequency, induces its greenish color in the corresponding column in that figure. We find that the KL-divergence can effectively summarize the information shown on Fig 11 and therefore can serve as an efficient metric to measure differences in individual risk.

Distances matrices of infection pairs

Fig 11 shows matrices of pairwise weighted-Minkowski distances of frequencies of infections pairs given downsampling methods, disease, and sampling frequencies for underlying populations, with the color shifting from greenish to reddish with the increasing degree of dissimilarity. For each matrix, starting from its top left corner, inter-observation intervals are arranged in ascending order—$\xi = 5, 10, 30, 60, 90, 180, 360$—horizontally from left to right and vertically from top to bottom. We found `Snapshot` is better at preserving consistent frequencies of infection pairs, particularly with an observation frequency higher than once per half-hour, except for higher R_0 diseases in "diffuse" communities, such as chickenpox and measles in SHED9. For lower R_0 diseases in general, particularly in "closer" communities like SHED1, the `Snapshot` method have weighted-Minkowski distance less than 1 even between the observation frequencies of 5-minute and 360-minute.

We found `Upperbound` is better at preserving likely paths than `Snapshot`, and the limits of the sampling interval needed to preserve likely paths of disease spreading lies amongst $\xi \in \{10, 30, 60\}$. Under `Upperbound`, diseases with similar R_0 resemble each other, and MERS with a low $R_0 = 0.69$, has its likely paths varying notably over sampling intervals for a less "diffuse" population, while other diseases—despite exhibiting a wide range of $R_0 \in [0.69, 15]$—maintain a similar pattern of those likely paths with rising sampling interval, for a given population.

Kullback-Leibler divergence on individual infection risk

For each individual given each combination of disease and datasets, we calculated the Laplacian-smoothed individual infection probability based on infection counts from simulations fed with ξ-sampled contact data using downsampling method η, where $\eta \in \{\texttt{Upperbound}, \texttt{Snapshot}\}$; then we assembled the individual infection probability into a vector of the individual infection risk, denoted $\boldsymbol{\rho}_{(\mathcal{M},\mathcal{D}_{\xi,\eta})}$. Laplacian-smoothing was employed to ensure that those who were not infected in simulation outcomes are still assigned a small probability of being infected.

To characterize $\boldsymbol{\rho}_{(\mathcal{M},\mathcal{D}_{\xi,\eta})}$ as shown in Figs 12 and 13, we arranged the presentation top-down, characterizing the distinct downsampling methods (η) using two sub-figures, with disease ($\mathcal{M}$) within each sub-figure as wrapped facets. Finally, within each facet, we plotted a line for each underlying population, with the x-value as the duty cycle interval ξ, and the y-value as $\delta_{\xi_+} = D_{\mathrm{KL}}(\boldsymbol{\rho}_{(\mathcal{M},\mathcal{D}_{\xi_0,\eta})} \parallel \boldsymbol{\rho}_{(\mathcal{M},\mathcal{D}_{\xi_+,\eta})})$, where $\xi_0 = 5$, and $\xi_+ \in \{10, 30, 60, 90, 180, 360\}$.

As shown in Fig 12, the `Snapshot` method in general will exhibit higher divergence than the `Upperbound` method, except for diseases with low R_0, such as influenza type A (1.31) and MERS (0.69). In general, the higher the δ_{ξ_+}, the higher the divergence of individual infection risk from estimations with ξ_+-downsampled contact data when compared to ξ_0-sampled contact data.

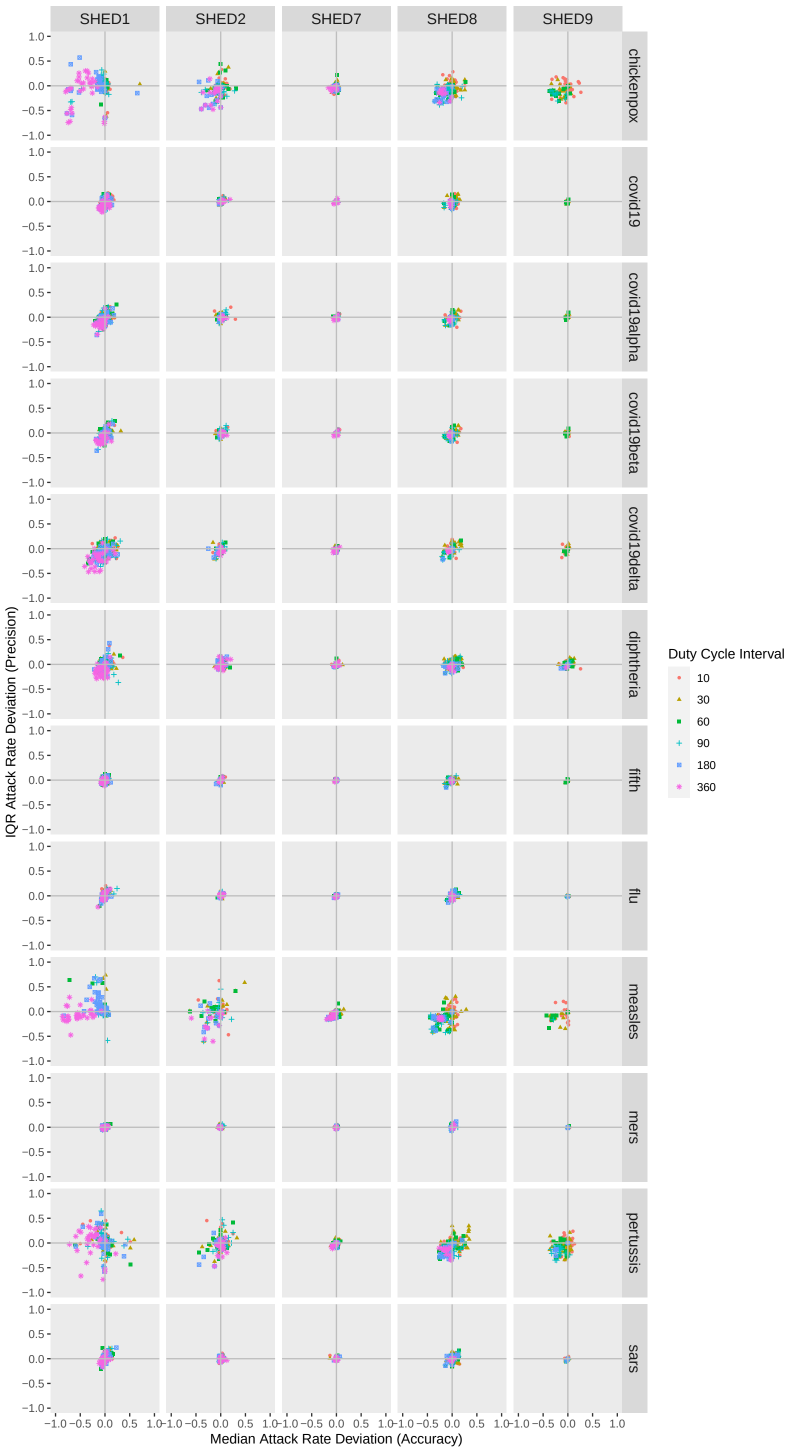

Fig 8: Attack rate given initial infection node for the `Snapshot` method

The accuracy-precision view measures the deviation with respect to the attack ratio of simulations parameterized by the downsampled contact observations $\mathcal{D}_{\xi_+},\ \xi_+ \in \{10, 30, 60, 90, 180, 360\}$ from the baseline $\mathcal{D}_{\xi_0},\ \xi_0 = 5$. Subplots are arranged as grids according to the combinations of underlying population V and disease $\mathcal{M}$. Within each subplot specific for a given combination of $\{\mathcal{D}_\xi, \mathcal{V}, \mathcal{M}\}$, deviation of the median attack rate is shown on the horizontal axis (reflecting accuracy) and deviation of the inter-quartile range (IQR) for attack rate is depicted on the vertical axis (negatively correlated with precision). Each datapoint within such a subplot is associated with a specific sample interval ξ of $\mathcal{D}_\xi$, whose value is denoted by both color and shape for visual clarity. In both Fig 8 and Fig 9, points with same color and shape tend to cluster instead of mixing with other colors, indicating that sample interval impacts govern both the accuracy and precision of the attack ratio more than the initial infection node $\mathcal{V}$.

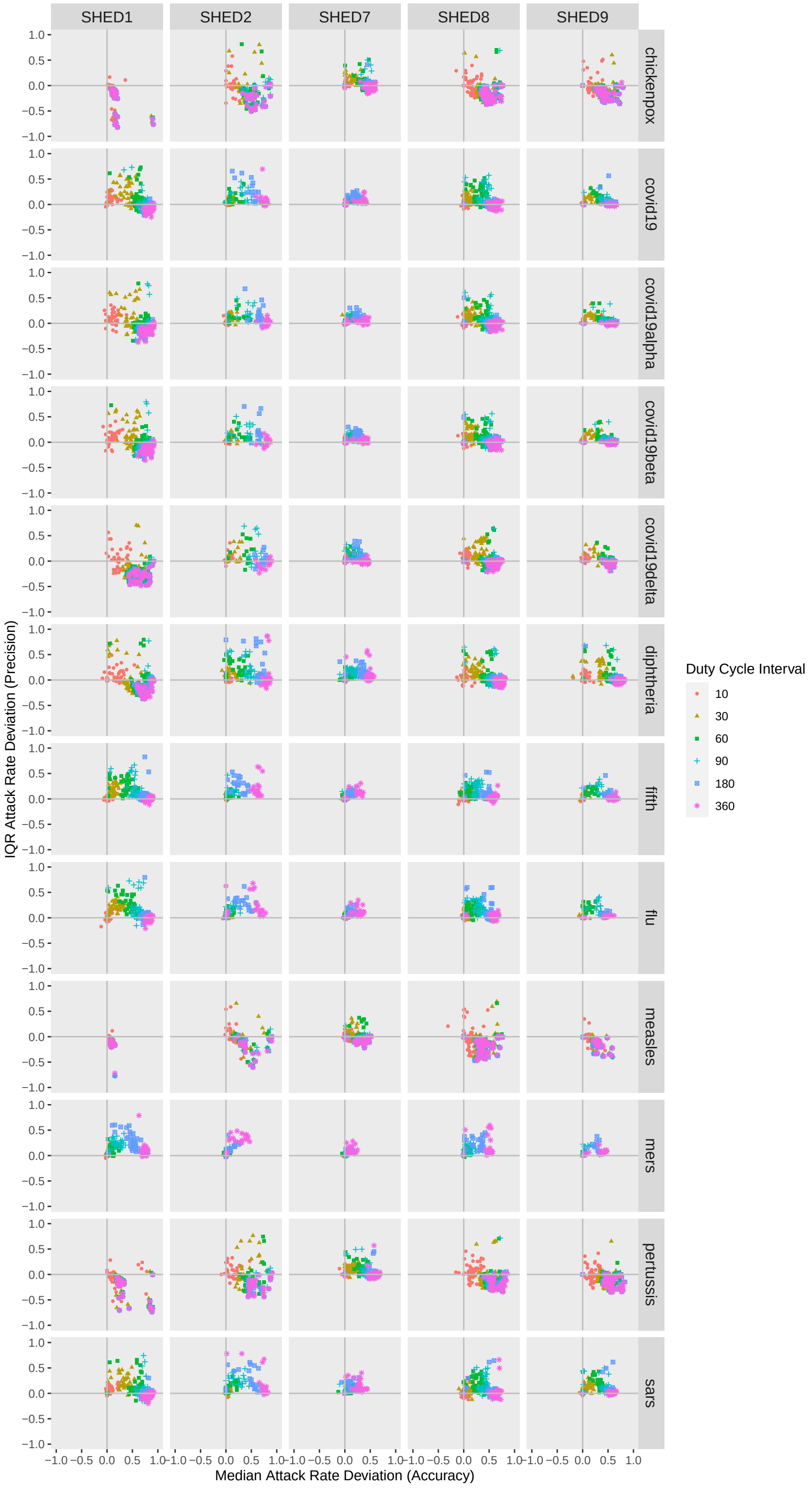

Fig 9: Attack rate given initial infection node for the `Upperbound` method

See the caption of Fig 8.

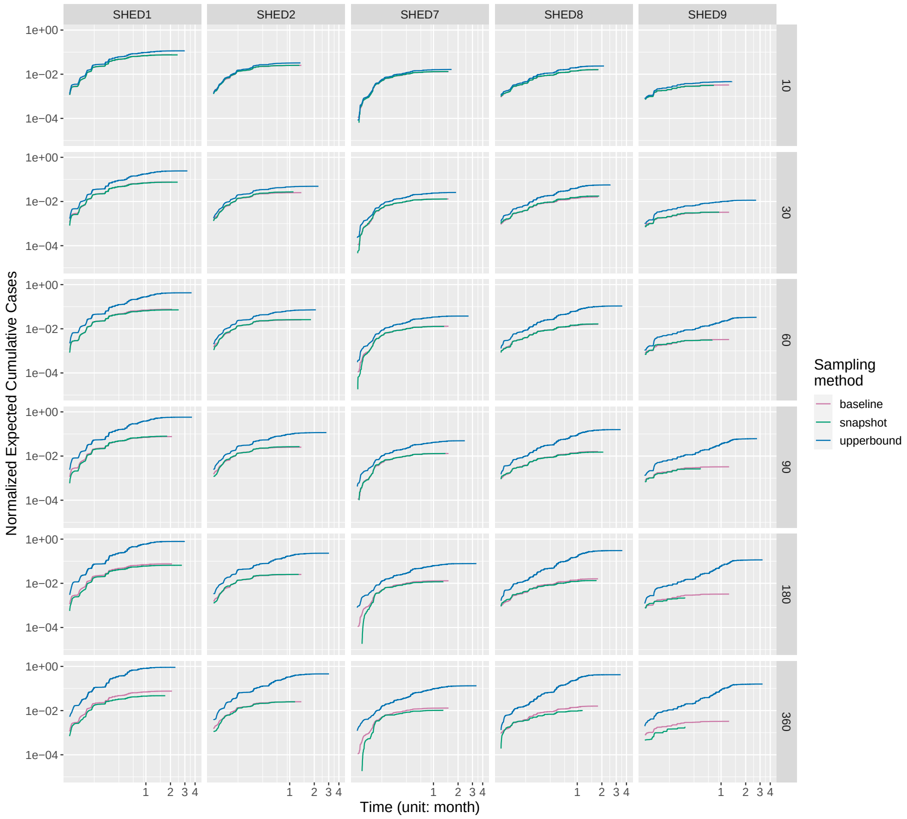
SHED1
SHED2
SHED7
SHED8
SHED9
1e+00
1e−02
1e−04
10
30
60
90
180
360
Normalized Expected Cumulative Cases
Time (unit: month)
1
2
3
4
Sampling method
baseline
snapshot
upperbound
(A) flu

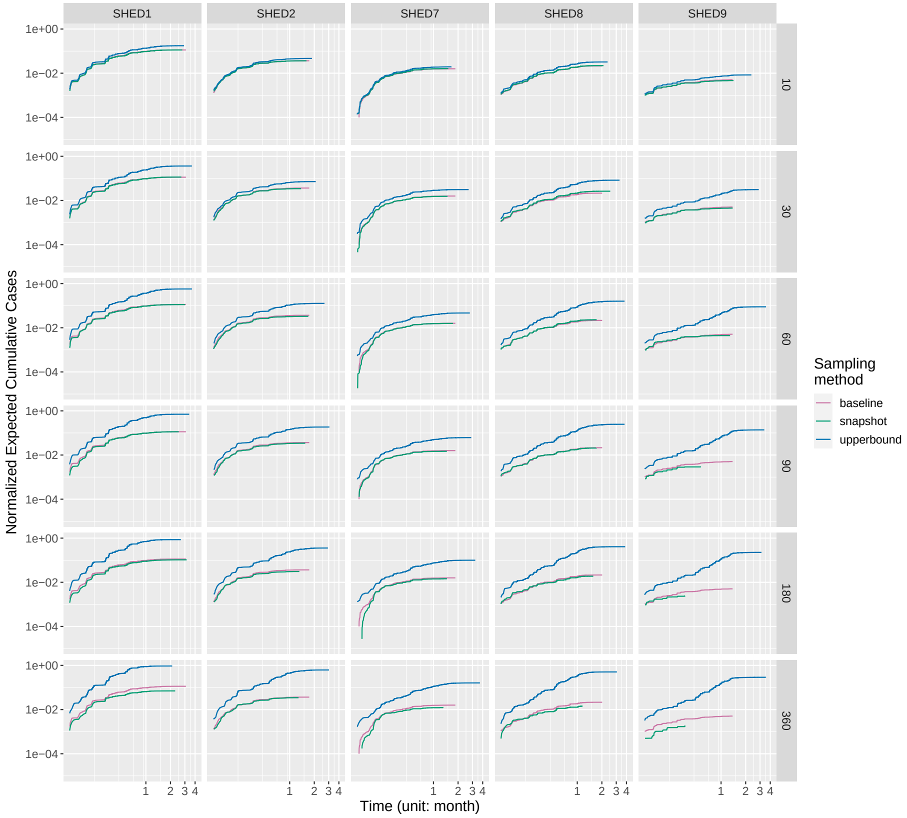
SHED1
SHED2
SHED7
SHED8
SHED9
1e+00
1e−02
1e−04
10
30
60
90
180
360
Normalized Expected Cumulative Cases
Time (unit: month)
1
2
3
4
Sampling method
baseline
snapshot
upperbound
(B) SARS

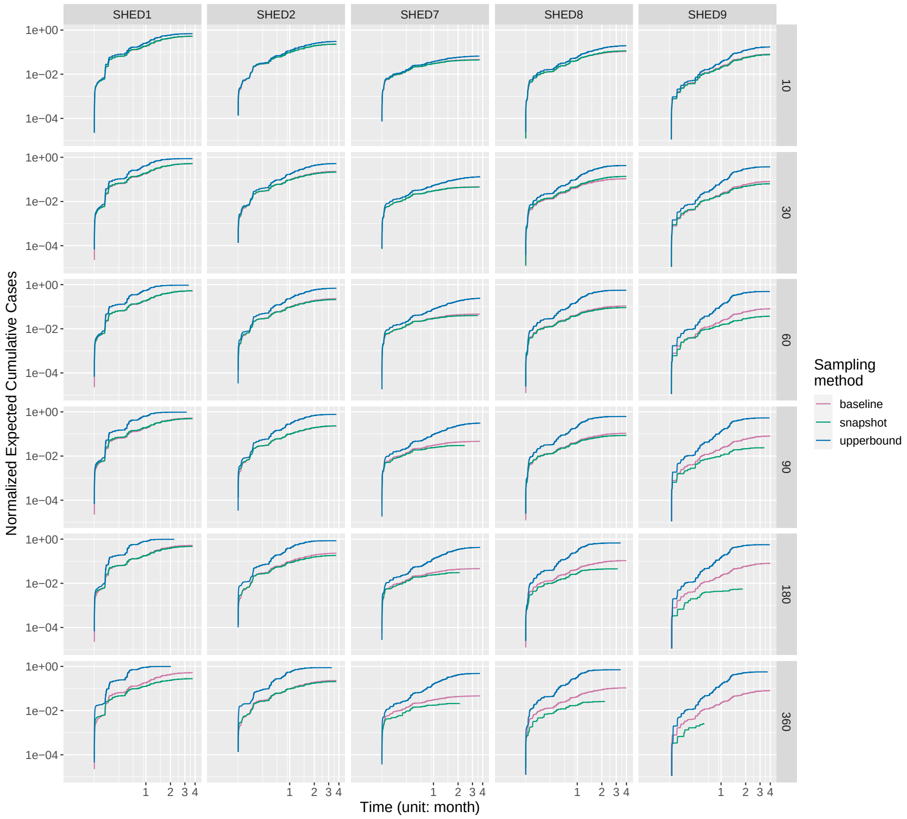

(C) pertussis

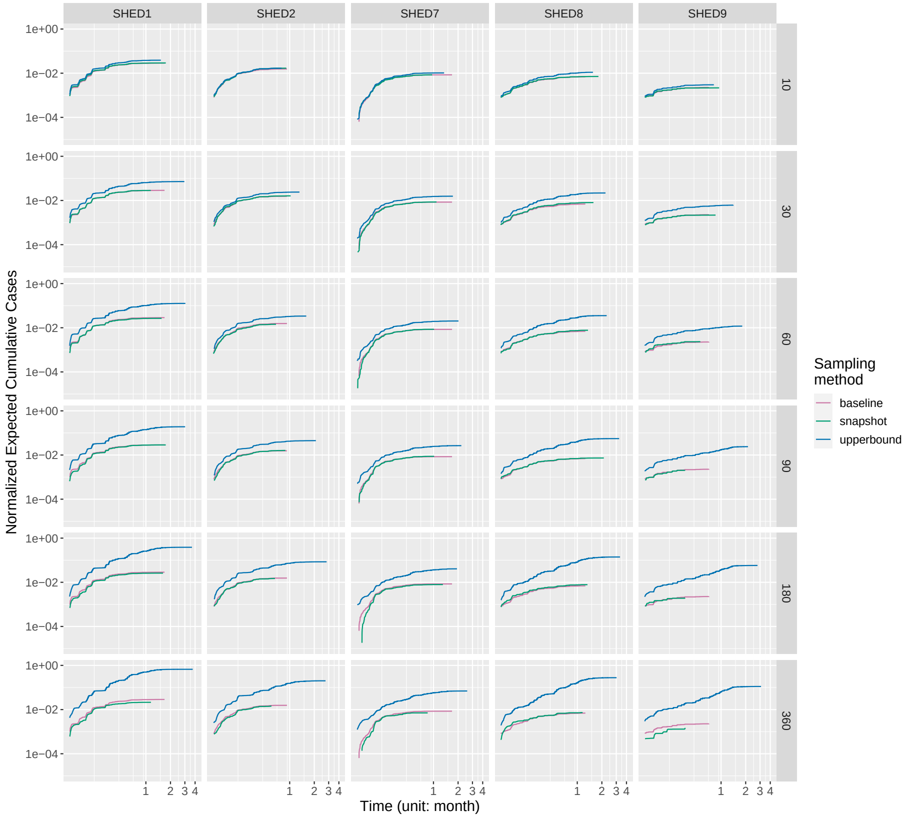

(D) MERS

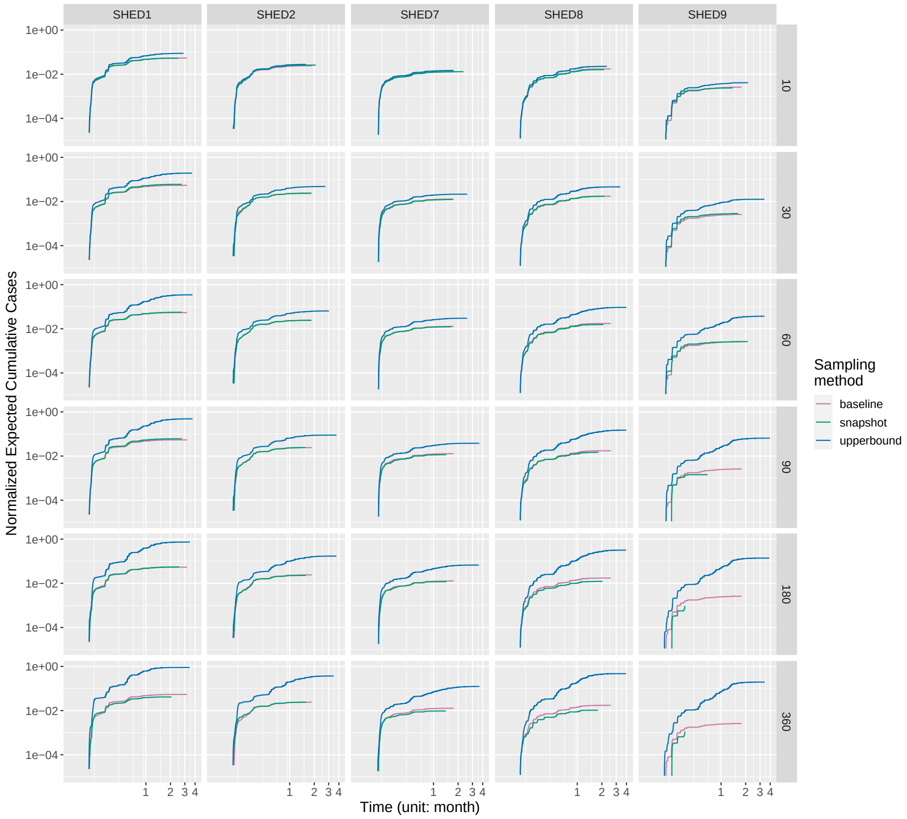

(E) fifth

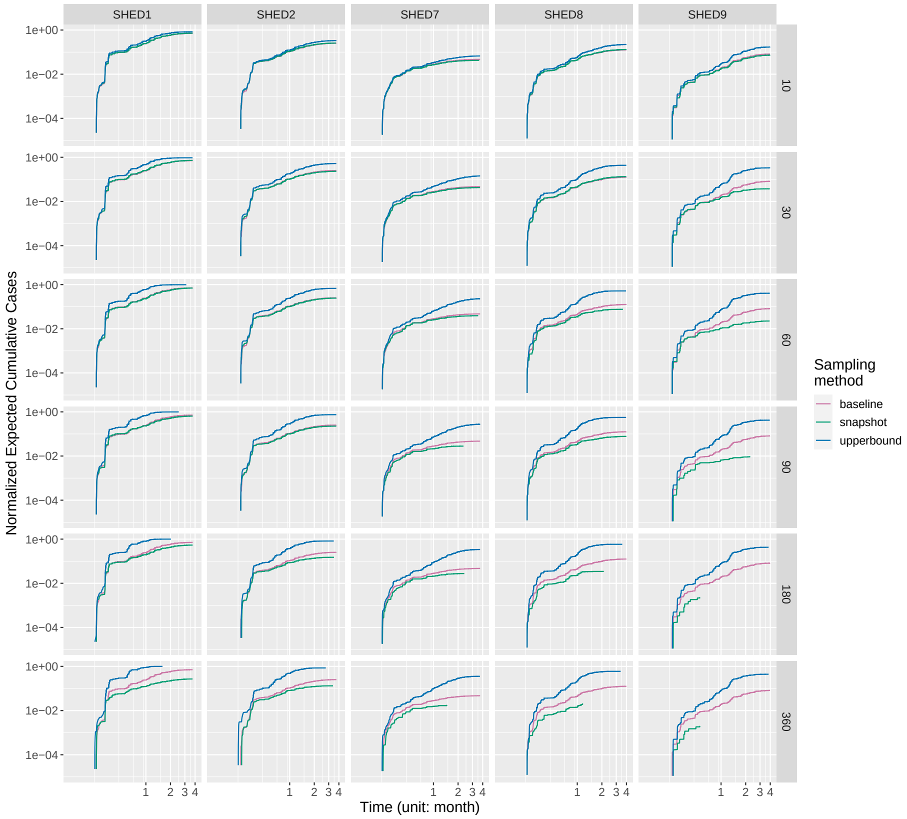

(F) chickenpox

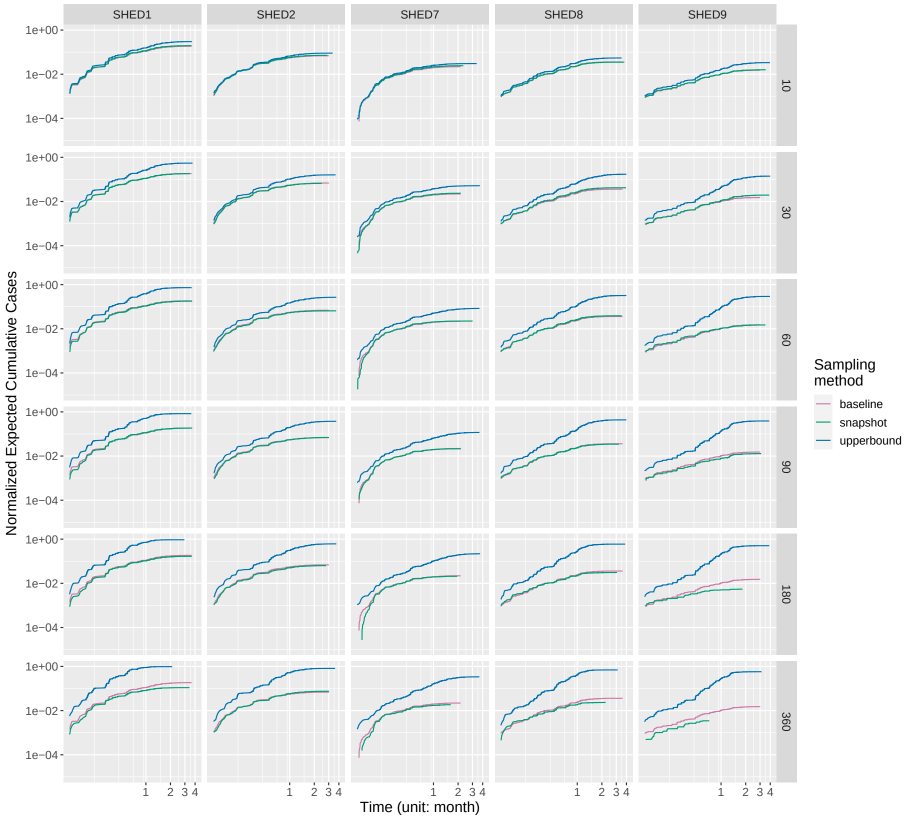

(G) diphtheria

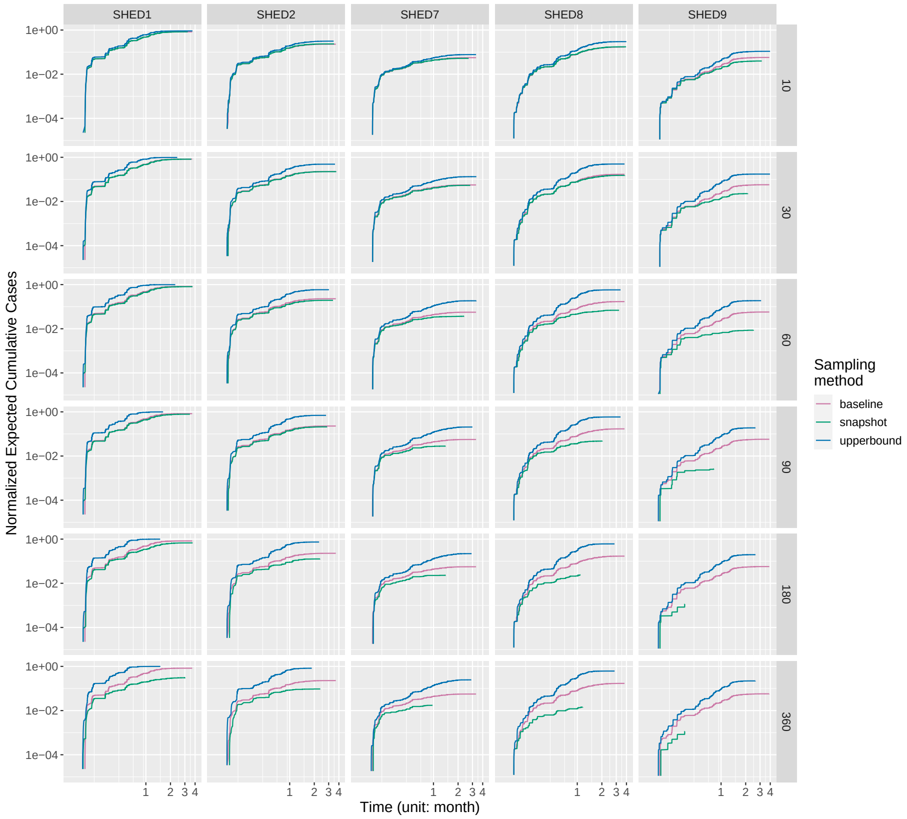

(H) measles

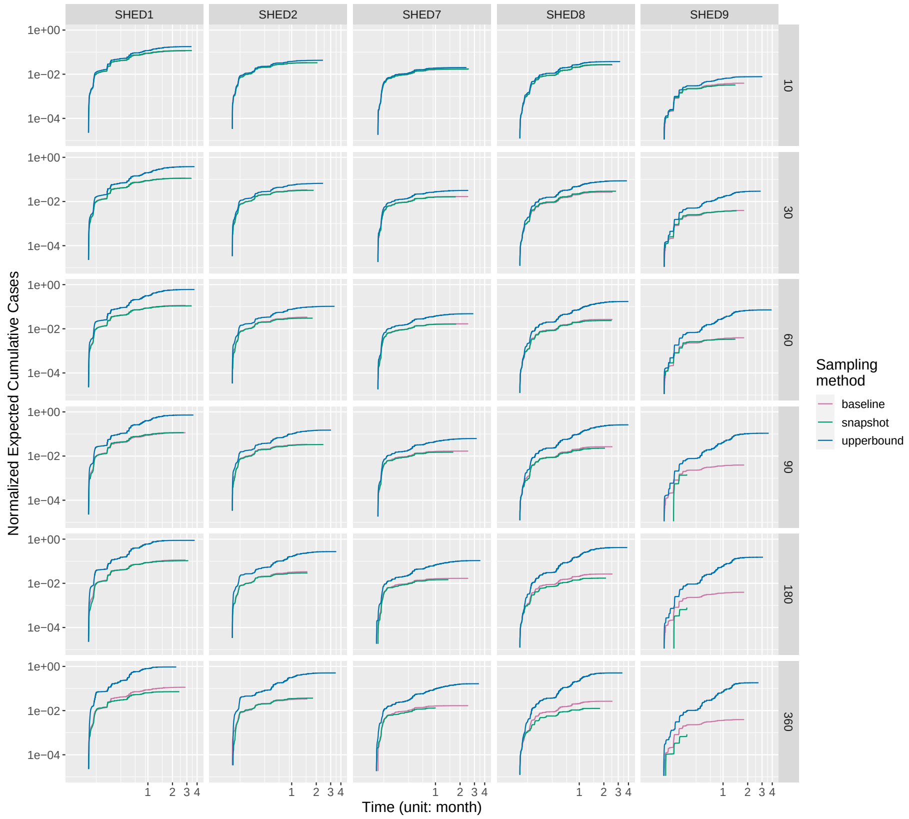

(I) COVID-19 Wild Type

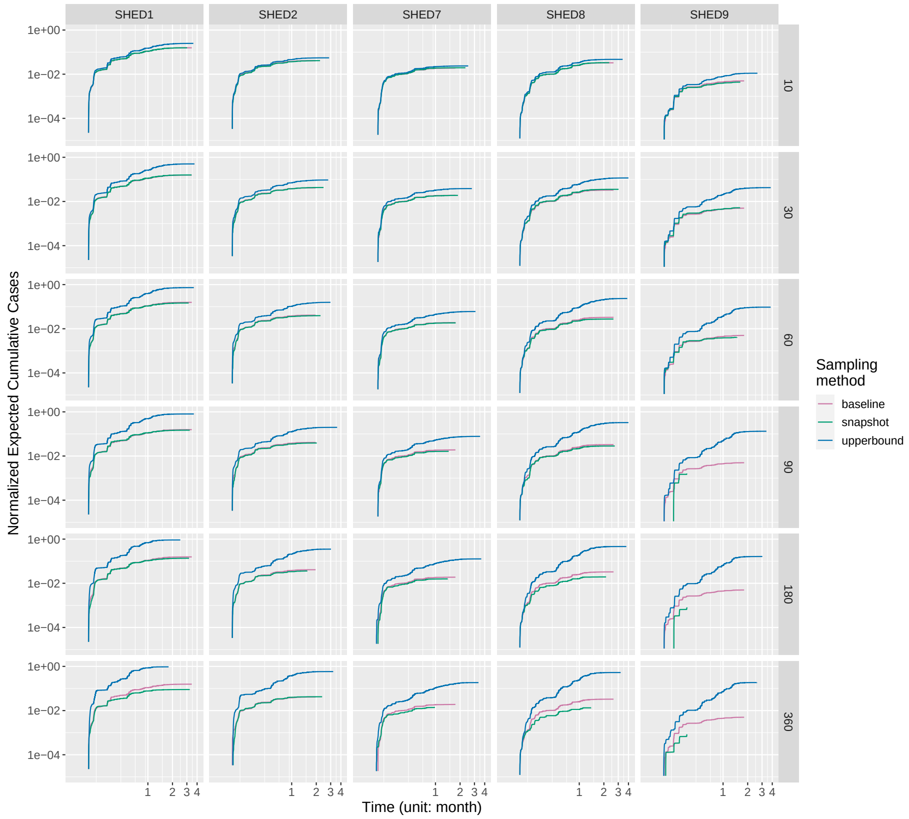

(J) COVID-19 Alpha Variant

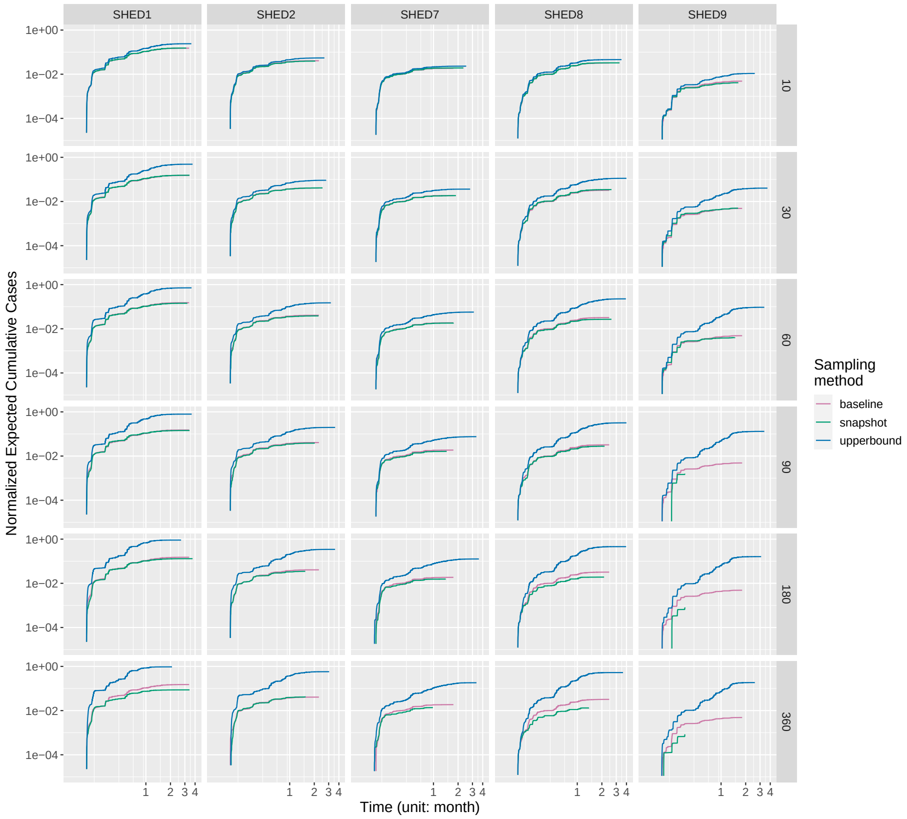

(K) COVID-19 Beta Variant

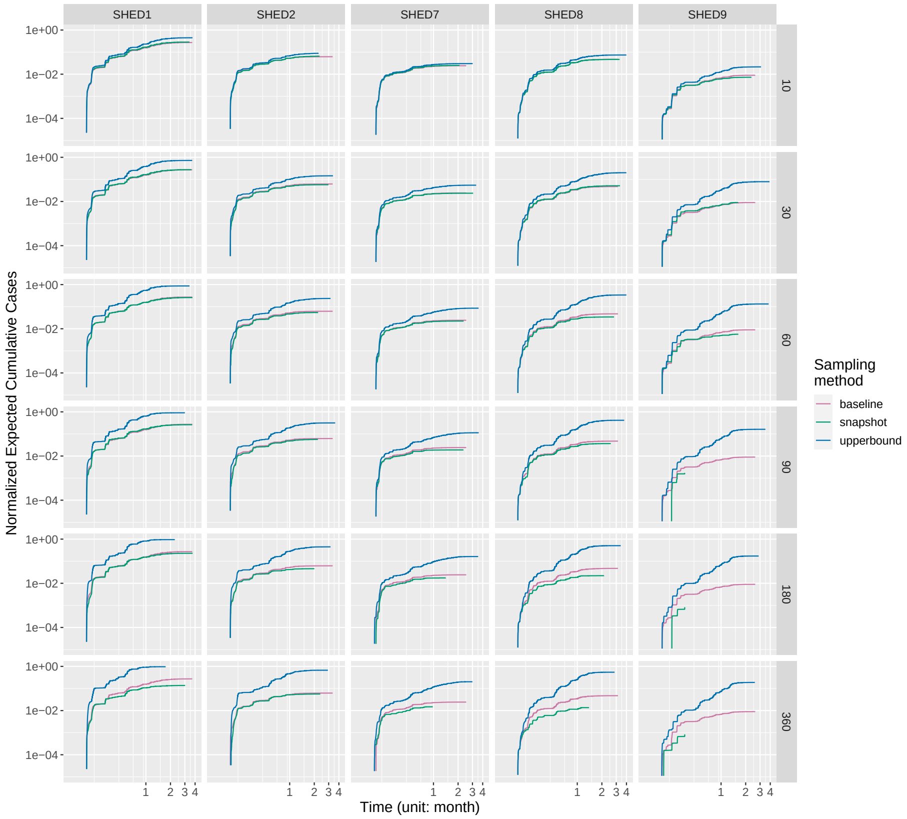

(L) COVID-19 Delta Variant

Fig 10: Comparison of outbreak timing

The normalized expected cumulative cases (NECC) estimates the expected cumulative cases as a fraction of its underlying population size at time t, given the disease, underlying population, sampling method, and sampling interval. We organized NECC over time as colored curves in a grid of cells for each disease, with the underlying population in columns and sampling interval in rows. Each cell plots three NECC curves of the baseline (red), downsampled with the `Snapshot` method (green), and downsampled with the `Upperbound` method (blue). Each NECC curve plots NECC (y-axis) over time in the unit of months (x-axis). Both the x-axis and the y-axis are $\log_{10}$-scaled. The origin of each plot is at $\left(0.5\,\text{weeks},\ 10^{-5}\right)$. Minor ticks on the x-axis(lighter vertical strips) mark 1, 2, 3, and 4 weeks before the first month, then every half month (15 days). NECC curves have different periods (on the x-axis) because they either lack proximate contact from infectious to susceptible or run out of the susceptible. If a NECC curve ends early because of running out of susceptible, its NECC value will stop at one on the y-axis. Steeper NECC curves imply faster growth in cumulative cases. We selected four representatives in the main text; here are the remaining eight out of the twelve in total.

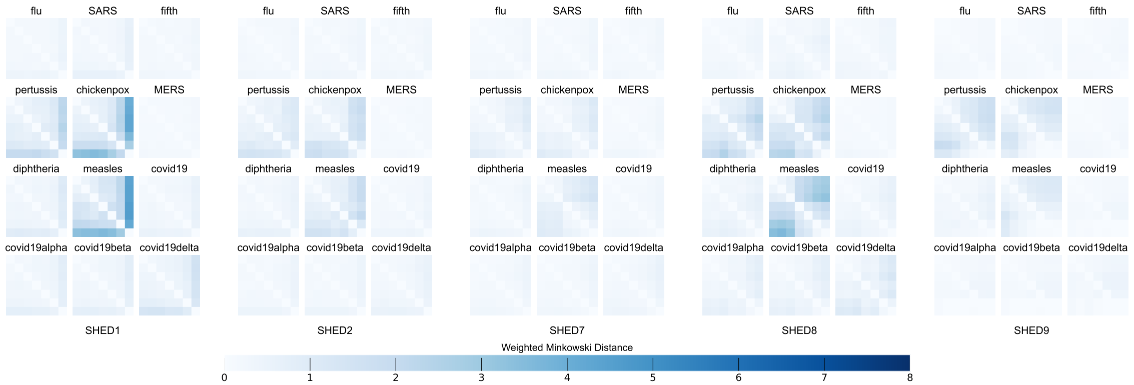

(A) `Snapshot`

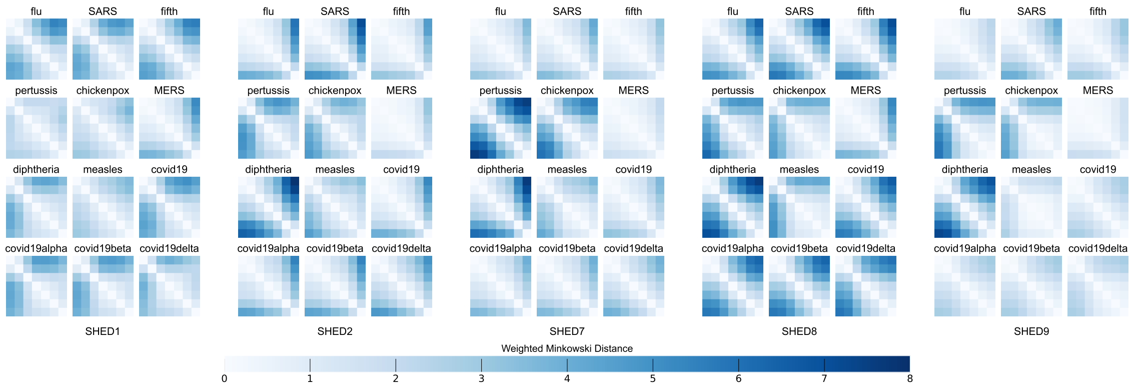

(B) `Upperbound`

Fig 11: Distance matrix of infection pairs

Two grids of two dimensional (2D) histograms visualize matrices of pairwise weighted-Minkowski distances of frequencies of infections pairs given downsampling methods, disease, and sampling frequencies for underlying populations. All histograms share a color-scale as shown in the legend, with the color shifting from lighter to darker with the increasing degree of dissimilarity. For each matrix, starting from its top left corner, inter-observation intervals are arranged in ascending order—$\xi = 5, 10, 30, 60, 90, 180, 360$—horizontally from left to right and vertically from top to bottom. We found `Snapshot` is better at preserving consistent frequencies of infection pairs, particularly with an observation frequency higher than once per half-hour, except for higher R_0 diseases in "diffuse" communities, such as chickenpox and measles in SHED9. For lower R_0 diseases (flu), particularly in "closer" communities like SHED1, the `Snapshot` method have weighted-Minkowski distance less than 2 even between the observation frequencies of 5-minute and 360-minute.

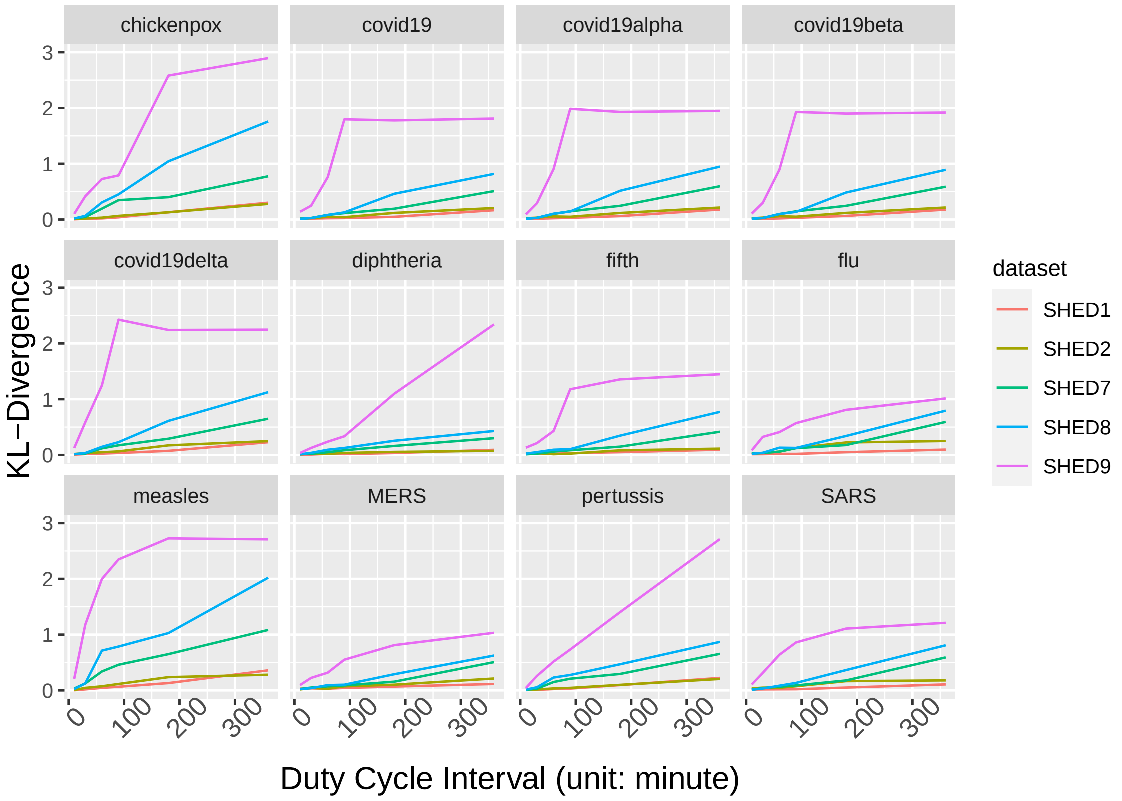

Fig 12: Kullback-Leibler divergence of individual infection risk with the `Snapshot` method

The deviation of individual infection risks as sample interval ξ increases is visualized as a grid of line plots. Every cell of the grid corresponds to a specific downsampling method η (row), disease $\mathcal{M}$ (column). Within each cell, there are five lines with distinct colors; each color denotes a corresponding underlying population V. Each colored line is formed by sequentially connecting adjacent points with straight lines. There are six points for each colored line, each associated with a sample interval in the order of $\xi_+ = 10, 30, 60, 90, 180, 360$, denoting the KL-divergence of the baseline $\boldsymbol{\rho}(\mathcal{M}, \mathcal{D}_{\xi_0,\eta})$ from $\boldsymbol{\rho}(\mathcal{M}, \mathcal{D}_{\xi_+,\eta})$ with the sample interval ξ_+.

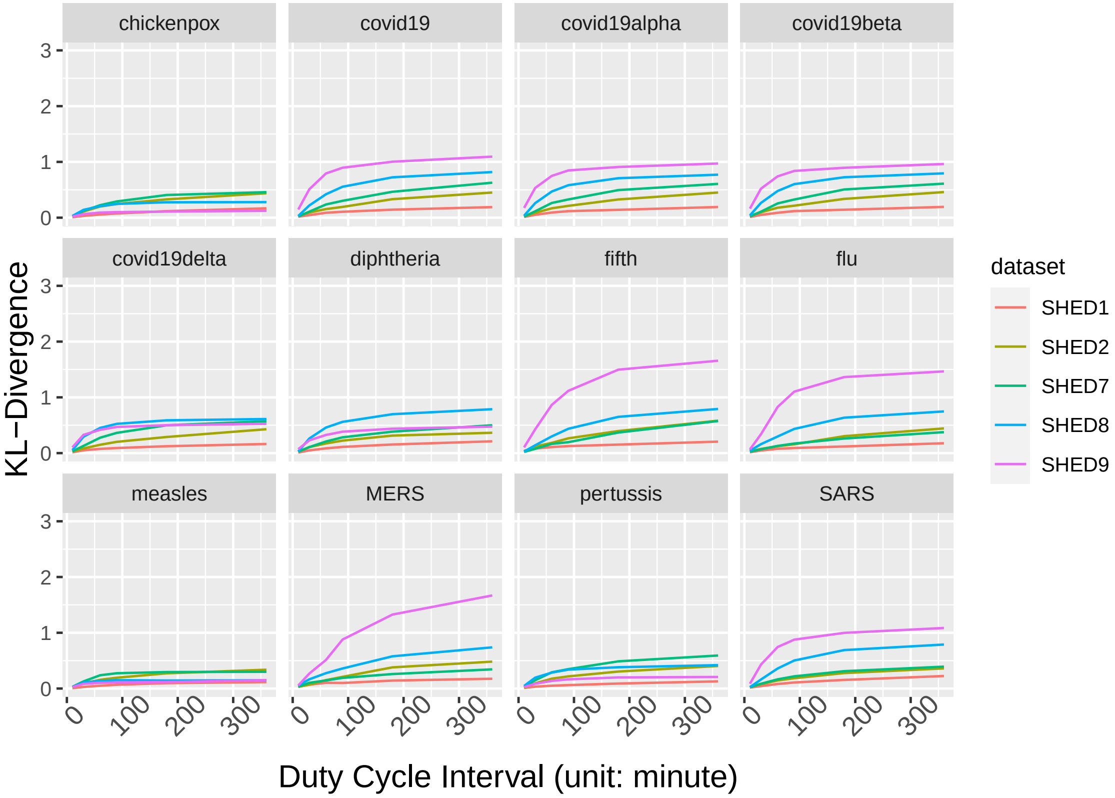

Fig 13: Kullback-Leibler divergence of individual infection risk with the `Upperbound` method
See the caption of Fig 12.

References

1. National Center for Immunization and Respiratory Diseases (NCIRD), Division of Viral Diseases. About chickenpox: Transmission; April 28, 2021. Available from: https://www.cdc.gov/chickenpox/about/transmission.html [cited August 25, 2022].

2. World Health Organization. Tracking SARS-CoV-2 variants; 2022.

3. Campbell F, Archer B, Laurenson-Schafer H, Jinnai Y, Konings F, Batra N, et al. Increased transmissibility and global spread of SARS-CoV-2 variants of concern as at June 2021. Eurosurveillance. 2021;26(24):2100509. doi:10.2807/1560-7917.ES.2021.26.24.2100509.

4. National Center for Immunization and Respiratory Diseases (NCIRD), Division of Viral Diseases. About diphtheria: Causes and spread to others;. Available from: https://www.cdc.gov/diphtheria/about/causes-transmission.html [cited August 25, 2022].

5. Young NS, Brown KE. Parvovirus B19. New England Journal of Medicine. 2004;350(6):586–597. doi:10.1056/NEJMra030840.

6. National Center for Immunization and Respiratory Diseases (NCIRD), Division of Viral Diseases. About flu: How flu spreads; August 27, 2018. Available from: https://www.cdc.gov/flu/about/disease/spread.htm [cited August 25, 2022].

7. National Center for Immunization and Respiratory Diseases (NCIRD), Division of Viral Diseases. Transmission of measles; November 5, 2020. Available from: https://www.cdc.gov/measles/transmission.html [cited August 25, 2022].

8. National Center for Immunization and Respiratory Diseases (NCIRD), Division of Viral Diseases. About MERS: Transmission; August 2, 2019. Available from: https://www.cdc.gov/coronavirus/mers/about/transmission.html [cited August 25, 2022].

9. National Center for Immunization and Respiratory Diseases (NCIRD), Division of Bacterial Diseases. About pertussis: Causes and transmission; August 7, 2017. Available from: https://www.cdc.gov/pertussis/about/causes-transmission.html [cited August 31, 2022].

10. Centers for Disease Control and Prevention, Department of Health and Human Servcies. Fact sheet: Basic information about SARS;. Available from: https://www.cdc.gov/sars/about/fs-SARS.pdf [cited August 31, 2022].

11. Barabási AL, Bonabeau E. Scale-free networks. Scientific American. 2003;288(5):60–69. doi:10.1038/scientificamerican0503-60.

12. Watts DJ, Strogatz SH. Collective dynamics of 'small-world' networks. Nature. 1998;393(6684):440–442. doi:10.1038/30918.

13. Hashemian M, Qian W, Stanley KG, Osgood ND. Temporal aggregation impacts on epidemiological simulations employing microcontact data. BMC Medical Informatics and Decision Making. 2012;12(1):132. doi:10.1186/1472-6947-12-132.

14. Welch BL. The generalization of *Student's* problem when several different population varlances are involved. Biometrika. 1947;34(1-2):28–35. doi:10.2307/2332510.

15. Bland JM, Altman DG. Multiple significance tests: the Bonferroni method. BMJ. 1995;310(6973):170. doi:10.1136/bmj.310.6973.170.

16. Lumley T, Diehr P, Emerson S, Chen L. The importance of the normality assumption in large public health data sets. Annual Review of Public Health. 2002;23(1):151–169. doi:10.1146/annurev.publhealth.23.100901.140546.

17. Sawilowsky SS, Blair RC. A more realistic look at the robustness and Type II error properties of the t-test to departures from population normality. Psychological Bulletin. 1992;111(2):352–360. doi:10.1037/0033-2909.111.2.352.

18. Friedman M. The use of ranks to avoid the assumption of normality implicit in the analysis of variance. Journal of the American Statistical Association. 1937;32(200):675–701. doi:10.2307/2279372.

19. Friedman M. A correction: The use of ranks to avoid the assumption of normality implicit in the analysis of variance. Journal of the American Statistical Association. 1939;34(205):109. doi:10.2307/2279169.

20. Prentice M. On the problem of m incomplete rankings. Biometrika. 1979; p. 167–170. doi:10.2307/2335259.

21. Tugwell P, de Savigny D, Hawker G, Robinson V. Applying clinical epidemiological methods to health equity: the equity effectiveness loop. BMJ. 2006;332(7537):358–361. doi:10.1136/bmj.332.7537.358.
