# Supplementary material for: Impacts of observation frequency on proximity contact data and modeled transmission dynamics
Source: PLoS Comput Biol. 2023 Feb 27;19(2):e1010917. doi: 10.1371/journal.pcbi.1010917 (PMC9997969; doi:10.1371/journal.pcbi.1010917)
Supplement: Attachment — Submitted filename: Review Report_PCOMPBIOL-D-22-00711.pdf [file pcbi.1010917.s003.pdf]

Report on "Impacts of observation frequency on proximity contact data and modeled transmission dynamics"

In this paper, authors studied the impact of observation frequency and sampling methods on the dynamics of proximity contact networks and transmission of 12 different communicable diseases. They collected the proximity contact data with varying sensing regimes and studied the impact of varying regimes on network structure and population-level and individual-level simulation models. They used two different downsampling methods to further investigate the finding regarding the attack rates, individual infection risks and outbreak timing from simulation outputs. They reconstructed SEIR model to consider the individual-level population and used SEIR-ABM model to perform the simulation for 12 different communicable diseases. The findings provide the empirical basis for guidelines to perform better data collection. The paper contains clean and granular longitudinal data and interesting results. However, the flow of the paper is not as per the PLOS Computational Biology journal, for example the results are presented before presenting methods. Please revise thoroughly. There are some minor comments:

- page 3, last paragraph, lines 81-82 → It would be helpful to provide reference and the schematic diagram of the model.

- page 5, lines 145-147 → The sentence is not clear, please revise
    - line 154 → ξ is used first time here and not defined
    - lines 163 – 164 → I think the explanation here got mismatched e.g., diseases (row) and underlying populations are (columns)
    - line 180 → no reference for Welch's t-test

- please provide the detailed legend for all figures in the paper. A figure should be able to stand alone.

- page 7, line 203 → reference for Prentice-modified Friedman test

- page 12, fig. 5 → Please explain why the incidence occurrence time unit is in minute, when the axis shows approximate 35 days interval and goes up to approximate 139 days. It is making figure too dense to easily understand.

- page 14, lines 371-375 → please use numbered points in place of dash.

Thank you and good luck!
