# Supplementary material for: Impacts of observation frequency on proximity contact data and modeled transmission dynamics
Source: PLoS Comput Biol. 2023 Feb 27;19(2):e1010917. doi: 10.1371/journal.pcbi.1010917 (PMC9997969; doi:10.1371/journal.pcbi.1010917)
Supplement: Attachment — Submitted filename: PLOS_CB_rebuttal.pdf [file pcbi.1010917.s004.pdf]

Thank you for considering our paper on the impact of contact measurement scheduling on agent-based disease modelling. The reviewers and editor were unanimous in deciding that the contribution was novel, timely and within the scope of the journal. Both the editor and reviewers made valuable suggestions and requested changes ranging from the minor to substantial. We would like to express our gratitude to the reviewers for their work in helping make this paper stronger. Presenting large, complex analyses of high dimensional parameter spaces can be challenging, and having a fresh set of expert eyes examine the presentation and make suggestions is valuable and appreciated.

For the most part, we have revised the manuscript to reflect the feedback from the editor and reviewers. Where we felt such feedback was ill-advised, we have provided clear reasoning as to why in this letter. We have separated every specific comment made by each reviewer and the editor, even if that comment was made in a narrative form, and have responded inline. For the remainder of this letter, comments are provided in italics, and responses in plain text immediately below. We hope this fulsome but slightly pedantic format will help speed the review process for the next round.

Editor

I agree with the reviewer comments that this is potentially a valuable contribution and that its focus on combining social and mobility data is a meaningful advance.

Thank you for your kind appraisal.

While I agree with the Reviewer comment that comparing to empirical data would strengthen the study, but I think there's a path forward without necessarily adding in an empirical comparison. I will leave that decision up to the authors.

We agree that comparing with empirical data would make the study stronger; however, the paper is already highly complex, as both reviewers noted, and adding an additional manipulation and comparison would make the paper too many things at the same time. That being said, comparison with empirical data is important. We have added several sentences to the future work section outlining why this is important and how it should be accomplished.

However, I think a bigger concern is around the data sharing. There's already a lot of work on longitudinal contact networks and they almost always provide more granular data. Because the actual model and results are not in and of themselves novel, the value of this paper is strongly driven by the data themselves.

The results are strongly driven by the data, but we would contest the implication that the study presented here is not novel without novel data. While researchers have used contact data to drive epidemiological simulations, in both agent based and compartmental models, no one has examined how disease parameters and contact measurement density impact the results of simulation. This is important for informing both the required sampling rate of future data collection studies, and the minimum temporal granularity for characterizing network change during simulation. We have attempted to reframe the contribution to be clearer on these points.

That you can only share aggregated data because of what the participants agreed is a study design issue and significantly lowers the future value of the study. I appreciate that it's harder

work to share longitudinal data, but SocioPatterns has done it, Salute has done it, and researchers like Sune Lehmann have done it. Can the authors provide any mechanism for future authors to obtain access to the more granular data (in this case I mean beyond simply saying, "available upon reasonable request"). If the authors cannot address this data availability issue, they should provide strong justification for how this advances from existing work on longitudinal studies without providing access to the data.

Data sharing is a challenging topic, as it puts in tension two value systems: the rights of the participant, and the value of open data. This is confounded by the SHED studies data itself, which as mentioned in the paper, contains substantially more data streams than Bluetooth contact and battery state. In particular, the inclusion of GPS and location data is problematic. Sufficiently high velocity GPS data can allow a skilled practitioner to determine salient information about participants, such as place of work, residence, and daily habits. From this information it is almost trivially easy to re-identify participants, a clear violation of most research ethical frameworks. We have circumvented this issue by committing to our own research ethics board, and to the participants, that any researcher who accesses the data must commit in writing to not cross reference any of the data with external data containing personal information (for example, a phone book). It is difficult to administer this kind of commitment for a public repository, and essentially impossible to enforce it, preventing full public disclosure of the data. Because the participants did not give prior informed consent for sharing of a subset of the data, we are also bound by ethics to not disclose the contact patterns, even though the potential for re-identification is small. The associated challenges are multiplied by the diversity and age of the SHED studies providing the basis for this work.

However, there is a mechanism for accessing the data. Researchers at institutions with research ethics boards must make an application to their board to use this data. Upon successful completion of that process, those researchers must make an application to our research ethics board for data sharing and secondary data use. Typically this would be sponsored by one of the co-authors. If that process completes successfully, then researchers would be granted access to the data. As this process is somewhat involved, we employed the ‘available upon reasonable request’ euphemism. We have adapted the data availability section to reflect the full process.

Reviewer #1

In this paper, authors studied the impact of observation frequency and sampling methods on the dynamics of proximity contact networks and transmission of 12 different communicable diseases. They collected the proximity contact data with varying sensing regimes and studied the impact of varying regimes on network structure and population-level and individual-level simulation models. They used two different downsampling methods to further investigate the finding regarding the attack rates, individual infection risks and outbreak timing from simulation outputs. They reconstructed SEIR model to consider the individual-level population and used SEIR-ABM model to perform the simulation for 12 different communicable diseases. The findings provide the empirical basis for guidelines to perform better data collection.

Thank you for the succinct synopsis of our work.

The paper contains clean and granular longitudinal data and interesting results.

Thank you for noting the utility of our results.

However, the flow of the paper is not as per the PLOS Computational Biology journal, for example the results are presented before presenting methods. Please revise thoroughly.

Based on our reading of the PLOS CB guidelines we were under the impression that results should come before the methods, even though that makes reading a paper where there is novelty in the methods difficult. Upon rereading the guidelines, we realised that the results before methods format is preferred, but not required. This was a relief as we agree that putting the methods first will make the paper much more comprehensible. We have moved the methods section earlier in the paper, as suggested by the reviewer.

There are some minor comments:

• page 3, last paragraph, lines 81-82 → It would be helpful to provide reference and the schematic diagram of the model.

• page 5, lines 145-147 → The sentence is not clear, please revise

o line 154 → ξ is used first time here and not defined

o lines 163 – 164 → I think the explanation here got mismatched e.g., diseases (row) and underlying populations are (columns)

o line 180 → no reference for Welch's t-test

• please provide the detailed legend for all figures in the paper. A figure should be able to stand alone.

• page 7, line 203 → reference for Prentice-modified Friedman test

• page 12, fig. 5 → Please explain why the incidence occurrence time unit is in minute, when the axis shows approximate 35 days interval and goes up to approximate 139 days. It is making figure too dense to easily understand.

• page 14, lines 371-375 → please use numbered points in place of dash.

All minor comments have been addressed as requested in place and the schematic diagram of the model in the supplemental material. Thank you for your suggestion for improving the understandability of this contribution.

Reviewer #2

The authors present a useful and practical work for contact tracing spread of infectious diseases, especially while the COVID-19 pandemic. There are many countries started to use auto contact tracing apps via smartphones. This study is an interesting work to evaluate the data sampling approaches and is helpful in preparation for potential outbreaks in the future.

Thank you for noting the utility of our work.

The study consists of several important components in infectious disease modeling such as contact network and agent-based SEIR model, and practically uses individual traced data to investigate the impact of the network and model given 2 downsampling methods with lower sampling frequencies. The authors apply the 5 sampled networks on 12 diseases and compared 2 downsampling methods with 7 frequencies (1 baseline and 6 alternatives).

Thank you for the summary of our methods, and for making evident an appreciation for the scale of the parameter space explored by the paper.

- Even though the sampling population could be too small and be biased due to its closed population in the university but this is still useful to demonstrate the methodology of the study.

Agreed. The findings are meaningful, but potentially biased by the population. However, the methodology should hold if a larger sample were available for analysis. We expect that the broad conclusions and ordering of impact would remain largely unchanged, but would not be surprised if the magnitude of the effects shifted with population. We have attempted to reword the limitations and future work section to make this clearer.

- Overall, the manuscript is written nicely and some comments are listed in the followings.

Thank you for your supportive assessment.

- recommended study about contact tracing app in the UK: [Wymant, C., Ferretti, L., Tsallis, D. et al. The epidemiological impact of the NHS COVID-19 app. Nature 594, 408–412 (2021). https://doi.org/10.1038/s41586-021-03606-z]

We have added this citation to the related work in page 3, line 43–45.

The introduction of the manuscript is smoothly written and describes the objectives with three research questions clearly.

- Page 3/30: The authors can add a citation of Table 1 while mentioning the reproduction numbers.

We have put the forward reference in.

- It would be recommended to answer these questions more systematically in Discussion.

We appreciate the reviewer's feedback, and have reworked the Discussion to explicitly address these research questions.

The methods and results need to be organized better and more clear description or systematic flow is needed for readers to understand better without going back and forth between Methods and Results.

As noted by Reviewer 1 and in contrast to our original reading of the PLOS CB guidelines, it is not necessary to place the methods after the results in PLOS CB. Reflecting this, we have reordered the paper; we hope that having the methods now placed before the results will significantly mitigate these challenges. To further address the identifier concerns, we have added sentences in the results section clearly indicating the subsection of the methods describing the corresponding experiment being presented.

- The authors can consider moving some part of the Methods into the main text or/and to add more description for example while explaining the two downsampling methods in 1st paragraph of the results.

As noted above, we have moved the entire methods section before the results, addressing this comment. We agree that this improves the clarity of the presentation.

- Table 1 is a good summary of all 12 diseases in this study. Adding a short paragraph in Method to describe the diseases (such as Fifth disease caused by Parvovirus B19 and much more common among children than adult) would be recommended. This type of information is important while applying the methodology using data collected in university.

We again thank the reviewer for the guidance offered. While we explored the inclusion of a paragraph, because of the dozen diseases /pathogens explored we included it in the text to be unwieldy, and disruptive to the flow of the paper. We have instead sought to address this concern by including a table with one to two sentence descriptions, as requested, to the supplemental material. We have further placed a reference to that table in Table 1.

For discussion, the network structures in different age groups and communities (household vs school) could be a key factor in transmission.

We have added a note on this point to the future work in conjunction with the editor's request for a clearer statement on future work. Given the relatively small and biased sample, we cannot make conclusive decisions about the demographics of transmission.

The pathogen or variant types can be mentioned there. "disease/pathogen" can be replaced by simple "disease".

Changed as requested.

- Table 1: 2nd row, replace "COVID-19" by "COVID-19 wild type".

Changed as requested.

Cumulative cases and ECDFs

- It is quite difficult to read all the figures especially figures 1 to 4. There is too much information and the font is too small. The authors can select some of the presented results (such as the section of "Outbreaks and outbreak timing") and put the others in the supplementary. Also, the figures 1 and 2 can be combined in one and so for figure 3 and 4 (for example using color and shape for sampling interval and downsampling method, respectively).

There is always a chicken and egg debate around complex figures. On the one hand, complex figures impede comprehension. On the other hand, simplified figures raise the possibility of the authors being accused of selective result disclosure. We have provided the simplified figures as requested, and moved the complete figures to supplementary materials. However, we leave the final decision on whether this improves or detracts from the paper to the editor.

- The section of "Outbreaks and outbreak timing" can be part of "Cumulative cases".

Sections have been combined as requested.

In figure 5a, it is clear to see that Upperbound method (blue line) slow down the spread while interval increases, but all methods seem to behavior the same in the bottom-left panel. Can you explain this? Would this be related to the stochasticity of the agent-based model?

The Upperbound method (blue line) tends to bloat proximate contacts, resulting in prolonged infectious periods. At any given time, The Upperbound method usually overestimates the cumulative cases compared to the baseline and the Snapshot methods. The Upperbound method is fast in increasing cumulative cases but slower in terms of its progress towards concluding the infection period. Initially, we used the ECDF as a measure because it is automatically normalized between 0 and 1 and is frequently used in network science. We found that it can be tricky to interpret results in forms of ECDF. In this revision, we have developed a new metric, named normalized expected cumulative cases (NECC), which we believe can better reflect the differences related to outbreak timing. We also added explanations to help interpret this phenomenon.

In the description on Page 21/30, 30 simulations per scenario seem to be too low, but I can understand that the output data size is quite large, 85GB.

It was necessary to balance the rigour of the simulation against the size of the resulting file and compute time required for each of the 5 studies and 12 diseases. We selected 30 as a reasonable compromise.

In Figure 5c, while comparing by columns (for example SHED 7 and 9, both diffuse communities), one tends to slow down and one tends to speed up the spread. Would this happen because of the biases of the sampled network data?

As stated above, we used the new metric NECC to avoid confusion in interpreting results of ECDF.

- On Page 5/30: the Xi symbol is not defined until Page 7/30.

Xi is now defined when first presented.

- Figures 1 and 2: what are the blue lines and red dots? More detailed captions in all figures are needed.

We have added detail to the captions.

- The two supplementary spreadsheets might be uploaded as one excel file.

We chose not to upload an excel file, as that is a proprietary format. It is relatively simple for someone who wishes to have both in the same excel file to import two CSV’s as individual sheets.

- Page 10/30: (ECDF) is written twice.

Corrected. Thank you.

Attack rates

- On Page 8/30, Line 5: Should that be the curvy V and normal V in {D, V, M}? The initial infectious individual (curvy V) is not mentioned until the 2nd paragraph.

- The concept of the two V is described in Methods but it is a bit confusing while reading this section.

We have attempted to clarify our use of V. Hopefully having the methods before results will also aid in comprehension.

Infection pairs

- Page 12/30: In the first paragraph, a better description of the resulting values of Weighted Minkowski Distance (Figure 6) and KL divergence (Figure 7) is needed, especially while grouping two types of diseases.

We have added sentences attempting to guide the interpretation of figures 6 and 7.

Would that be associated with the reproduction numbers, as mentioned on Page 13/30? Or also the incubation periods and infectious periods listed in Table 1?

All parameters of the disease impact its transmission, but we expect that the reproductive numbers are the most impactful.

One can also investigate how these three factors affecting the results, for example, all 4 COVID-19 variants share the same incubation periods and infectious periods and the only variable is the R0. This affects the results in Figure 3 but not Figure 4. On the other hand, long and short infectious periods can be compared using pertussis vs measles given both sharing similar R0 and incubation period.

We have added a more nuanced comparison to the discussion to highlight the impact of the various disease parameters.

- Page 12/30: Should the darker/lighter color refers to green/red in Figure 6?

Thank you, indeed the darker/lighter should be reddish/greenish. To make our figure friendly to graytone and readers who are color blind, we recolored the figure from greenish/reddish to lighter/darker blue.

- Figure 6: what is the x-y axis?

Thank you. We have added explanatory text in the long-caption of this figure, explaining the layout of x-y axes for distance matrices.

Individual infection risks

- Page 13/30, is the (Xi_+) symbol the same as (Xi') symbol on page 8/30?

Yes and thank you! We have unified both symbols to (Xi_+).

Reviewer #3

In their paper, the authors present a new method to better combine social information from cellular networks with an epidemiological model to model the spread of various infectious diseases. Indeed, over the past two years, there has been an increased interest in accurately creating models to predict the spread of epidemics, so many researchers across different fields have worked to develop models that can faithfully predict plague outbreaks under various scenarios.

Thank you for recognizing the importance of the motivation underlying our work.

However, this article does not provide enough evidence that an ABM-SEIR, which uses data from mobile devices, is the best method of tracking pandemic spread.

In fact, the paper provides no evidence that an ABM-SEIR model is the best method for tracking a pandemic. This is not the contribution of our paper. Our paper focuses on the impact of sampling rates on ABM-SEIR outcomes, given different diseases. We make no claim as to the relative merit of the various measurement and modelling approaches in this paper. We have attempted to clarify the contribution to make this clearer.

Hence, this paper should focus on fewer pathogens and primarily show how this model can be used to predict an outbreak of COVID-19 in contrast to the SEIR model (or another model)l, which doesn't use such external information.

If we were trying to establish the utility of ABM models versus compartmental models (something already addressed in the literature) this would be an appropriate step. However, the purpose of our work is to examine the repercussions on ABM-SEIR model outcomes of variable measurement frequency, based on disease characteristics. The suggested comparison is interesting, however, and has been added to future work.

In addition, I have several minor comments regarding the presentation of the data in the paper:

While the authors present an innovative method for collecting data that can be used in epidemiological ABM, the paper itself is hard to read, and all the figures are not self-explanatory because the data they contain is so large. Although the authors attempted to include as many pathogens as possible, this resulted in a detailed paper that could have been better organized. Since each calculation contains five data sets, the authors should only present three pathogens per figure. By doing so, the figures will be more readable, and readers will better understand the differences between the different data sets and methods employed in this study. Hence, I think this paper can be better organized, and the figures have to be changed to contain less information (the original figures can use as supplementary material).

Reviewer 2 made a similar comment. We have attempted to simplify the figures with selected results and have moved the existing figures to the supplementary material. However, this does increase the risk that of readers perceiving that the results were selected rather than presented. We leave to the editor the final decision on whether the main body of the paper should include the full results or a only subset.
