# Supplementary material for: Impacts of observation frequency on proximity contact data and modeled transmission dynamics
Source: PLoS Comput Biol. 2023 Feb 27;19(2):e1010917. doi: 10.1371/journal.pcbi.1010917 (PMC9997969; doi:10.1371/journal.pcbi.1010917)
Supplement: Attachment — Submitted filename: PLOS_CB_rebuttal_Revision2.pdf [file pcbi.1010917.s005.pdf]

Thank you for providing further feedback on our paper. The reviewers and editors recognized the improvement made in our submitted revision. Both the editor and reviewers made valuable suggestions and requested a few minor changes. Once more, we would like to express our gratitude to the reviewers for their work in helping make this paper stronger.

For the most part, we have revised the manuscript to reflect the feedback from the editor and reviewers. Where we felt such feedback was ill-advised, we have provided clear reasoning as to why in this letter. We have separated every specific comment made by each reviewer and the editor, even if that comment was made in a narrative form, and have responded inline. For the remainder of this letter, reviewer comments are provided in italics, and responses in plain text immediately below. We hope this fulsome but slightly pedantic format will help speed the review process for the next round.

Editor

The authors have done a nice job with the revision.

Thank you for your kind appraisal.

However, I still would like to see more in terms of data availability. In their response, the authors mention that aggregate data can be made available. In many cases, those aggregate data are likely to be useful for epidemic simulations. Can the authors provide aggregate metrics, e.g., degree distributions over time, measure of network structure like cluster over time, etc.?

SHED datasets used in this manuscript have some descriptive information listed in cited papers. To ease reader comprehension, we have reproduced existing and added additional aggregate metrics of each dataset in the appendix. Aggregate distributions can be found as CSVs in the public code repository. We also added in line 113 that "Descriptive information of these SHED data sets, such as aggregate distributions, can be found in the S1-Appendix and the code repository" to inform readers of the public availability of such information.

Reviewer #2

There are lots of places improved in this revised version. The figures are simpler and easier for readers to understand where to focus on. The results of using NECC show consistence between scenarios.

Thank you for recognizing our improvements.

The article is more organized and readable by placing Results after Methods. However, most of the 1st paragraphs in subsections of Results should be placed in Methods to explain and define all symbols and notations. For example, Lines 475-502 on Page 14/31 should be Methods on Page 10/31 (note that eta is not defined right after first presented on Line 482. It can be mentioned on Page 6 or 7). The same on

Lines 540-549 on Page 15/31 should be Methods on Page 10/31. Lines 615-627 on Page 20/31 are also Methods where notation eta and xi_+ should be predefined.

We have reorganized the contents in subsections of Results into Methods, moving explanations and definitions of all symbols and notations into Methods.

Some minors:

- 'basic reproductive number' instead of 'base reproductive number' on Line 246 Page 7/31.

Thanks for pointing out that incorrect use of "base reproductive" that anomalously appeared on page 7 of the revised document. We have corrected this to "basic reproductive number", per the remainder of the document and standard convention.

- Line 637: delete 'two'.

Thanks for pointing that out, we have corrected it.

- COVID-19 wild type is added in the table but not in the main text for example on page 5 and 7.

Thanks for pointing that out, we have corrected it.

- While mentioning the time interval between sampling t_i in [Xi i, Xi i + Xi), it can be written as i Xi, (i+1) Xi to avoid mixing up with index i on for example Line 174 on Page 5/31.

Thanks for the suggestion, we have it rewritten to avoid confusion.

Reviewer #3

As a result of the authors' revisions, the article has been improved.

Thanks for recognizing our improvements.

Having the methods part appear prior to the results does improve understanding of the article and simplifies the paper. Moreover, the figures are presented better in the current version of the article than in the original (Fig. 2 is still unreadable).

We have changed Fig.2 from landscape into portrait, such that each subfigure becomes larger. Meanwhile, to reduce the number of scattered points, we only show scattered points related to Duty Cycle Intervals of 10, 60, and 360 minutes.

However, In my opinion, an article presenting an epidemiological model for predicting the rate of pandemic spread must be compared to the actual data in some way. In the end, epidemiological models are designed to monitor the spread of a pandemic and to facilitate informed decision-making. The fact that after two and a half years, the coronavirus still exists to a different extent in most countries of the world shows us that we should not take the global pandemic lightly, which emphasizes the necessity for epidemiological models that are as precise as possible. As a result of frequent changes in how the Corona pandemic spreads (for example, the introduction of new variants and the effectiveness of vaccinations against infection/severe morbidity), and on the other hand, there has been a change in

epidemic control policy (i.e., there are almost no lockdowns in the world as compared to two years ago), most models produced mediocre results in predicting the spread of the pandemic and have been criticized regularly. Therefore, I find that an article that ultimately presents an epidemiological model for predicting the rate of spread of an epidemic must be compared in one way or another to the real data. Furthermore, it would be necessary to convince the reader that this model, which uses cellular data, has an advantage over epidemiological models that do not utilize this information because the information required for this model is not publicly available in all countries.

Close-proximity contact data informs transmission models to achieve goals such as estimating parameters, simulating (or predicting) dynamics, and exploring “what-if” (or counterfactual) scenarios. The study described in the manuscript found that when informing transmission models with close-proximate contact data, the observation frequency of the collected close-proximate contact data matters. Our manuscript informs researchers when using transmission models with close-proximate contact data rather than proposing a specific model to predict COVID-19 or other pathogens. Researchers are proposing approaches for transmission models to benefit from close-proximate contact data, along with progress of research on close-proximate contact data. Transmission models have various purposes, such as understanding the transmission pathways, projection, current state estimation, or evaluation of the impact of measures. In addition, real-world data, such as the COVID-19 incidence reports, may vary by regional definition and data collection approaches, collecting coupled close-proximate contact data and health data is an intensive process and requires additional ethical guarantees. While the suggestions made by the reviewer are certainly valid, they each represent contributions in their own right, as subjects of their own detailed studies and peer-reviewed manuscripts. The contributions suggested by the reviewer would be valuable additions to the literature, but are distinct from the contribution we make in this work.
